# Inhibition of PGC1β-dependent mitochondrial biogenesis enhances EGFR-targeted therapy in lung cancer

**DOI:** 10.1038/s44321-026-00493-7

**Published:** 2026-07-30

**Authors:** Zhen Chen, Dongsheng Wang, Songqing Fan, Qiming Wang, Yong Huang, Pan Du, Shidong Jia, Suresh S Ramalingam, Shi-Yong Sun

**Affiliations:** 1 Departments of Hematology and Medical Oncology, Emory University School of Medicine Atlanta, Atlanta, GA USA; 2 Department of Pathology, The Second Xiangya Hospital, Central South University, Changsha, China; 3 Department of Internal Medicine, The Affiliated Cancer Hospital of Zhengzhou University & Henan Cancer Hospital, Zhengzhou, China; 4 Institute of Cancer Research, Henan Academy of Innovations in Medical Science, Zhengzhou, China; 5 Predicine, Inc, Hayward, CA USA; 6 Winship Cancer Institute of Emory University, Atlanta, GA USA

**Keywords:** Cancer, Metabolism, Respiratory System

## Abstract

Third-generation EGFR tyrosine kinase inhibitors (EGFR-TKIs), including osimertinib, show robust clinical efficacy in EGFR-mutant (EGFRm) non-small cell lung cancer (NSCLC), yet acquired resistance remains inevitable. Here, we demonstrate that osimertinib and other EGFR-TKIs suppress PPARGC1B expression and its regulated mitochondrial biogenesis in EGFRm NSCLC cells through a previously unrecognized FOSL1/AP-1-mediated transactivation mechanism. Upon acquisition of osimertinib resistance, PPARGC1B expression and its encoded protein PGC1β rebound and become refractory to osimertinib-mediated suppression. Enforced overexpression of PPARGC1B confers resistance to osimertinib in sensitive EGFRm NSCLC cells, whereas PPARGC1B knockdown restores drug sensitivity in resistant cells. Moreover, combining osimertinib with the mitochondria-targeting agent CPI-613 synergistically suppresses mitochondrial biogenesis, induces apoptosis, and inhibits the growth of osimertinib-resistant cells and tumors. Collectively, these findings identify PGC1β-dependent mitochondrial biogenesis as a critical determinant of therapeutic response to osimertinib and suggest co-targeting mitochondrial metabolism as a potential strategy to overcome acquired resistance in EGFRm NSCLC.

## Synopsis

Acquired resistance to osimertinib remains inevitable in EGFR-mutant NSCLC. PGC1β-dependent mitochondrial biogenesis emerges as a key determinant of therapeutic response, providing a rationale for targeting mitochondrial biogenesis to overcome acquired resistance.

- Suppression of PGC1β-dependent mitochondrial biogenesis in EGFR-mutant NSCLC cells mediates the therapeutic response to osimertinib.
- Osimertinib-induced downregulation of PGC1β is mediated by suppression of FOSL1/AP-1-dependent transcription of PPARGC1B.
- Rebound elevation of PGC1β in osimertinib-resistant cells and tumors is associated with acquired resistance to osimertinib.
- Co-targeting mitochondrial biogenesis sensitizes osimertinib-resistant EGFR-mutant NSCLC to osimertinib.

## Abstract (web summary)

Acquired resistance to osimertinib remains inevitable in EGFR-mutant NSCLC. PGC1β-dependent mitochondrial biogenesis emerges as a key determinant of therapeutic response, providing a rationale for targeting mitochondrial biogenesis to overcome acquired resistance.

## Graphical abstract

**Figure:**
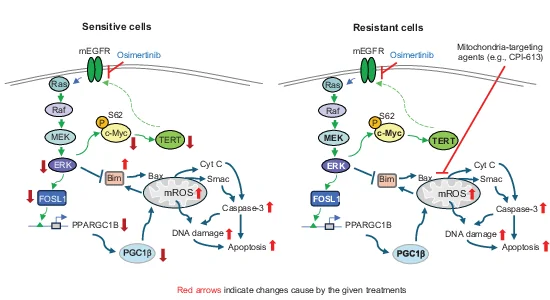

**Figure:**
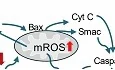

The paper explained **Problem**

EGFR-mutant non-small cell lung cancer is commonly treated with EGFR tyrosine kinase inhibitors, such as osimertinib. Although these agents have substantially improved patient outcomes, acquired resistance inevitably emerges in most patients, limiting their long-term clinical benefit.

**Results**

This study identifies PGC1β-dependent mitochondrial biogenesis as a key determinant of the response to osimertinib. Osimertinib suppresses PPARGC1B/PGC1β expression by inhibiting FOSL1/AP-1-mediated transcription, thereby reducing mitochondrial biogenesis in EGFR-mutant lung cancer cells. In models of acquired resistance, however, PGC1β expression is no longer effectively suppressed by osimertinib. Genetic reduction of PPARGC1B resensitizes resistant cells to osimertinib, whereas PPARGC1B overexpression promotes resistance. Combining osimertinib with the mitochondria-targeting agent CPI-613 suppresses the growth of resistant cells and tumors by enhancing mitochondrial stress and apoptosis.

**Impact**

These findings identify mitochondrial adaptation as a mechanism contributing to acquired resistance to osimertinib and provide a rationale for combining osimertinib with mitochondria-targeting agents, particularly in relapsed EGFR-mutant lung cancers with elevated PGC1β expression.

## Introduction

Non-small cell lung cancer (NSCLC) constitutes over 85% of all cases of lung cancer, a leading cause of cancer death among both men and women, and has a low 5-year survival rate of just over 25% in the US (Siegel et al, 2025) despite efforts made worldwide over the past decades. Targeted therapy specifically against lung cancer with epidermal growth factor receptor (EGFR) activating mutations, which occur at a rate of ~15% and ~50% in Western and Asian NSCLC populations, respectively, using EGFR tyrosine kinase inhibitors (EGFR-TKIs) has contributed greatly to the improved patient survival (Maione et al, 2025; Romaniello et al, 2025). In fighting the inevitable issue of acquired resistance, EGFR-TKIs have rapidly evolved from the initial first generation (e.g., erlotinib and gefitinib) to second generation (e.g., afatinib) and current third generation (e.g., osimertinib; also named AZD9291 or TAGRISSO^TM^) agents (Gomez-Randulfe et al, 2025). Compared to earlier generations, third-generation EGFR-TKIs have the advantages of selectively and irreversibly inhibiting EGFR with both common activating mutations and the T790M resistance mutation that accounts for approximately 60% of cases resistant to earlier generation EGFR-TKIs, while showing limited binding affinity to wild-type (WT) EGFR. Thus, third-generation agents are well-tolerated mutation-selective EGFR-TKIs, among which osimertinib is an FDA-approved drug for the treatment of patients with EGFR-mutant (EGFRm) NSCLC that has become resistant to earlier generation EGFR-TKIs due to T790M mutation as a second-line therapy and for advanced NSCLC with activating EGFR mutations as a first-line treatment either alone or in combination with platinum-based chemotherapy (Maione et al, 2025). Despite the proven and impressive clinical activity, patients eventually relapse due to the development of acquired resistance to treatment (Du et al, 2025; Romaniello et al, 2025). As such, long-term remission is infrequent, limiting the durable benefit of these agents.

Acquisition of a novel resistance mutation, C797S, is the most common target- or EGFR-dependent resistance mechanism, particularly when osimertinib is used as a second-line therapy. Moreover, there are other heterogeneous EGFR-independent resistance mechanisms such as *MET* amplification, acquired mutations in oncogenes (e.g., *BRAF*), and cell histological transformation (e.g., small cell lung cancer transformation) (Du et al, 2025; Romaniello et al, 2025). However, resistance mechanisms in most cases are largely unknown, particularly when osimertinib is used as a first-line therapy. Hence, efforts are urgently needed to thoroughly understand the mechanisms of acquired resistance. Progress in this regard will guide us to develop effective mechanism-driven strategies to manage acquired resistance to third-generation EGFR-TKIs.

PGC1β, a protein encoded by the *PPARGC1B* or *PGC1B* gene, shares extensive sequence identity with PGC1α and thus belongs to the PGC1 family that is known to coordinate the activity of certain transcription factors such as PPARγ and SREBP1 to modulate energy metabolism, lipid metabolism, mitochondrial biogenesis, and other cellular processes (Lin et al, 2005; Liu and Lin, 2011; Qian et al, 2024; Villena, 2015), some of which, such as SREBP1 and lipid metabolism, are tightly associated with therapeutic efficacy of osimertinib (Chen et al, 2021). Like PGC1α, PGC1β is also involved in certain types of cancer, such as colon cancer (Fisher et al, 2015), breast cancer (Chen et al, 2019; Deblois et al, 2010), intestinal cancer (Bellafante et al, 2014), and multiple myeloma (Zhang et al, 2018) by promoting tumorigenesis. Moreover, PGC-1β was found to be significantly upregulated in cisplatin-resistant cells and thus has been linked to acquired cisplatin chemoresistance (Yao et al, 2013). However, the potential connection between PGC1β modulation and the therapeutic efficacy of a targeted cancer therapy, such as EGFR-targeted therapy, has not been studied.

In our effort to understand the biology and action mechanisms of osimertinib in sensitive EGFRm NSCLC cells, we found that osimertinib-treated human EGFRm NSCLC cell lines possessed substantially reduced expression of *PPARGC1B* based on our RNA-seq analysis in EGFRm NSCLC cells exposed to DMSO vs. osimertinib, whereas *PPARGC1A* expression in the same cell lines was undetected and not altered by osimertinib. Given that PGC1β is critical for maintaining mitochondrial function and biogenesis (Qian et al, 2024; Villena, 2015), which plays critical roles in tumorigenesis and cancer progression (Dong et al, 2020), and targeting mitochondria as a potential cancer therapeutic target (Dong et al, 2020), we thus focused on studying the critical role of PGC1β inhibition including subsequent suppression of mitochondrial biogenesis or metabolism in mediating the therapeutic efficacy of osimertinib in EGFRm NSCLC cells/tumors and on developing therapeutic strategies to enhance osimertinib’s therapeutic efficacy via targeting PGC1β, particularly mitochondria. Through this study, we have demonstrated that the inhibition of PGC1β/mitochondria biogenesis is a critical mechanism accounting for the therapeutic efficacy of osimertinib, and co-targeting this mechanism effectively overcomes the challenging issue of acquired resistance to osimertinib and other third-generation EGFR-TKIs.

## Results

### Osimertinib downregulates *PPARGC1B* gene expression with inhibition of mitochondrial biogenesis in EGFRm NSCLC cells

**Figure 1:**
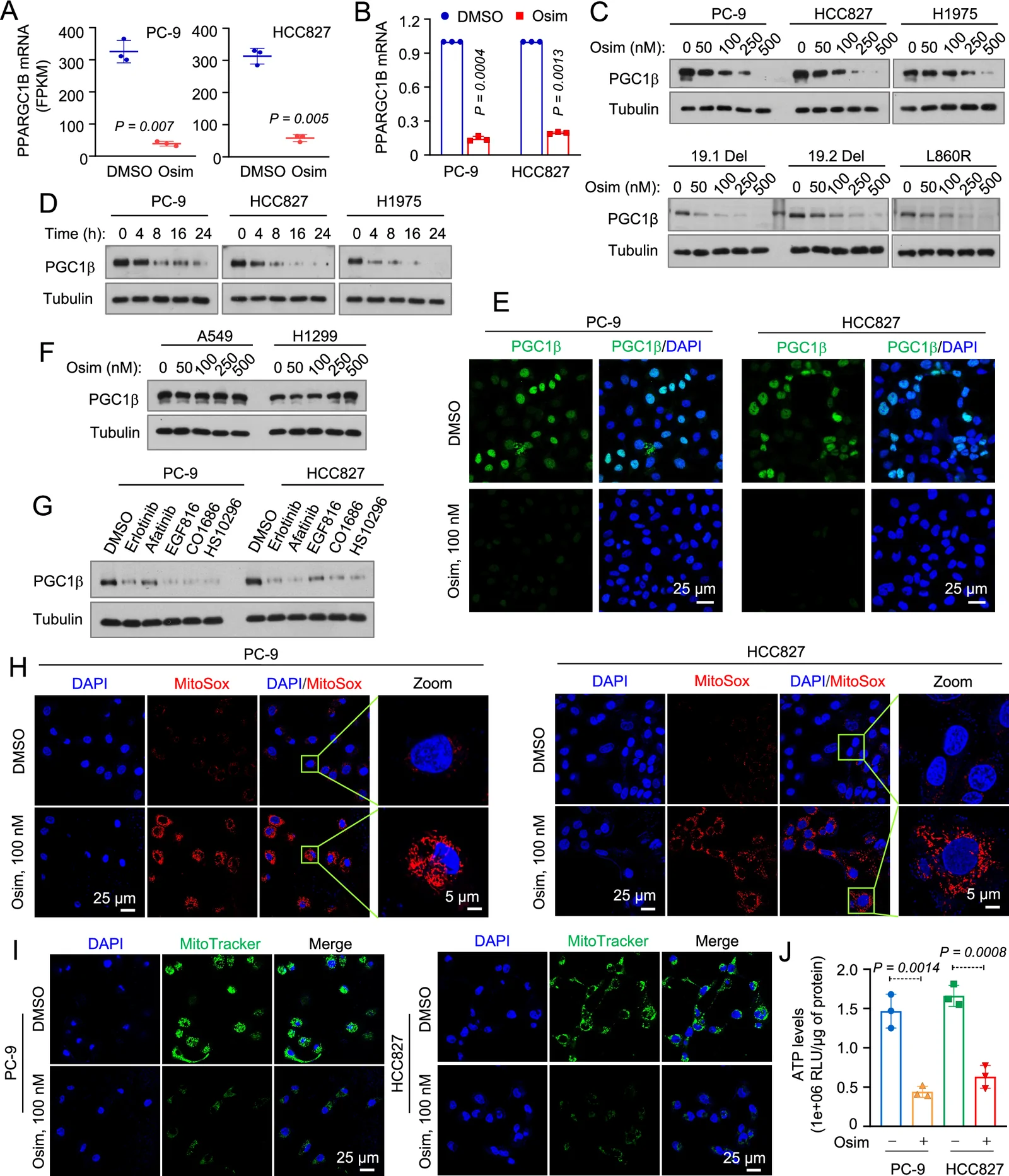
Osimertinib downregulates *PPARGC1B* gene expression with inhibition of mitochondrial biogenesis in EGFRm NSCLC cells. (**A**) RNA-seq analysis of PC-9 and HCC827 cells treated with DMSO or 100 nM osimertinib (Osim) for 14 h. (**B**) RT-qPCR validation of *PPARGC1B* suppression in the indicated cell lines following 14 h exposure to DMSO or 100 nM osimertinib. In each independent experiment, *PPARGC1B* mRNA expression was normalized to the corresponding DMSO control, which was set to 1. *P* values were calculated using a two-sided unpaired Student’s *t* test based on ΔCt values. (**C**–**G**) Western blot analysis of the indicated cell lines treated with osimertinib at various concentrations for 24 h (**C**, **F**), with 200 nM osimertinib for the indicated times (**D**), or with 200 nM of the indicated EGFR-TKIs for 24 h (**G**). (**E**) Immunofluorescent detection of PGC1β in PC-9 and HCC827 cells treated with DMSO or 100 nM osimertinib for 24 h. (**H**–**J**) The indicated cells were treated with DMSO or 100 nM osimertinib for 24 h and analyzed for mitochondrial ROS by MitoSOX™ Red staining (**H**), mitochondrial mass by MitoTracker® Green FM staining (**I**), and ATP levels by the Mitochondrial ToxGlo™ Assay (**J**). Data are presented as means ± SDs from three independent treatments. Statistical differences between the two given groups were conducted with a two-sided unpaired Student’s *t* test. Source data are available online for this figure.

Analysis of our previous RNA-seq data generated in EGFRm NSCLC cell lines treated with osimertinib revealed that *PPARGC1B* expression was significantly decreased by osimertinib in both PC-9 and HCC827 cell lines (Fig. 1A). This downregulation was confirmed by RT-qPCR (Fig. 1B). The levels of PGC1β protein were decreased in a concentration-dependent manner by osimertinib at concentrations ranging from 50 to 500 nM in different EGFRm NSCLC cell lines (Fig. 1C). The reduction of PGC1β occurred early at 4 h post osimertinib treatment and remained up to 24 h, the longest time tested (Fig. 1D). The reduction of nuclear PGC1β staining in osimertinib-treated cells was also clearly visualized with IF (Fig. 1E). As expected, osimertinib at the same conditions did not decrease PGC1β levels in A549 and H1299 cells possessing wild-type EGFR considering its mutation-selective function (Fig. 1F). In addition, other EGFR-TKIs including erlotinib (first generation), afatinib (second generation), EGFR816, CO1686 and HS10296 (third generation) also decreased the levels of PGC1β in both PC-9 and HCC827 cell lines (Fig. 1G). These results together convincingly demonstrate that osimertinib and other EGFR-TKIs inhibit the expression of *PPARGC1B*.

**Figure EV1:**
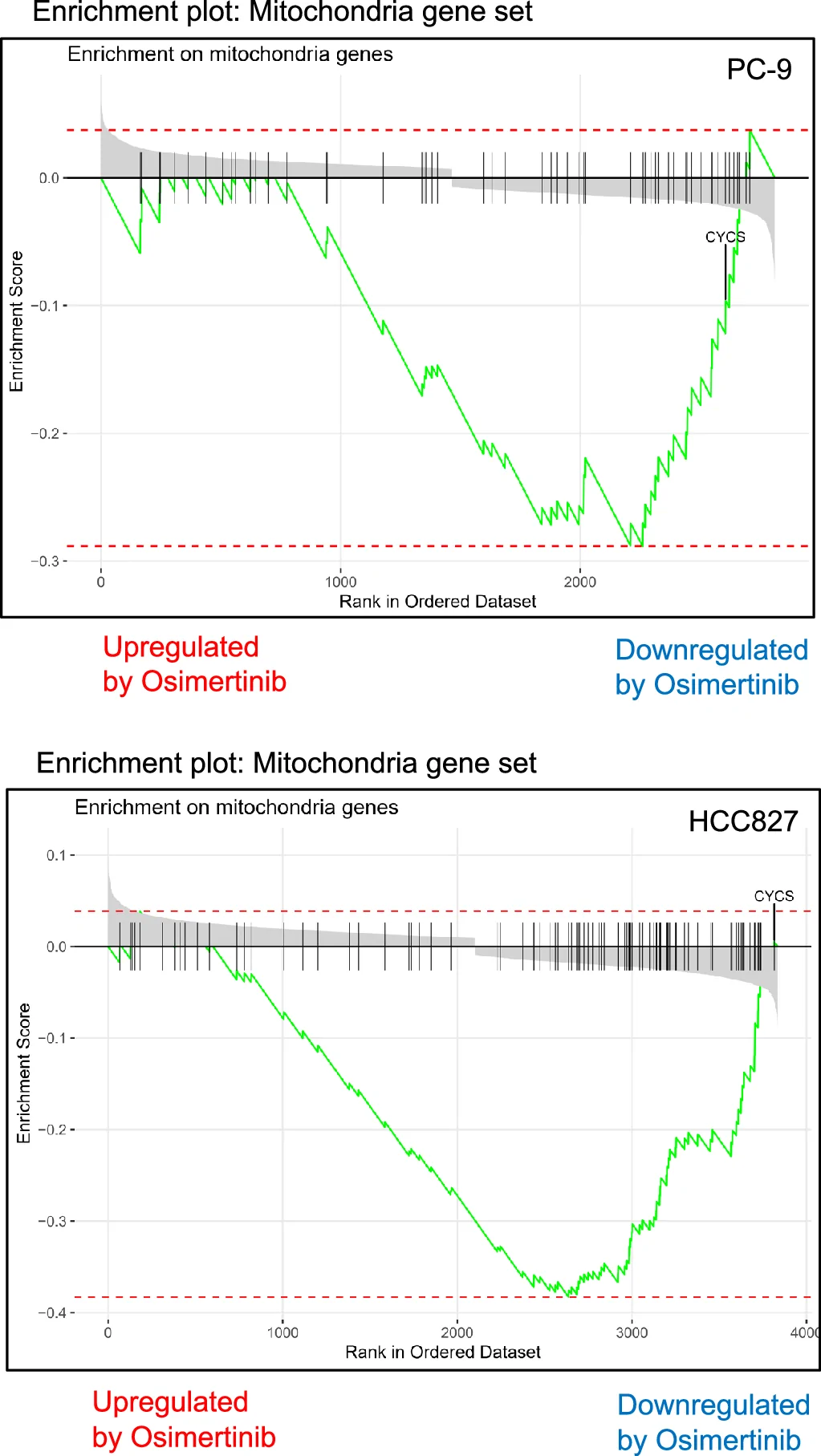
GSEAs showing negative enrichment of mitochondrial gene set in EGFRm NSCLC cells treated with osimertinib compared with DMSO control. Enrichment plot showing the running enrichment score (ES) for the mitochondrial gene set (Human MitoCarta3.0) across a ranked list of genes in HCC827 and PC9 cells treated with osimertinib versus DMSO. The *x* axis represents gene rank in the ordered dataset, and the *y* axis shows the running enrichment score. Vertical ticks indicate positions of mitochondrial genes. The negative ES indicates enrichment of mitochondrial genes toward the bottom of the ranked list, consistent with downregulation upon treatment.

One of the key biological functions of PGC1β is to positively regulate mitochondrial biogenesis (Liu and Lin, 2011; Qian et al, 2024; Villena, 2015). Gene enrichment analysis also showed that the expression of many mitochondria-related genes was downregulated by osimenrtinib in both PC-9 and HCC827 cells (Fig. EV1). Thus, we explored whether osimertinib accordingly inhibits mitochondrial biogenesis by measuring mitochondrial ROS generation (MitoSox staining), ATP production, and mitochondrial mass (MitoTracker green staining) in EGFRm NSCLC cells exposed to osimertinib. We found that MitoSox staining intensity in osimertinib-treated cells was increased (Fig. 1H), whereas MitoTracker green staining was greatly diminished in these cells (Fig. 1I) compared with their corresponding control cells. These results indicate that osimertinib increases mitochondrial ROS generation while suppressing mitochondrial mass. Moreover, ATP amounts were significantly reduced in cells exposed to osimertinib compared with control cells (Fig. 1J). Collectively, these findings suggest that osimertinib inhibits mitochondrial biogenesis in EGFRm NSCLC cells.

### Osimertinib inhibits *PPARGC1B* expression via suppression of FOSL1/AP-1 in EGFRm NSCLC cells

**Figure 2:**
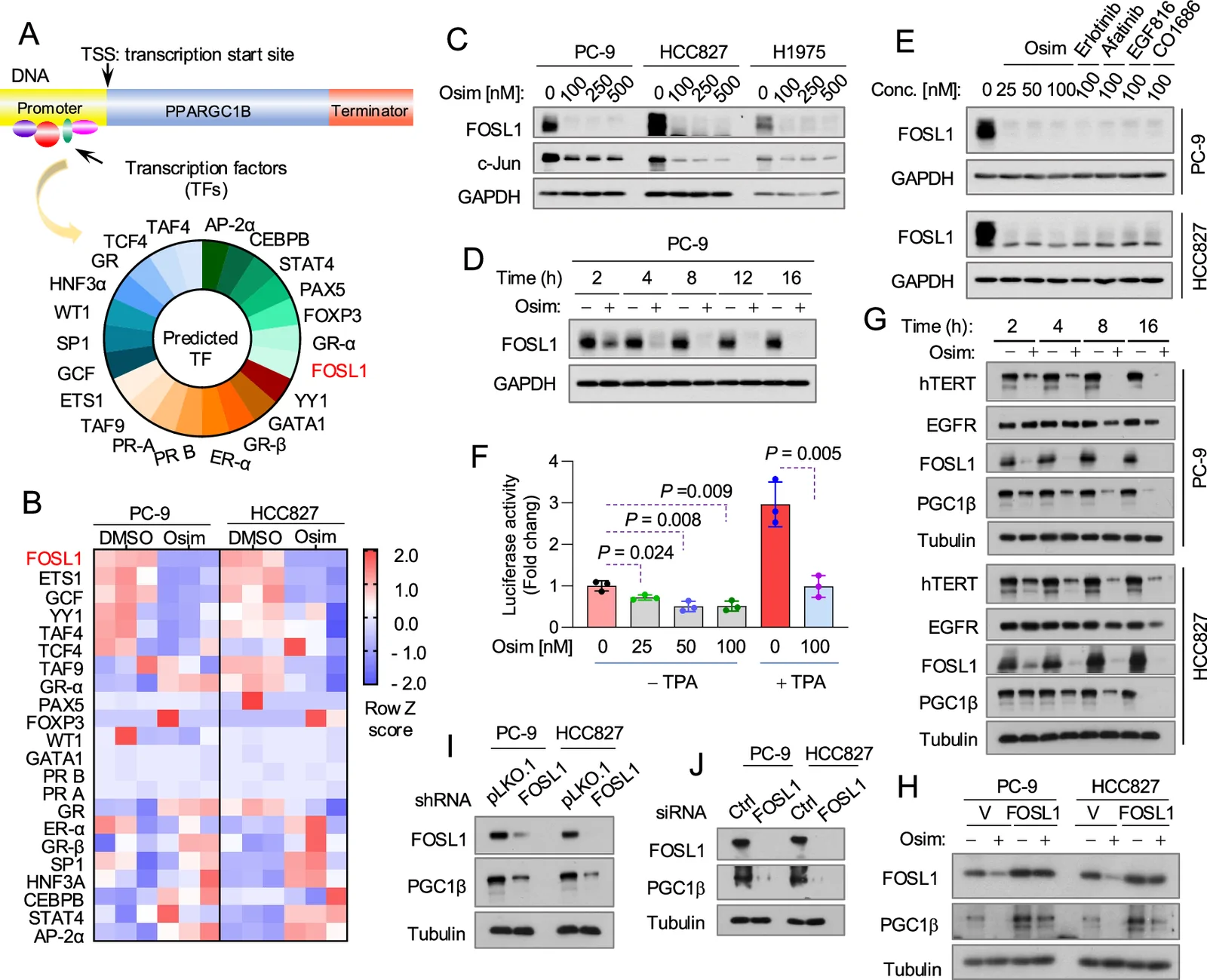
Osimertinib inhibits *PPARGC1B* expression via suppression of FOSL1/AP-1 in EGFRm NSCLC cells. (**A**, **B**) Putative transcription factors were predicted using the PROMO database (**A**), and their alterations were visualized as a heatmap based on RNA-seq data from PC-9 and HCC827 cells treated with DMSO or 100 nM osimertinib (Osim) for 14 h (**B**). (**C**–**E**) The indicated cell lines were treated with various concentrations of osimertinib for 14 h (**C**, **E**) or 200 nM osimertinib for the indicated times (**D**). Proteins of interest were detected by western blotting. (**F**) PC-9 cells transfected with pAP1-luc plasmid overnight were treated with the indicated concentrations of osimertinib in the absence and presence of 100 ng/ml TPA for 6 h. Data are presented as mean ± SD (*n* = 3). Statistical differences between two given groups were analyzed using a two-sided unpaired Student’s *t* test. (**G**) The tested cell lines were treated with 200 nM osimertinib for the indicated times. Proteins of interest were detected by Western blotting. (**H**–**J**) The indicated cell lines expressing empty vector (V) and FOSL1, respectively, were treated with DMSO or 200 nM osimertinib for 16 h (**H**), were infected with lentiviruses carrying FOSL1 shRNA (**I**), or were transfected with the given siRNA for 48 h (**J**). Proteins of interest were detected with Western blotting. Source data are available online for this figure.

To understand the mechanism by which osimertinib inhibits *PPARGC1B* expression, we analyzed the *PPARGC1B* promoter region for predicted transcriptional factors that may regulate *PPARGC1B* expression (Fig. 2A). By looking at their alterations in osimertinib-treated cells based on our RNA-seq analysis, we found that FOSL1 (Fra-1) expression was the most significantly decreased (Fig. 2B). At the protein level, osimertinib at concentrations ranging from 100 to 500 nM effectively decreased FOSL1 levels in different EGFRm NSCLC cell lines (Fig. 2C). This reduction occurred early at 2 h after osimertinib treatment (Fig. 2D). Beyond osimertinib, other EGFR-TKIs also decreased FOSL1 levels (Fig. 2E). Given that FOSL1 is a component of the AP-1 dimer that is responsible for the regulation of gene transcription (Talotta et al, 2020; Zeng et al, 2022) and osimertinib decreased the levels of c-Jun, another key component of AP-1, as demonstrated in our previous study (Zhang et al, 2021) and current study (Fig. 2C), we further examined the effect of osimertinib on AP-1 activity. As presented in Fig. 2F, osimertinib not only decreased basal levels of AP-1 activity but also blocked AP-1 activity increased by TPA, a known AP-1 activator (Lamph et al, 1988), suggesting that osimertinib inhibits AP-1 activity.

To determine the connection between FOSL1 and PGC1β, we compared kinetic changes of FOSL1 and PGC1β in EGFRm NSCLC cells exposed to osimertinib and noted that FOSL1 reduction occurred at 2 h post treatment; this was ahead of a strong PGC1β decrease that happened at 4 h after treatment (Fig. 2G). Moreover, the expression of ectopic FOSL1 in both PC-9 and HCC827 elevated PGC1β levels and rescued PGC1β reduction caused by osimertinib (Fig. 2H), whereas FOSL1 knockdown with both siRNA and shRNA accordingly decreased PGC1β levels in these cell lines (Fig. 2I,J). Hence, there is clearly a causal relationship between FOSL1 and PGC1β expression.

**Figure 3:**
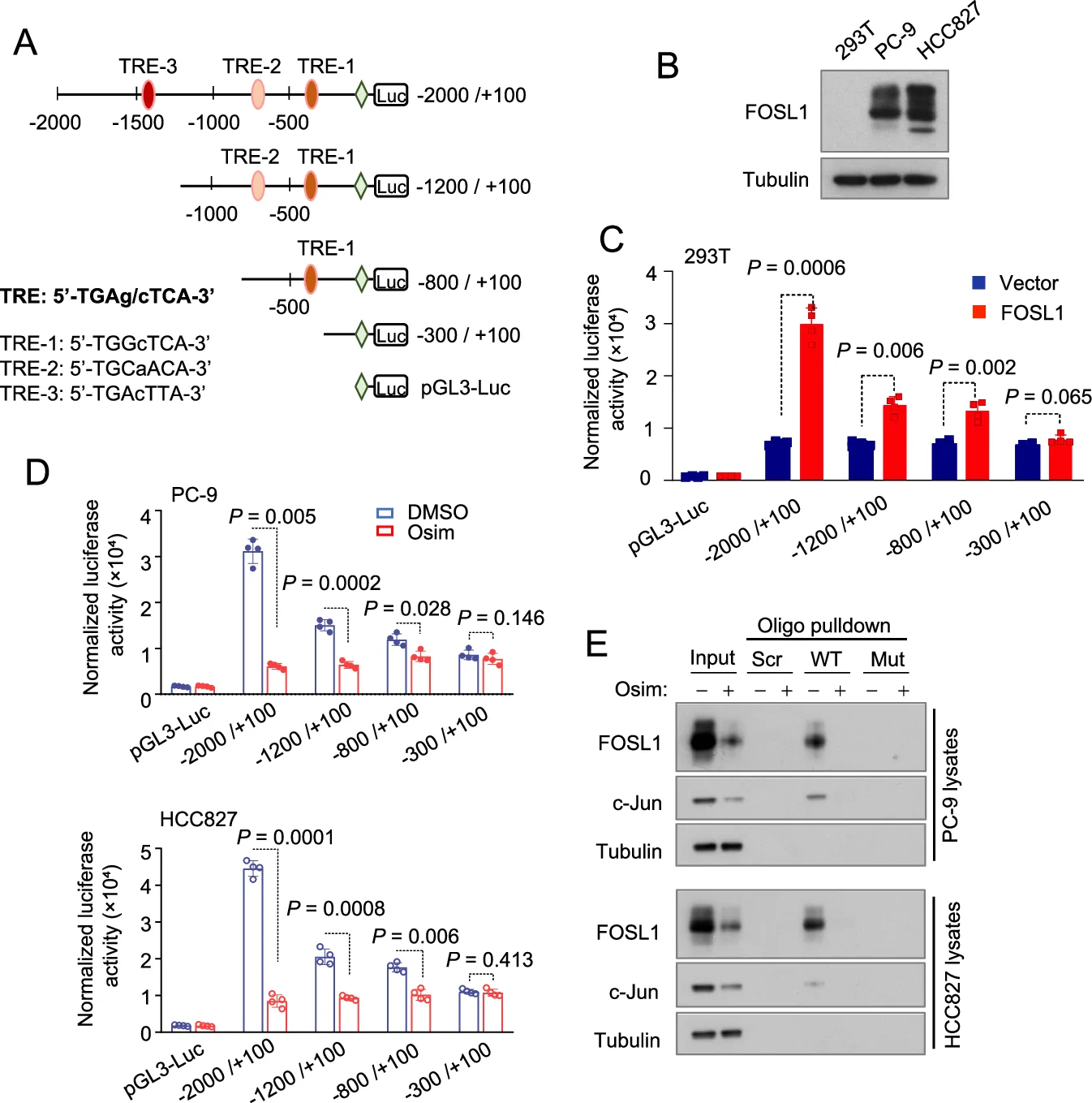
Osimertinib inhibits FOSL1/AP-1-dependent *PPARGC1B* expression via directly suppressing AP-1-mediated gene transactivation in EGFRm NSCLC cells. (**A**) Schematic representation of reporter constructs containing the *PPARGC1B* promoter region with putative TPA-response elements (TRE) and its deletion mutants. (**B**), Detection of FOSL1 by western blotting in 293T cells in comparison with PC-9 and HCC827 cells. (**C**) 293T cells were co-transfected with the *FOSL1* expression plasmid plus the indicated reporter constructs, respectively, for 48 h and then harvested for luciferase assay. The results are means ± SDs of four replicate determinations. (**D**) The indicated cell lines were transfected with the given reporter constructs for 24 h, followed by treatment with 200 nM osimertinib for an additional 16 h. Cells were then harvested for assaying luciferase activity. Data are presented as the means ± SDs of four independent determinations. (**E**) The indicated biotin-oligos were incubated with whole-cell protein lysates prepared from the PC-9 and HCC827 cells exposed to DMSO or 200 nM osimertinib for 16 h. Pulldown assays were performed using streptavidin–agarose beads. Bound FOSL1 and c-Jun were detected by Western blotting. Statistical differences between two given groups were conducted with a two-sided unpaired Student’s *t* test. Source data are available online for this figure.

Analysis of the *PPARGC1B* gene promoter region identified three putative AP-1 binding sites or TPA-response elements (TREs; Fig. 3A). To characterize the function of these TREs, we co-transfected *FOSL1* together with different reporter constructs with and without the TREs, respectively, in 293 T cells that expressed very low levels of endogenous FOSL1 (Fig. 3B) and found that *FOSL1* transfection significantly increased luciferase activity of the reporter constructs with 1, 2, or 3 TREs, but not that of a construct without a TRE, i.e., pGL3 (−300/ + 100)-luc. However, the highest luciferase activity was detected in cells co-transfected with *FOSL1* and pGL3 (−2000/ + 100)-luc (Fig. 3C). These results suggest that these TREs are functional, and TRE-3 may be the predominant one. Following this experiment, we transfected PC-9 and HCC827 cells with these reporter constructs, followed by treatment with osimertinib to determine the impact of osimertinib on the transcriptional activity of these reporter constructs. As presented in Fig. 3D, cells transfected with reporter constructs containing the full-length *PPARGC1B* 5’-flanking region (−2000/ + 100) exhibited the highest levels of luciferase activity in comparison with cells transfected with reporter constructs harboring a truncated 5’-flanking region lacking the one distal AP-1 binding site (TRE-3; −1200/ + 100), the two distal AP-1 binding sites (TRE-2 and TRE-3; −800/ + 100), or the three AP-1 binding sites (TRE-1, TRE2 and TRE-3; −300/ + 100), which had relatively low basal levels of luciferase activity (Fig. 3A,D). The increased luciferase activity in cells transfected with pGL3 (-2000/ + 100)-Luc, pGL3 (-1200/ + 100)-Luc, or pGL3 (−800/ + 100)-Luc was significantly inhibited when exposed to osimertinib. By further conducting oligo pull-down assays, we found that an oligo carrying TRE-3, but not scrambled or TRE-3-mutant oligos, could pull down FOSL1 and c-Jun proteins in both PC-9 and HCC827 cell lines; the bindings of these proteins were drastically reduced in cells treated with osimertinib (Fig. 3E). These findings suggest that FOSL1 indeed binds to TRE-3 in the *PPARGC1B* 5’-flanking region. These results together demonstrate that these AP-1 binding sites, particularly TRE-3, located in the *PPARGC1B* 5’-flanking region, mediate AP-1-dependent transcription of the *PPARGC1B* gene and that osimertinib suppresses *PPARGC1B* gene expression through an AP-1-mediated mechanism.

**Figure EV2:**
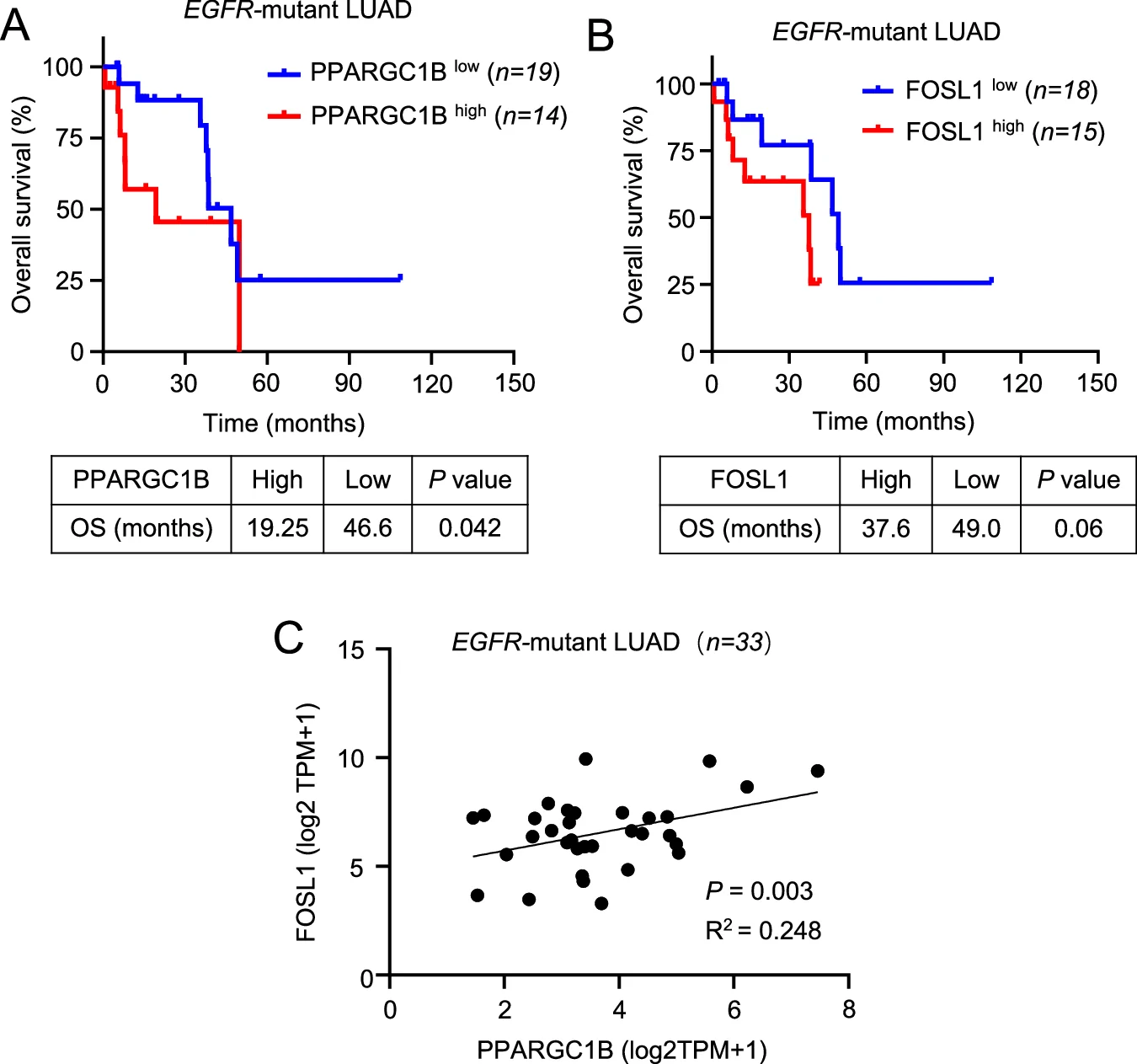
TCGA analysis of *PPARGC1B* and *FOSL1* expression in EGFRm lung adenocarcinoma (LUAD) tissues and their impacts on patient survival. (**A**, **B**) High versus low expression of PPARGC1B or FOSL1 in 33 tumors was stratified using the median expression level as the cutoff. Overall survival was analyzed by Kaplan–Meier survival analysis, and P values were determined by the log-rank test. (**C**) PPARGC1B and FOSL1 mRNA expression data from the 33 EGFRm tumors in the TCGA database were extracted, and their correlation was then analyzed. TPM, transcripts per million.

We finally analyzed the TCGA database and found that high expression of *PPARGC1B* or *FOSL1* in EGFRm NSCLC tissues was associated with a trend of worse overall survival (*P* = 0.042 and 0.06, respectively) (Fig. EV2A,B). Moreover, the correlation between the expression of these two genes was significant (*P* = 0.003) (Fig. EV2C).

### Osimertinib-induced hTERT inhibition contributes to suppression of *PPARGC1B* gene expression

**Figure 4:**
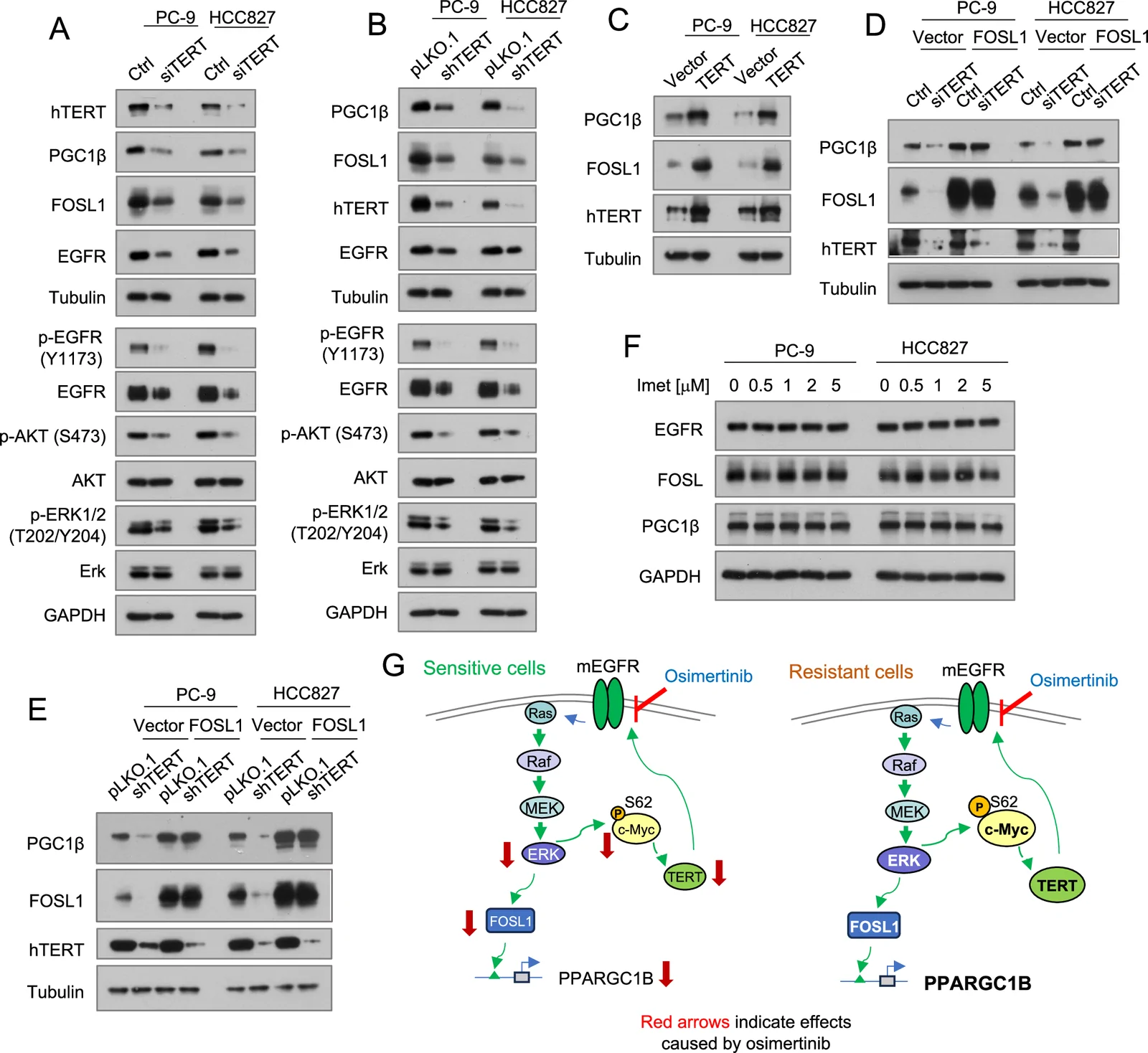
hTERT inhibition contributes to suppression of *PPARGC1B* gene expression by osimertinib in EGFRm NSCLC cells via a telomerase-independent manner. (**A**) PC-9 and HCC827 cells were transfected with siRNA for 48 h. (**B**) PC-9 and HCC827 cells were infected with pLKO.1 control or hTERT shRNA lentiviruses. (**C**) PC-9 and HCC827 cells were transfected with either an empty vector (V) or an hTERT expression construct. (**D**, **E**) PC-9 and HCC827 cells transfected with vector or FOSL1 plasmids were further transfected with control or hTERT siRNA (**D**) or infected with pLKO.1 or hTERT shRNA lentiviruses (**E**) for 48 h. (**F**) The indicated cell lines were exposed to the varied concentrations of imetelstat for 48 h. Proteins of interest, as indicated in the aforementioned studies, were detected with western blotting. (**G**) Schematic working models for the contribution of hTERT inhibition to suppression of *PPARGC1B* gene expression by osimertinib in sensitive EGFRm NSCLC cells (left panel) and for rebound elevation of *PPARGC1B* expression in osimertinib-resistant cells (right panel). Suppression of ERK-dependent c-Myc stability and c-Myc-dependent hTERT expression by osimertinib including rebound elevation of c-Myc and hTERT in osimertinib-resistant cells are previous findings as reported (Chen et al, 2024; Zhu et al, 2021). Source data are available online for this figure.

hTERT/telomere dysfunction was suggested to be involved in the suppression of *PPARGC1B* expression and subsequent mitochondrial metabolism via activation of p53 (Sahin et al, 2011). We previously demonstrated that osimertinib decreases c-Myc levels via induction of ERK inhibition-caused c-Myc degradation (Zhu et al, 2021); this further leads to inhibition of *hTERT* expression and subsequent induction of telomere dysfunction and telomeric DNA damage in EGFRm NSCLC cells (Chen et al, 2024). Although the tested PC-9 and HCC827 cell lines possess mutant p53, we were still interested in determining whether there is a connection between hTERT suppression and PGC1β inhibition in EGFRm NSCLC cells. In both PC-9 and HCC827 cell lines, hTERT reduction was accompanied by PGC1β downregulation (Fig. 2G). Hence, we first determined whether modulation of hTERT levels alters the levels of PGC1β in EGFRm NSCLC cells. Genetic knockdown of hTERT expression using both siRNA and shRNA decreased PGC1β levels in both PC-9 and HCC827 cell lines (Fig. 4A,B), whereas enforced expression of ectopic hTERT increased the levels of PGC1β in these cell lines (Fig. 4C). These results suggest that there is indeed a connection between hTERT and the regulation of PGC1β expression. To explore how hTERT positively regulates PGC1β expression in cell lines with mutant p53, we further looked at FOSL1 alteration in the above cell lines in which hTERT expression was knocked down or overexpressed and found that FOSL1 levels were reduced in cell lines in which hTERT expression was knocked down (Fig. 4A,B) and increased in the cell lines expressing ectopic hTERT (Fig. 4C). These findings indicate that hTERT positively regulates the levels of both FOSL1 and PGC1β in EGFRm NSCLC cells. To determine whether hTERT regulates PGC1β expression through FOSL1, we conducted hTERT knockdown in PC-9 and HCC827 cell lines expressing ectopic FOSL1 to see whether enforced overexpression of *FOSL1* rescues the downregulation of PGC1β caused by hTERT knockdown. As presented in Fig. 4D, hTERT knockdown with siRNA decreased the levels of PGC1β in vector-transfected PC-9 and HCC827 cells, but not in cells expressing the ectopic *FOSL1* gene. Similar results were also generated with hTERT shRNA (Fig. 4E). These results suggest that hTERT functions through FOSL1 to positively regulate *PPARGC1B* expression. We further determined the effects of imetelstat, a known oligonucleotide telomerase inhibitor (Lennox et al, 2024), on the levels of EGFR, FOSL1, and PGC1β in both PC-9 and HCC827 cell lines and found that imetelstat did not decrease the levels of these proteins (Fig. 4F), suggesting that hTERT may regulate the EGFR, FOSL1, and PGC1β axis through a telomerase-independent mechanism. This may also explain why hTERT can regulate this axis in cells with mutant p53. Hence, it is likely that ERK inhibition-caused c-Myc degradation and subsequent suppression of hTERT expression by osimertinib may further enhance suppression of FOSL1-dependent *PPARGC1B* expression via EGFR downregulation in sensitive EGFRm NSCLC cells (Fig. 4G, left panel).

In bladder cancer, it was shown that hTERT knockdown decreases the expression of both FOSL1 and EGFR (Kraemer et al, 2006). Given that EGFR levels were reduced in both PC-9 and HCC827 cell lines exposed to osimertinib, albeit at a relatively late time (e.g., at 8 h; Fig. 2G), we further determined whether hTERT also regulates EGFR levels in EGFRm NSCLC cell lines. We found that EGFR levels were reduced in both PC-9 and HCC827 cells in which hTERT expression was knocked down. Accordingly, the levels of p-EGFR, p-AKT, and p-ERK were all reduced in these hTERT knockdown cell lines (Fig. 4A,B). These results indicate that hTERT knockdown causes EGFR reduction and subsequent downstream signaling suppression. Hence, EGFR downregulation caused by hTERT inhibition may also contribute to suppression of FOSL1-mediated *PPARGC1B* expression (Fig. 4G, left panel).

### Osimertinib-resistant EGFRm NSCLC cell lines and tumors have elevated levels of PGC1β that are associated with reduced cell response to osimertinib

**Figure 5:**
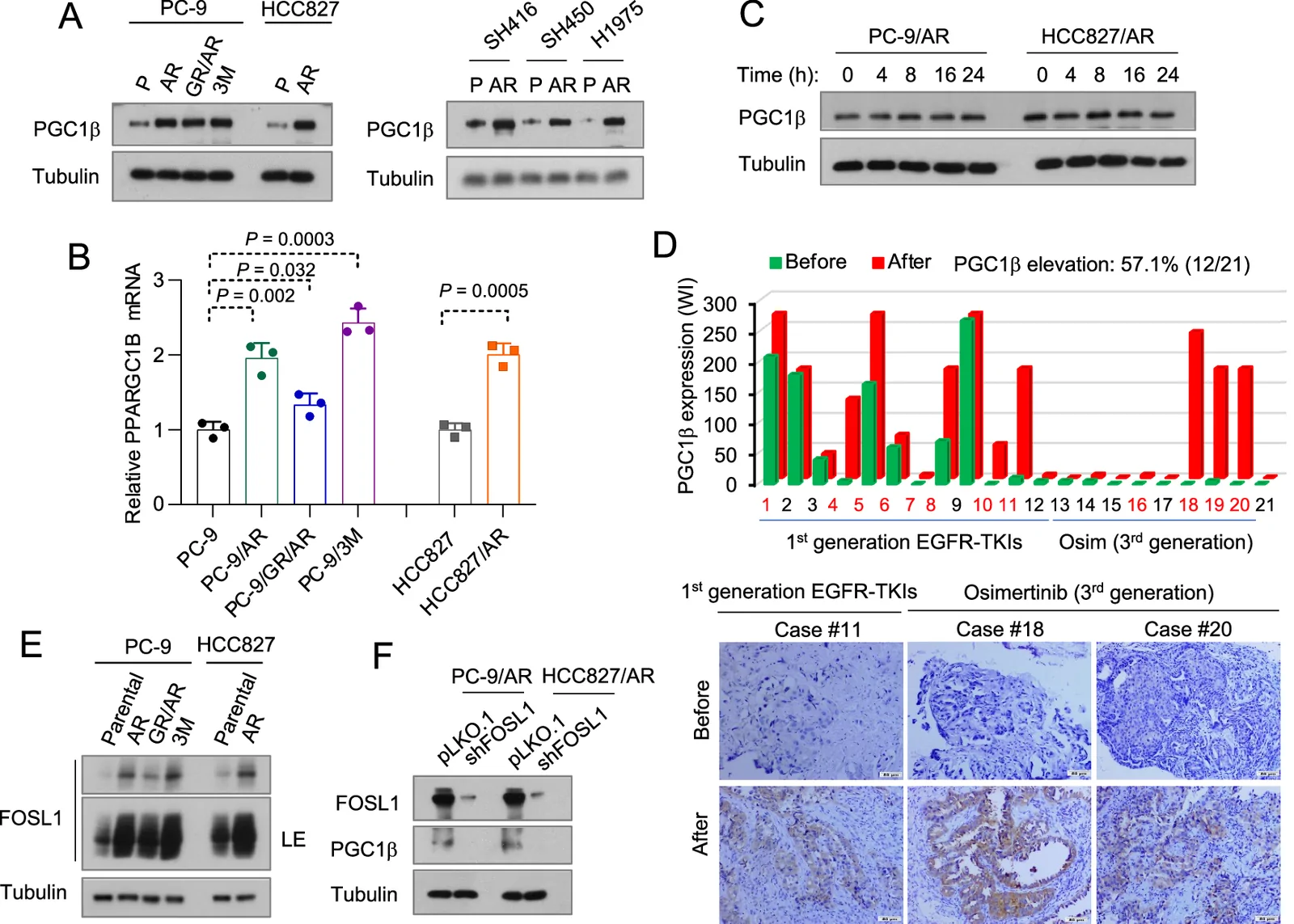
Osimertinib-resistant EGFRm NSCLC cell lines and tumors have elevated levels of PGC1β, likely due to elevated FOSL. (**A**) Western blot analysis of basal levels of PGC1β protein expression in the indicated cell lines. (**B**) Cellular total RNA was prepared from the indicated cell lines and then used for the detection of *PPARGC1B* mRNA using RT-qPCR. The results are means ± SDs of triplicated samples. Statistical differences between the two given groups were conducted with a two-sided unpaired Student’s *t* test. (**C**) Western blot analysis of PGC1β protein expression in the tested cell lines treated with or without 200 nM osimertinib for the indicated times. (**D**) IHC detection of PGC1β expression in human EGFRm NSCLC tissues before and after relapse from EGFR-TKI treatment. WI, weighted index, which represents % positive stain × intensity score (0–3). Red font on case number labels (*x* axis) indicates those with PGC1β elevation after treatment. (**E**) Western blot analysis of basal levels of FOSL1 protein in the indicated cell lines. (**F**) Western blot analysis of the indicated proteins in different EGFRm cell lines infected with lentiviruses carrying FOSL1 shRNA. Source data are available online for this figure.

To determine the impact of PGC1β alteration on the therapeutic outcomes of osimertinib, we compared basal levels of PGC1β between osimertinib resistant cell lines and their matched parental cell lines with varied resistance mechanisms to osimertinib including *MET* amplification and EGFR C797S mutation (Appendix Table S1) and detected clearly increased levels of PGC1β in the tested osimertinib-resistant cell lines compared with those in the corresponding parental cell lines (Fig. 5A). We further detected *PPARGC1B* mRNA expression in these cell lines and found that *PPARGC1B* mRNA levels were also significantly increased in osimertinib resistant cell lines compared with those in the corresponding parental cell lines (Fig. 5B), indicating increased *PPARGC1B* gene transcription. Osimertinib did not decrease PGC1β levels in the resistant cell lines (Fig.5C), indicating that osimertinib-resistant cell lines lose response to osimertinib modulation of PGC1β. In paired tissues obtained before EGFR-TKI treatment, including with osimertinib (baseline) and after relapse from treatment, we detected PGC1β elevation in post relapse tissues in 12 of 21 cases (57.1%) and no change in the remaining cases (Fig. 5D), thus confirming PGC1β elevation in patient NSCLC tissues with resistance to EGFR-TKIs. Some novel mutations were detected in some cases of after-relapse tissues, although no *EGFR C797S* mutation and *MET* amplification were identified (Appendix Table S2); however, no clear association between these mutations and PGC1β expression was observed.

We also compared the levels of FOSL1 between parental and corresponding osimertinib-resistant cell lines and found that FOSL1 levels were also elevated in osimertinib-resistant cell lines in comparison with their corresponding parental cell lines (Fig. 5E). Knockdown of *FOSL1* in the osimertinib-resistant cell lines resulted in a reduction of PGC1β (Fig. 5F), suggesting that PGC1β elevation in osimertinib-resistant cell lines is likely caused by increased FOSL1 expression. It is likely that the elevated c-Myc and hTERT in osimertinib-resistant cell lines as demonstrated previously (Chen et al, 2024; Zhu et al, 2021) will further facilitate EGFR/MEK/ERK-dependent FOSL1 expression, contributing to elevated *PPARGC1B* expression in osimertinib-resistant cells (Fig. 4G, right panel).

**Figure 6:**
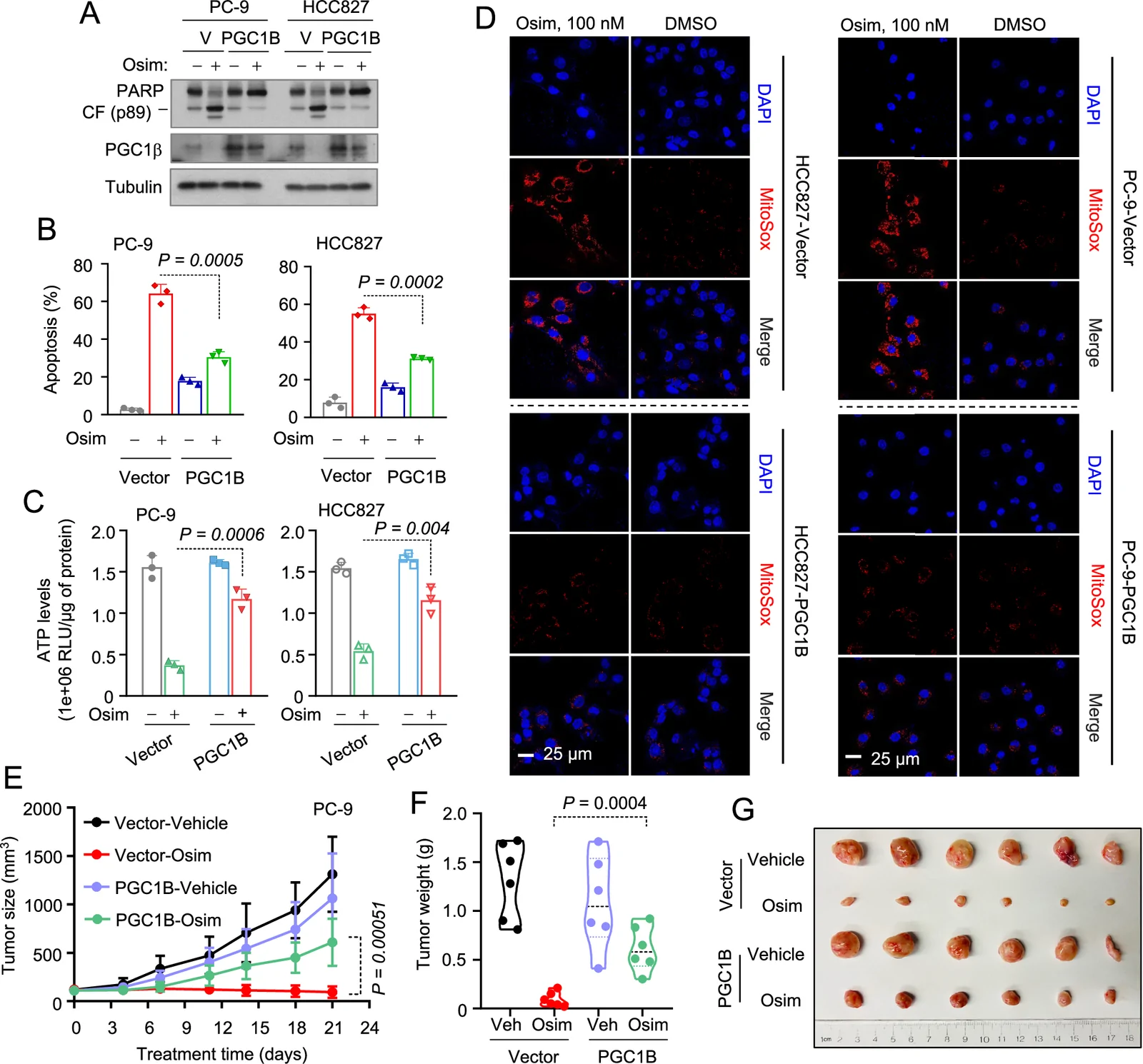
Elevated PGC1β in osimertinib-resistant EGFRm NSCLC cells is associated with reduced cell response to osimertinib. (**A**–**D**) The indicated cell lines expressing vector (V) or *PPARGC1B* gene were exposed to DMSO or 100 nM osimertinib (Osim) for 24 h (**A**, **D**, **C**) or 48 h (**B**). The proteins of interest were detected with western blotting (**A**). Apoptotic cells were analyzed with annexin V staining/flow cytometry (**B**). ATP levels were measured using the Mitochondrial ToxGlo™ Assay (**C**). Mitochondrial ROS were detected by MitoSOX™ Red staining (**D**). Data are presented as means ± SDs from three independent treatments (**B**, **C**). (**E**–**G**) Mice inoculated with the indicated tumors were treated with vehicle or osimertinib (5 mg/kg, o.g., daily). Tumor volumes were measured at the indicated times (**E**), and tumor weights were measured at the end of the treatments (**F**) and photographed (**G**). The data are means ± SEs (*n* = 6). Statistical differences between two given groups were conducted with a two-sided unpaired Student’s *t* test. Source data are available online for this figure.

**Figure EV3:**
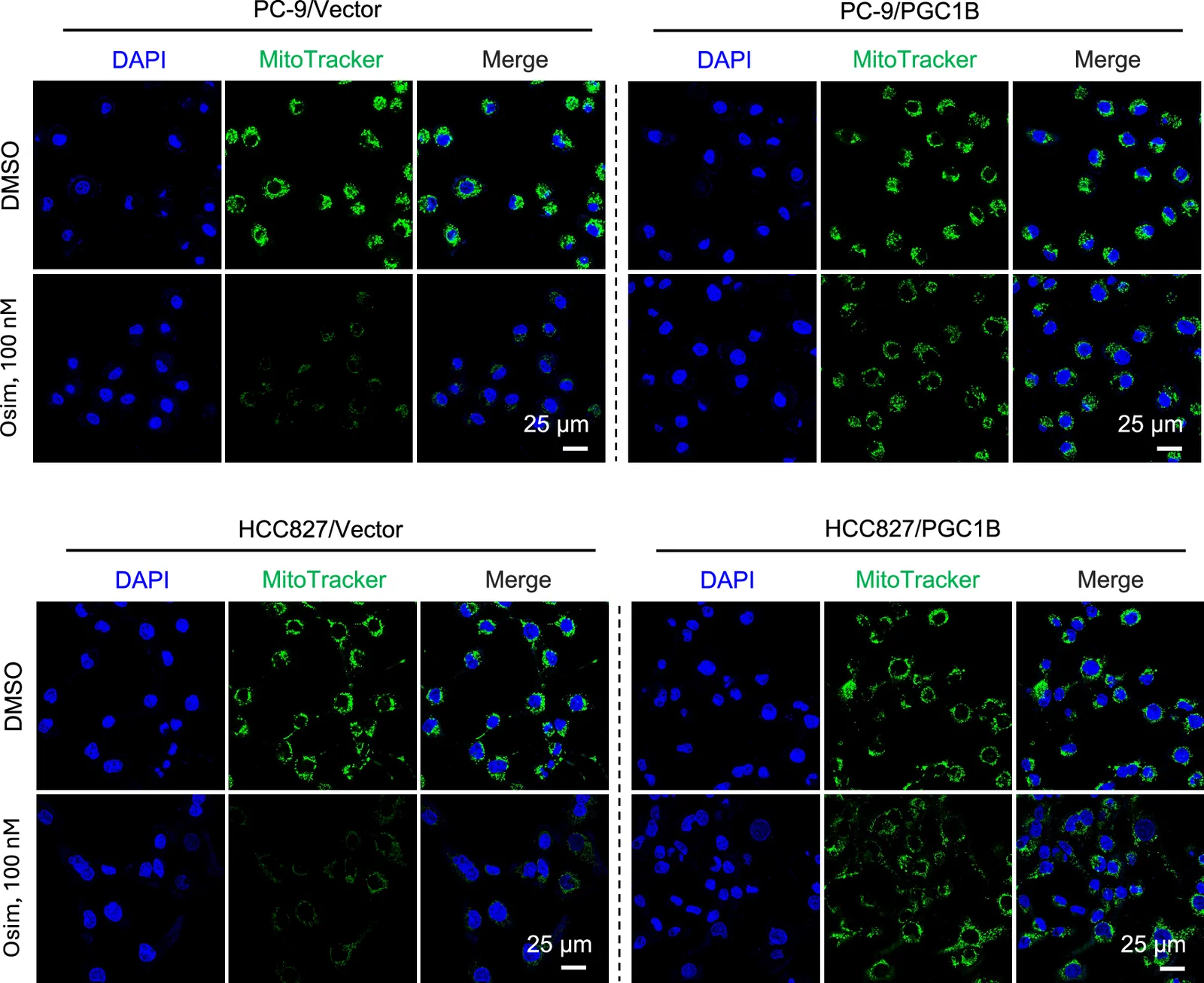
Impact of enforced expression of ectopic *PPARGC1B* (*PGC1B*) in EGFRm NSCLC cell lines on osimertinib-induced mitochondrial alteration. The indicated cell lines were treated with 100 nM osimertinib for 24 h and then stained with MitoTracker green.

To determine whether elevated PGC1β contributes to acquired resistance to osimertinib, we enforced the expression of ectopic *PPARGC1B* in the sensitive EGFRm NSCLC cell lines and then analyzed its impact on cell response to osimertinib. The expression of ectopic *PPARGC1B* rescued PGC1β reduction induced by osimertinib (Fig. 6A). Accordingly, osimertinib induced PARP cleavage and increased annexin V-positive cells in the vector control cells, but had minimal or no effect in *PPARGC1B*-expressing cells (Fig. 6A,B), indicating a protective effect of ectopic *PPARGC1B* expression on the induction of apoptosis by osimertinib. In agreement, osimertinib decreased ATP levels (Fig. 6C), increased mitochondrial ROS production (or MitoSox staining; Fig. 6D), and suppressed mitochondrial mass (or MitoTracker Green staining; Fig. EV3); however, these effects were reduced in the corresponding *PPARGC1B*-expressing cell lines. Hence, expression of ectopic *PPARGC1B* reduced the inhibition of mitochondrial biogenesis induced by osimertinib in EGFRm NSCLC cells. Beyond these findings, we also found that osimertinib had a significantly reduced effect on inhibiting the growth of PC-9/ PGC1β tumors in comparison to PC-9/vector control tumors as evaluated by measuring both tumor sizes and weights (Fig. 6E–G). Under the tested conditions, mouse body weights among different groups were comparable (Appendix Fig. S1). Hence, enforced expression of ectopic *PPARGC1B* clearly confers resistance of EGFRm tumors to osimertinib in vivo. These data collectively suggest that elevated PGC1β mediates resistance to EGFRm NSCLC cells to osimertinib.

### Enforced reduction of *PPARGC1B* in osimertinib-resistant cell lines via gene knockdown restores the sensitivity of cells with osimertinib acquired resistance to osimertinib

**Figure 7:**
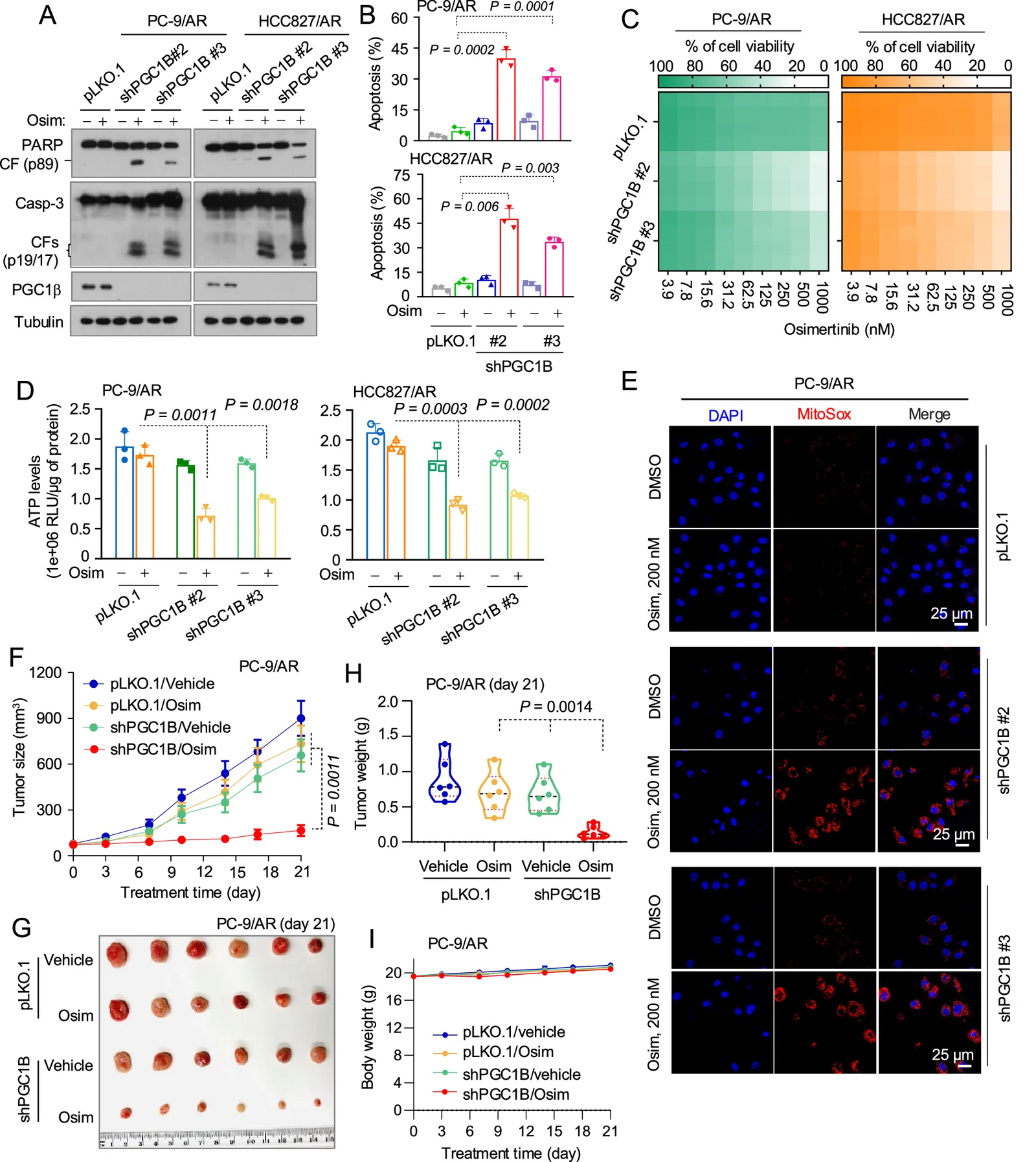
Enforced reduction of *PPARGC1B* in osimertinib-resistant cell lines via genetic gene knockdown restores cell sensitivities to osimertinib. (**A**–**E**) The indicated cell lines expressing pLKO.1 or shPPARGC1B were exposed to DMSO or 200 nM osimertinib for 48 h (**A**, **B**), 72 h (**C**), or 24 h (**D**, **E**). The proteins of interest were detected with western blotting (**A**). Annexin V-positive cells were analyzed with flow cytometry (**B**). Cell numbers were estimated with the SRB assay (**C**). ATP levels were measured using the Mitochondrial ToxGlo™ Assay (**D**). Mitochondrial ROS were detected by MitoSOX™ Red staining (**E**). The data are means ± SDs of triplicate (**B**, **D**) or four replicate (**C**) determinations. (**F**–**I**) Mice inoculated with the indicated tumors were treated with vehicle or osimertinib (5 mg/kg, o.g., daily). Tumor volumes and body weights were recorded at the indicated time points (**F**, **I**). At the end of the treatment, tumors were removed, photographed (**G**), and weighed (**H**). The data are means ± SEs (*n* = 6). Statistical differences between two given groups and among multiple groups were conducted with a two-sided unpaired Student’s *t* test and a one-way ANOVA test, respectively. Source data are available online for this figure.

**Figure EV4:**
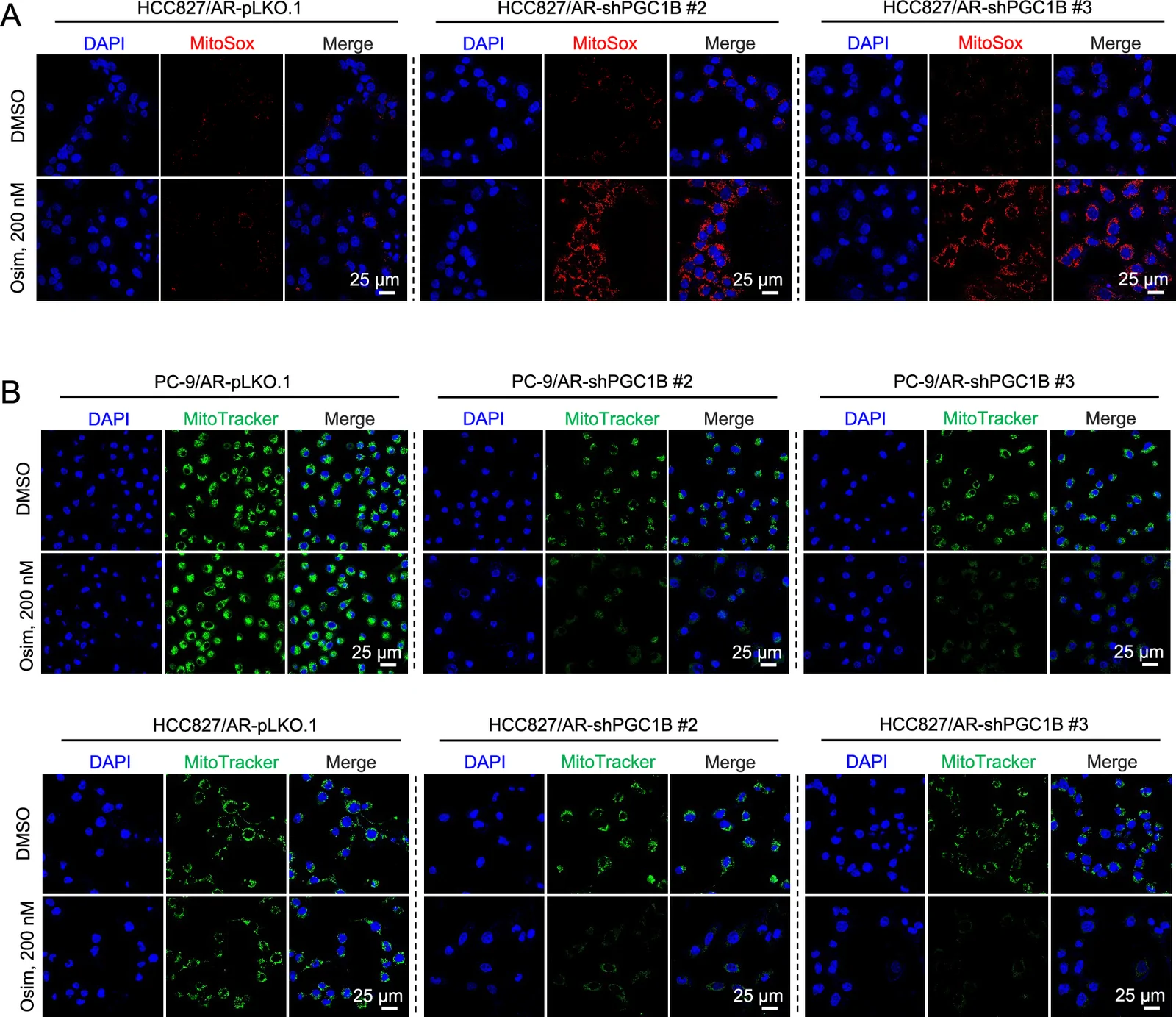
*PPARGC1B* (*PGC1B*) knockdown in osimertinib-resistant cells restores osimertinib’s ability to inhibit mitochondrial biogenesis. The indicated cell lines were exposed to 200 nM osimertinib (Osim) for 24 h and then stained with MitoSox Red dye for mitochondrial ROS (**A**) or MitoTracker Green FM dye for mitochondrial mass (**B**).

**Figure EV5:**
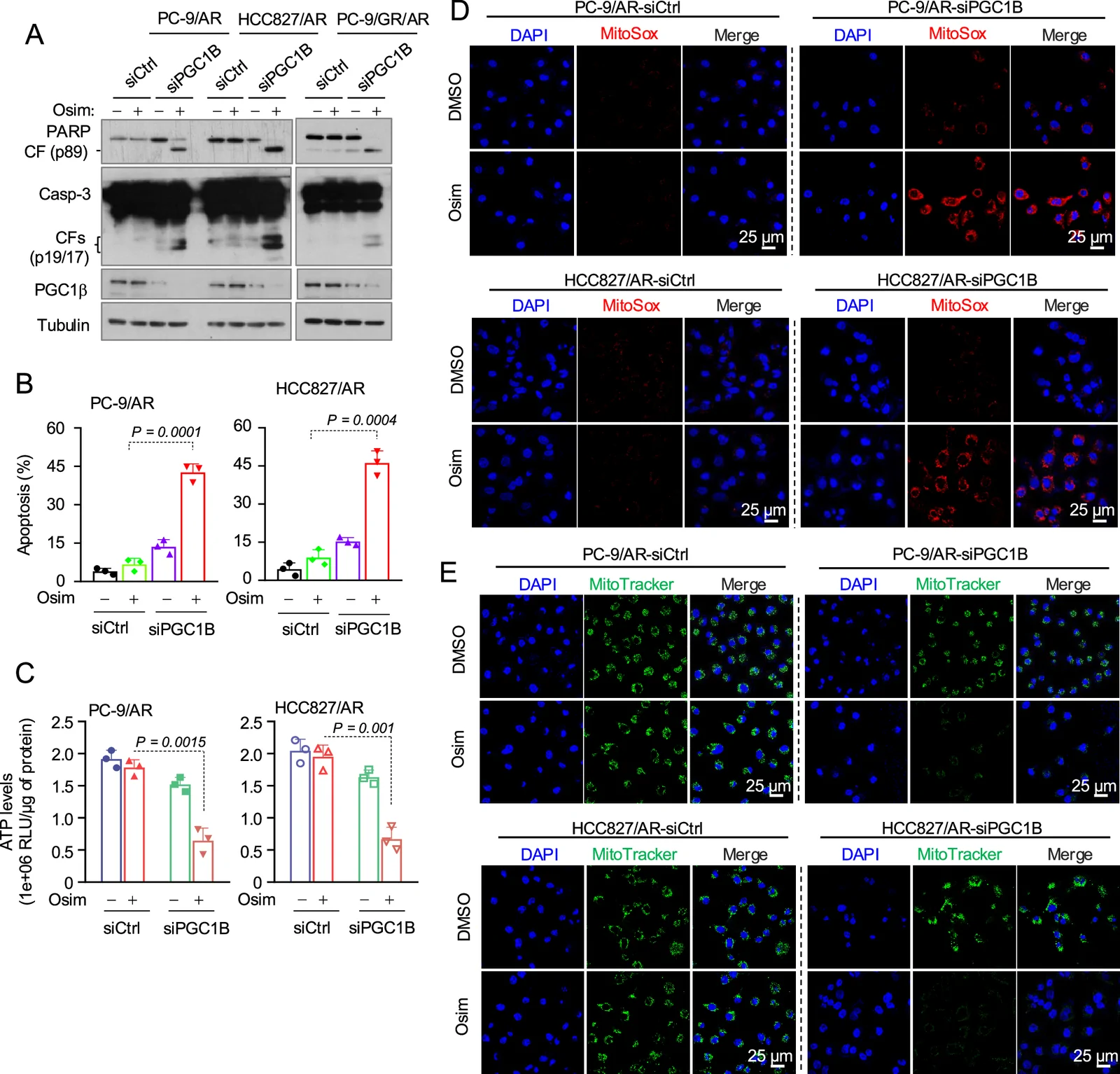
*PPARGC1B* (*PGC1B*) knockdown with siRNA in osimertinib-resistant cells sensitizes the cells to undergo apoptosis with suppression of mitochondrial biogenesis. The indicated cell lines were transfected with control or *PPARGC1B* (*PGC1B*) siRNA for 48 h, followed by treatment with 200 nM osimertinib for 48 h (**A**, **B**) or 24 h (**C**–**E**). The proteins of interest were detected with Western blotting (**A**). Annexin V-positive cells were analyzed with flow cytometry (**B**). ATP levels were measured using the Mitochondrial ToxGlo™ Assay (**C**). Mitochondrial ROS were detected by MitoSOX™ Red staining (**D**). Mitochondrial mass was detected by MitoTracker® Green FM staining (**E**). The data in (**B**, **C**) are means ± SDs of triplicate determinations. Statistical differences between the two given groups were evaluated with a two-sided unpaired Student’s *t* test.

To further illustrate the role of elevated *PPARGC1B* expression in osimertinib-acquired resistance, we used a genetic approach to reduce *PPARGC1B* expression in osimertinib-resistant cell lines through gene knockdown and then examined its impact on cell sensitivity to osimertinib. We speculated that enforced reduction of elevated PGC1β in the resistant cell lines should restore their sensitivity to osimertinib if the elevation is a critical event that mediates the emergence of acquired resistance to osimertinib. Indeed, while osimertinib was ineffective in inducing cleavage of PARP and caspase-3 (Fig. 7A), increasing annexin V-positive cells (Fig. 7B) and decreasing cell survival (Fig. 7C) in both PC-9/AR and HCC827/AR cells infected with control lentiviruses, it clearly did so in the cell lines infected with lentiviruses carrying different *PPARGC1B* shRNAs. In agreement, osimertinib significantly decreased mitochondrial ATP levels (Fig. 7D), increased MitoSox staining (ROS production; Figs. 7E and EV4A), and decreased MitoTracker Green staining (mitochondria mass; Fig. EV4B) in cells expressing *PPARGC1B* shRNAs, but had minimal or no effect in control cells. Similar results were also generated in the osimertinib-resistant cell lines transfected with *PPARGC1B* siRNA (Fig. EV5). These results together clearly indicate that enforced reduction of PGC1β through genetic knockdown of *PPARGC1B* expression in osimertinib-resistant cells restores their response to osimertinib in suppressing mitochondrial biogenesis with subsequent induction of apoptosis.

Mitochondrial transcription factor A (TFAM) is essential for mitochondrial biogenesis and integrity (Wang et al, 2026). We then determined whether enforced overexpression of ectopic *TFAM* attenuates the ability of *PPARGC1B* knockdown to sensitize osimertinib-resistant cells to osimertinib in terms of mitochondrial dysfunction and apoptosis induction. In both PC-9/AR and HCC827/AR cell lines, *PPARGC1B* knockdown (Appendix Fig. S2A) clearly decreased MitoTracker Green staining (Appendix Fig. S2B) and increased cPARP levels (Appendix Fig. S2A) upon osimertinib treatment in comparison with pLKO.1 control cells; however, these effects were reduced when the *TFAM* gene was co-expressed (Appendix Fig. S2), suggesting that enforced overexpression of ectopic *TFAM* partially rescues mitochondrial dysfunction and apoptosis induced by PGC1β suppression/osimertinib treatment in these osimertinib-resistant cells. These results further demonstrate a critical role of mitochondrial suppression in mediating osimertinib’s therapeutic efficacy.

Furthermore, we determined the impact of *PPARGC1B* knockdown in osimertinib-resistant cells on the response of tumors to osimertinib in vivo using PC-9/AR as a model. While osimertinib treatment caused limited growth suppression of PC-9/AR/pLKO.1 tumors, it significantly and effectively inhibited the growth of PC-9/AR/shPGC1B tumors as evaluated by measuring both tumor sizes (Fig. 7F,G) and weights (Fig. 7H). Mouse body weights among these groups did not show much difference (Fig. 7I). Hence, enforced reduction of PGC1β via gene knockdown clearly restores the response of osimertinib-resistant tumors to osimertinib in vivo.

### Combination of osimertinib with a mitochondria-targeting agent synergistically decreases the survival of osimertinib-resistant cells and augments the induction of Bim-dependent apoptosis and suppression of mitochondrial biogenesis

**Figure 8:**
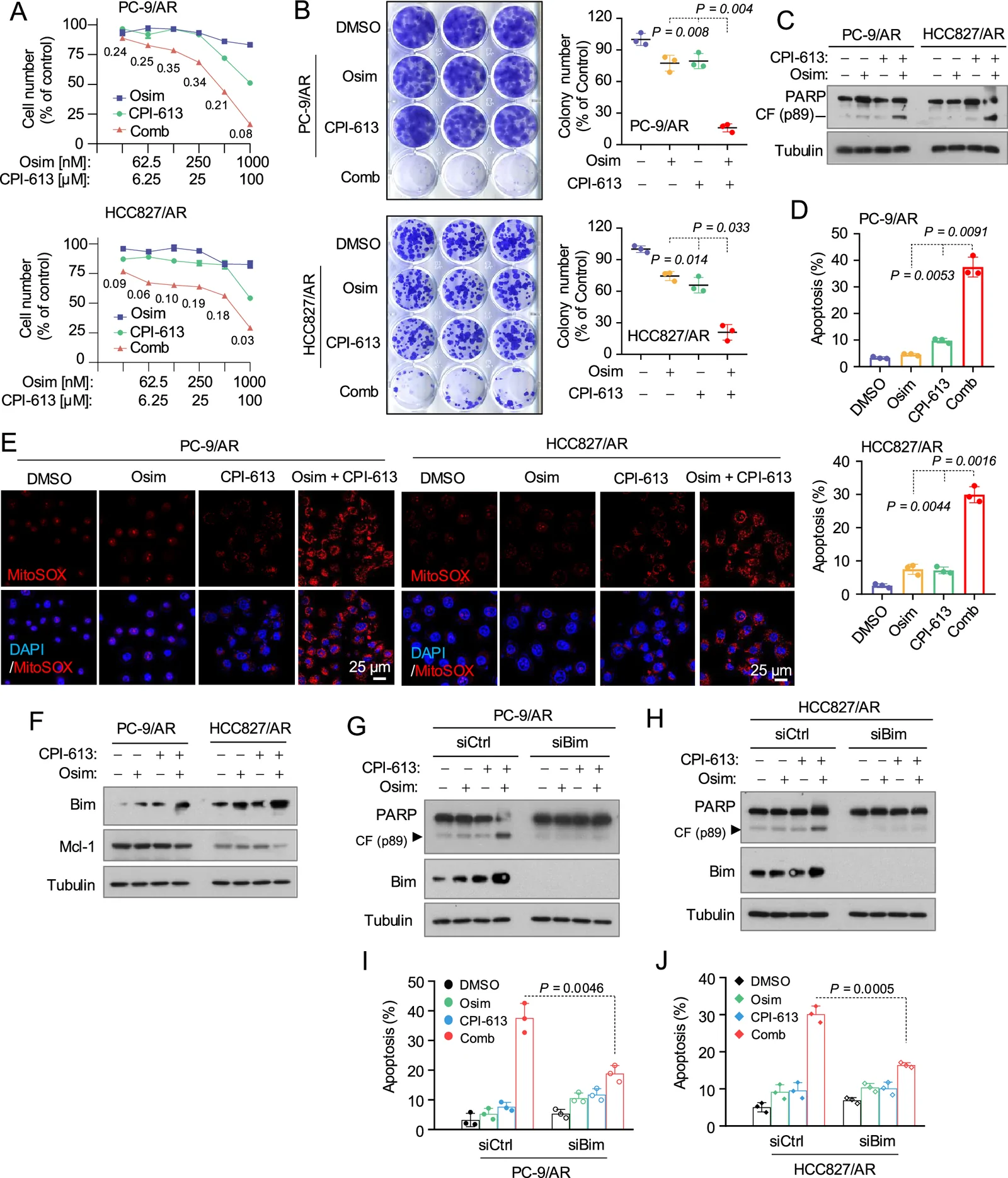
Combination of osimertinib with a mitochondria-targeting agent synergistically decreases the survival of osimertinib-resistant cells and augments the induction of Bim-dependent apoptosis and suppression of mitochondrial biogenesis. (**A**) Both PC-9/AR and HCC827/AR cell lines were treated with varied concentrations of the tested agents either alone or in combinations for 72 h. Cell numbers were then measured by the SRB assay, and combination index (CI) values were calculated and presented within the graph. The data are means ± SDs of four replicate determinations. (**B**) The indicated cell lines seeded in 12-well plates were exposed to 100 nM osimertinib, 250 nM CPI-613, or their combination for 10 days. The data are means ± SDs of triplicate treatments. (**C**–**E**) The tested cell lines were treated with 200 nM osimertinib, 5 µM CPI-613, or their combinations for 48 h (**C**, **D**) and 24 h (**E**). The proteins of interest were detected with western blotting (**C**). Apoptotic cells were analyzed with annexin V staining/flow cytometry (**D**). Mitochondrial ROS were detected by MitoSOX™ Red staining (**E**). The data are means ± SDs of triplicate determinations. (**F**) The indicated cell lines were exposed to 200 nM osimertinib, 5 µM CPI-613, or their combinations for 16 h and then harvested for subsequent western blot analysis of the proteins of interest. (**G**–**J**) The indicated cell lines were transfected with control (Ctrl) or Bim siRNA for approximately 48 h and then treated with 200 nM osimertinib, 5 µM CPI-613, or their combinations for 48 h. The proteins of interest were detected with Western blotting (**G**, **H**). Apoptotic cells were detected with annexin V staining/flow cytometry (**I**, **J**). Each column represents mean ± SD of triplicate treatments. Statistical differences between two given groups and among multiple groups were conducted with a two-sided unpaired Student’s *t* test and a one-way ANOVA test, respectively. Source data are available online for this figure.

**Figure EV6:**
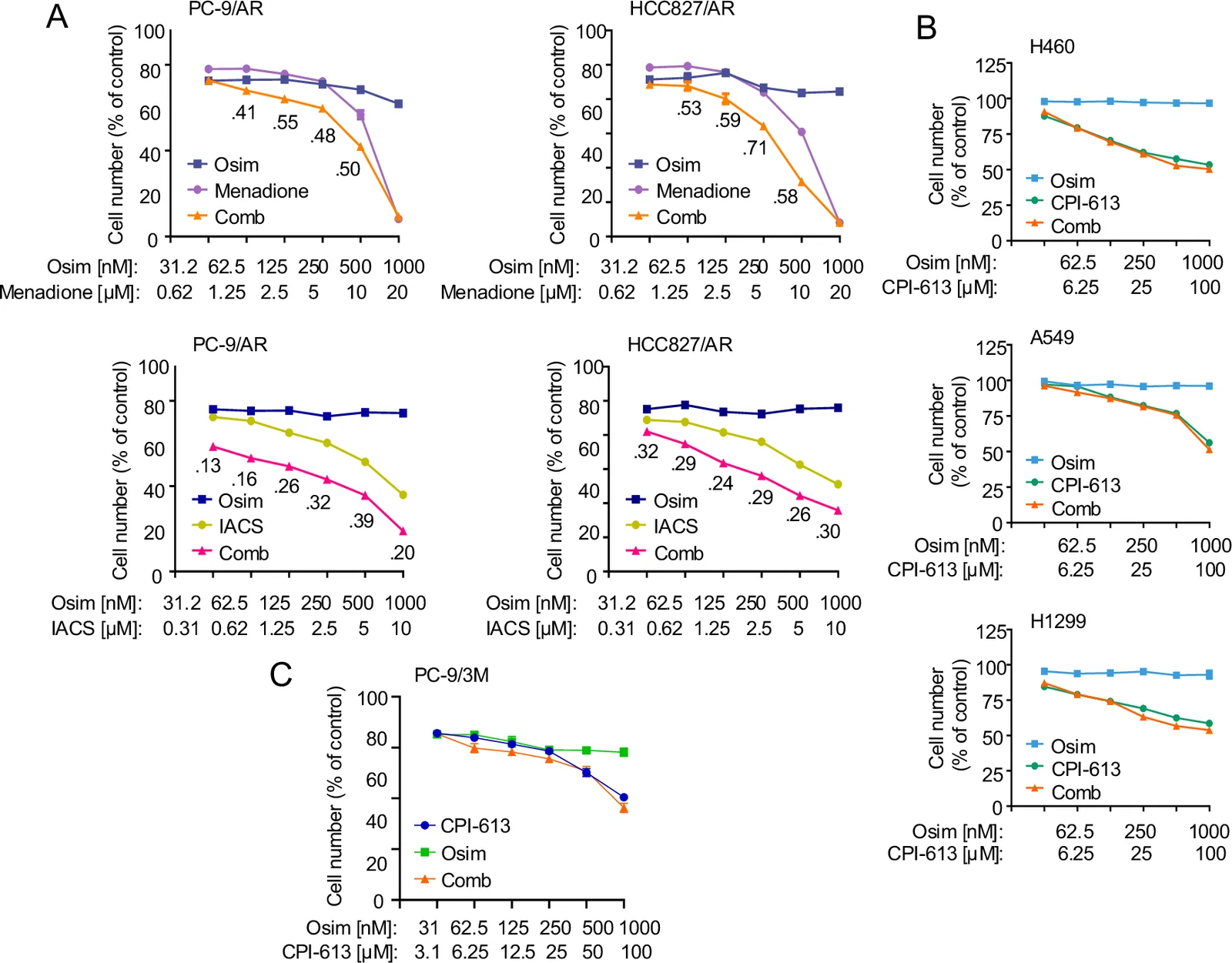
Effects of osimertinib combined with a mitochondria-targeting agent on the survival of EGFRm NSCLC cell lines with acquired resistance to osimertinib and NSCLC cell lines with WT EGFR. The indicated cell lines with mutant (**A**, **C**) or WT (**B**) EGFR seeded in 96-well plates were treated with different concentrations of the tested agents alone or in combination for 3 days. Cell numbers were then estimated with the SRB assay. The data are means ± SDs of four replicate determinations. IACS, IACS-010759.

Since there are no small-molecule inhibitors that specifically target PGC1β, it is hard to translate our findings in the clinic by directly targeting PGC1β. We therefore considered potential anti-tumor agents that target mitochondria, given the positive connection between PGC1β and mitochondrial biogenesis (Villena, 2015) and that some mitochondria-targeting agents have been tested in clinical trials (Dong et al, 2020; Vasan et al, 2020). In the above studies, we have demonstrated that osimertinib induces inhibition of PGC1β-dependent mitochondrial biogenesis. We thus tested the effects of osimertinib in combination with mitochondria-targeting agents, including CPI-613, menadione, and IACS-010759, on the survival of PC-9/AR and HCC827/AR cells. These combinations were all more active than each agent alone in decreasing the survival of the tested osimertinib-resistant cell lines with CIs of <1 (Figs. 8A and EV6A), showing synergistic effects. We thus focused on CPI-613, which is currently being tested in phase 3 clinical trials (Anderson et al, 2022; Dong et al, 2020; Mangieri et al, 2023; Mohan et al, 2023; Philip et al, 2024; Vasan et al, 2020), for further evaluation. In colony formation assay, the CPI-613 and osimertinib combination effectively inhibited the formation and growth of both PC-9/AR and HCC827/AR colonies, whereas each agent alone did not (Fig. 8B). In agreement, the combination was more active than each agent alone in inducing PARP cleavage (Fig. 8C) and increasing annexin V-positive cells (Fig. 8D) in both PC-9/AR and HCC827/AR cells, indicating enhanced induction of apoptosis. Moreover, the combination was also more effective than either single agent in enhancing MitoSox staining intensity or mitochondrial ROS (Fig. 8E) in these osimertinib-resistant cell lines, suggesting that the combination enhances the suppression of mitochondrial biogenesis as well in the osimertinib-resistant cells. When testing the effects of osimertinib and CPI-613 combination on the survival of other NSCLC cell lines without mutant EGFR, we found that the combination did not enhance the decrease in survival of these EGFR WT NSCLC cell lines (Fig. EV6B). Hence, it appears that CPI-613 in combination primarily augments the ability of osimertinib to decrease the survival of osimertinib-resistant EGFRm NSCLC cells. Since EGFR C797S mutation prevents osimertinib from binding to mutant activating EGFR, it is reasonable to see that osimertinib and CPI-613 combination was not active in PC-9/3M cell line that carries a C797S mutation (Fig. EV6C).

To gain insight into how the combination enhances the induction of apoptosis in these osimertinib-resistant cell lines, we further assessed the effects of the combination on the modulation of Bim and Mcl-1, critical events that mediate the induction of apoptosis by osimertinib in EGFRm NSCLC cells (Shi et al, 2017). The combination was more potent than every single agent in decreasing Mcl-1 levels and, particularly, increasing Bim levels in both PC-9/AR and HCC827/AR cell lines (Fig. 8F). Knockdown of Bim in both osimertinib-resistant cell lines abrogated or attenuated the effects of the combination on enhancing PARP cleavage (Fig. 8G,H) and increasing annexin V-positive cells (Fig. 8I,J). These results suggest that the combination enhances Bim-dependent induction of apoptosis in osimertinib-resistant cells.

### Combination of osimertinib with a mitochondria-targeting agent effectively inhibits the growth of osimertinib-resistant tumors in vivo

**Figure 9:**
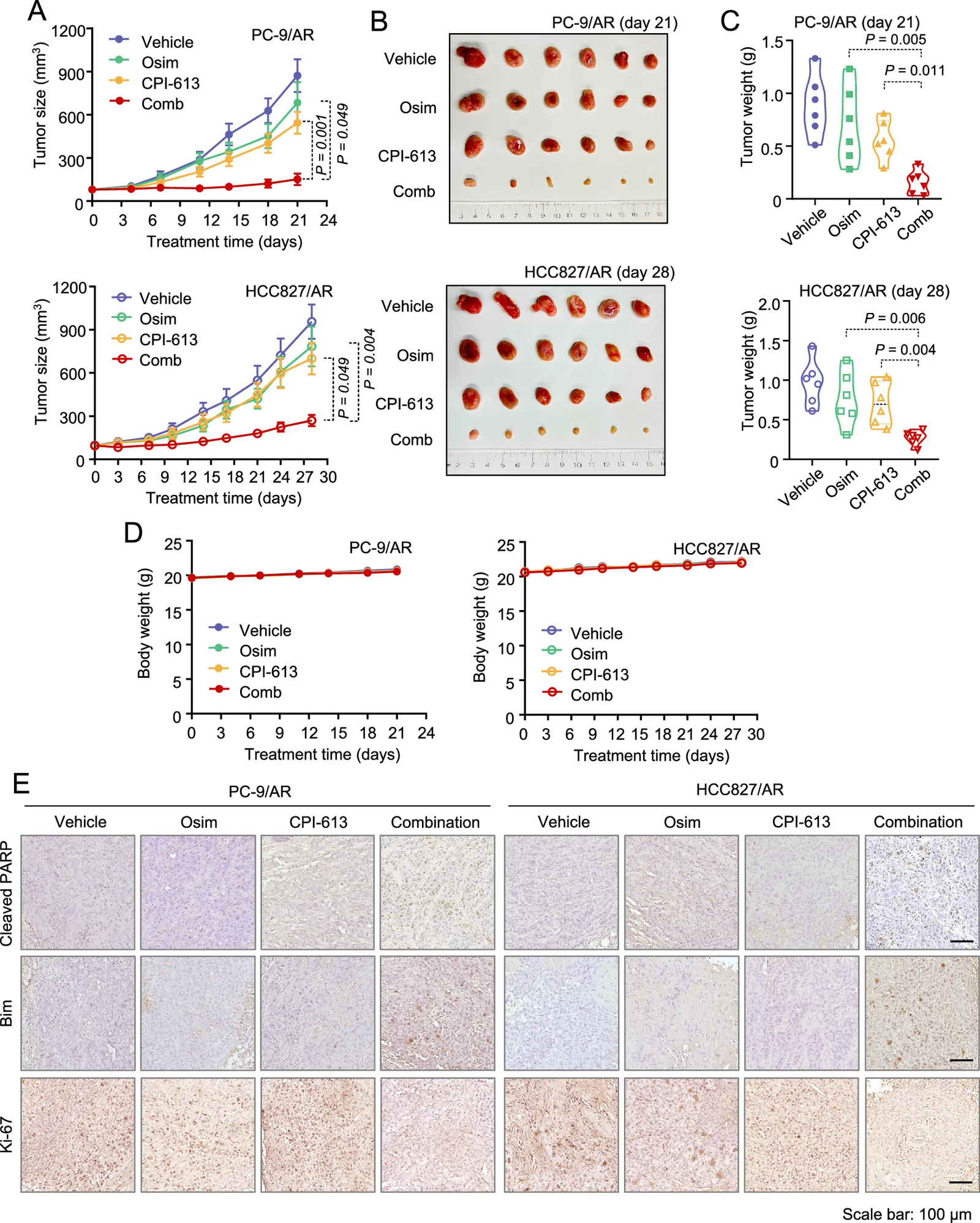
Combination of osimertinib with CPI-613 effectively inhibits the growth of osimertinib-resistant tumors in vivo. Both PC-9/AR and HCC827/AR cells grown in NU/NU nude mice as xenografted tumors (*n* = 6/group) were treated with vehicle, osimertinib alone (5 mg/kg, o.g., daily), CPI-613 (25 mg/kg, i.p., daily), or their combination. Tumor sizes (**A**) were measured at the indicated time points. At the end of the treatments, tumors in each group were photographed (**B**) and weighed (**C**). Mouse body weights were also recorded every 3 days as indicated (**D**). The data in each group are means ± SEs (*n* = 6). The proteins of interest as indicated in tumor tissues were stained with IHC (**E**). Statistical differences between two given groups and among multiple groups were conducted with two-sided unpaired Student’s *t* test and one-way ANOVA test, respectively. Source data are available online for this figure.

**Figure EV7:**
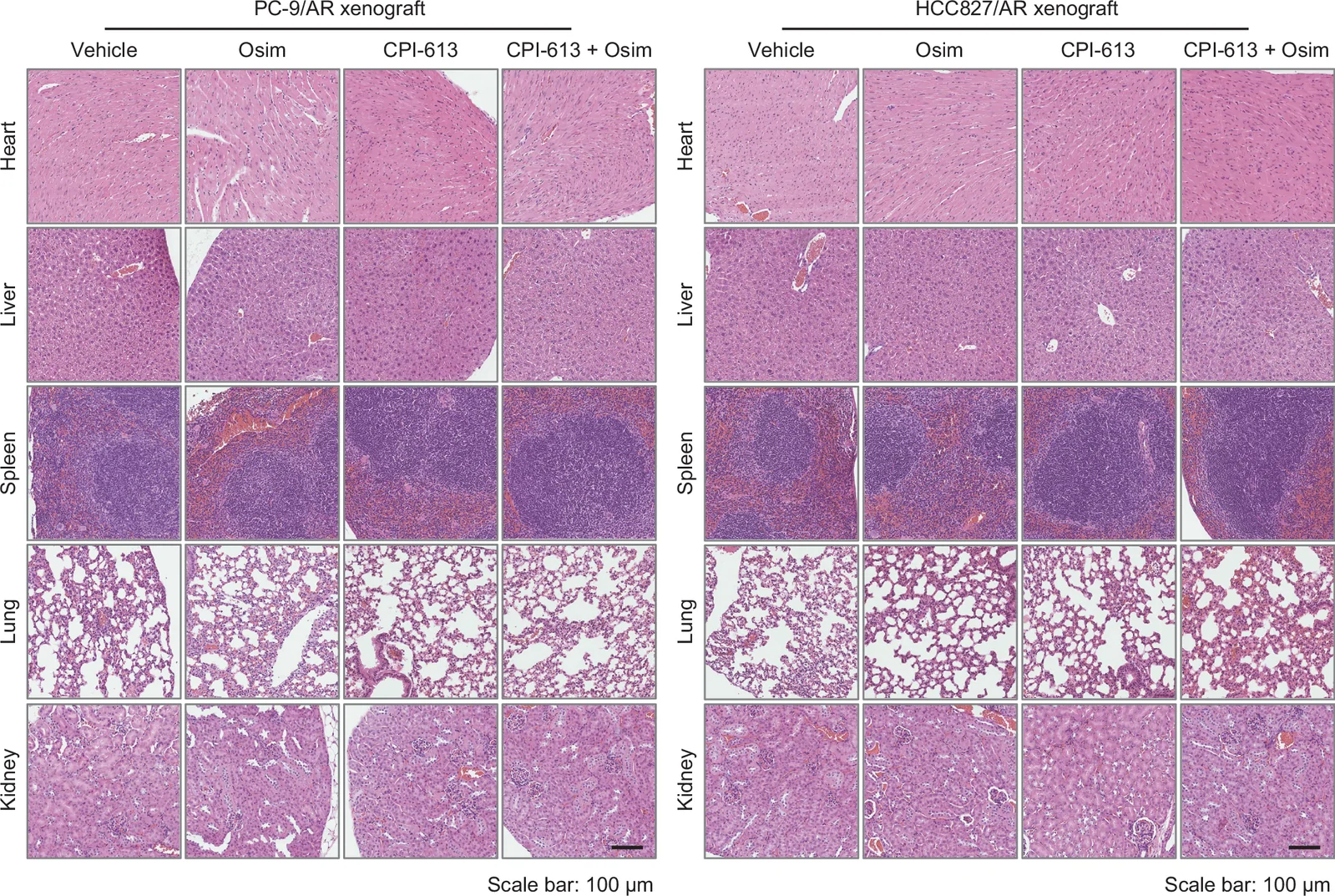
The combination of osimertinib and CPI-613 does not show harmful effects on the major organs of mice. The treatments were the same as described in Fig. 9. The morphologies of the tested organs as indicated were evaluated with H&E staining.

**Figure EV8:**
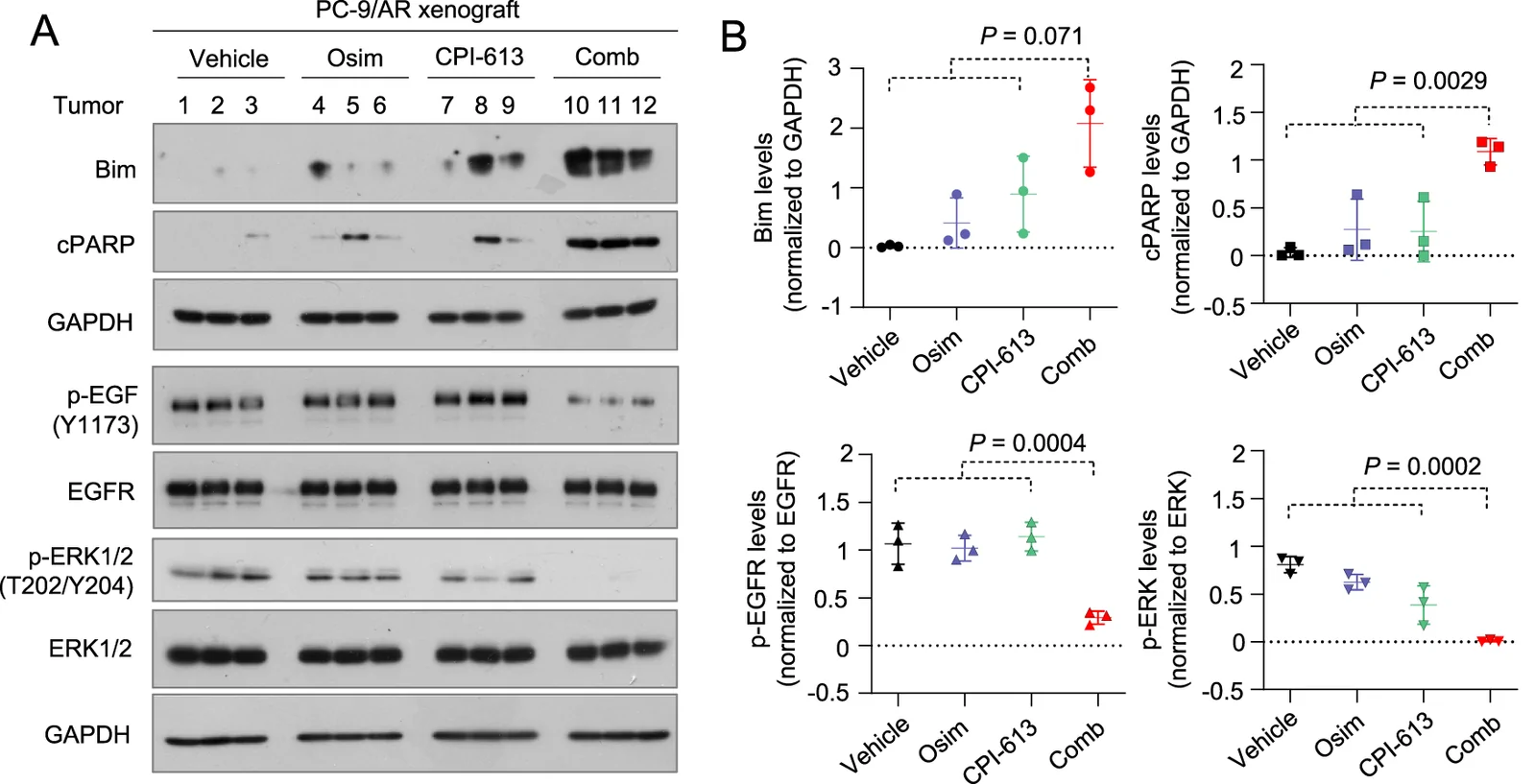
Detection of protein markers in PC-9/AR tumor tissues using western blotting. The tumor treatments are the same as described in Fig. 9. Proteins of interest were detected with western blotting (**A**). Protein quantification was conducted using ImageJ software (**B**). The data is presented as means ± SDs generated from three different tumor samples. Statistical differences among the treatment groups were evaluated with a one-way ANOVA test.

Following the above in vitro studies, we further evaluated the effect of the osimertinib and CPI-613 combination on the growth of osimertinib-resistant tumors in mice. While osimertinib and CPI-613 alone led to limited inhibition of the growth of osimertinib-resistant PC-9/AR tumors, the combination of osimertinib and CPI-613 was much more active than each agent alone and significantly inhibited the growth of PC-9/AR tumors as measured by tumor sizes (Fig. 9A,B) and tumor weights (Fig. 9C). Similar results were also generated in the HCC827/AR tumor model (Fig. 9A–C). In both tumor models, mice receiving treatment with the combination of osimertinib and CPI-613 had comparable body weights with mice in other treatment groups (Fig. 9D). Hematoxylin and eosin (H&E) staining of mouse tissues from different organs, including lung, liver, kidney, spleen, and heart, did not show differences among the treatment groups (Fig. EV7). These results together demonstrate the favorable tolerability of the osimertinib and CPI-613 combination. IHC detection of Bim and cleaved PARP (cPARP) showed that cells positive for Bim or cPARP staining were clearly increased in tumors treated with CPI-613 and osimertinib, but showed limited or no increase in tumors receiving osimertinib or CPI-613 alone (Fig. 9E), indicating that the combination of CPI-613 and osimertinib enhances the induction of apoptosis in osimertinib-resistant tumors in vivo. This is further confirmed by Western blot analysis of these tumor tissues, as the combination of osimertinib and CPI-613 was much more active than either agent alone in increasing the levels of both Bim and cPARP (Fig. EV8). Moreover, the combination decreased the levels of p-EGFR and p-ERK, whereas either agent alone did not do so (Fig. EV8), suggesting that the combination enhances suppression of the EGFR signaling. In addition, the expression of Ki67, a well-known proliferation marker, was also decreased in tumors treated with CPI-613 and osimertinib combination, but not or limited reduced in tumors receiving osimertinib or CPI-613 alone (Fig. 9E). Hence, the combination of osimertinib and CPI-613 effectively inhibits tumor cell proliferation as well.

## Discussion

The current study has revealed a previously undiscovered connection between PGC1β downregulation/mitochondrial biogenesis inhibition and the mediation of the therapeutic efficacy of osimertinib in EGFRm NSCLC cells. The findings have demonstrated that the inhibition of PGC1β/mitochondrial biogenesis is a critical event contributing to the therapeutic efficacy of osimertinib in EGFRm NSCLC cells, whereas rebound elevation of PGC1β upregulation and increased mitochondrial biogenesis are tightly associated with the emergence of acquired resistance to osimertinib. Accordingly, targeting mitochondrial biogenesis using small-molecule inhibitors such as CPI-613 in combination with osimertinib effectively overcomes osimertinib acquired resistance as demonstrated using both in vitro and in vivo models. CPI-613 is currently being tested in phase 3 clinical trials as a potential anticancer agent primarily against pancreatic cancer (Anderson et al, 2022; Dong et al, 2020; Mangieri et al, 2023; Mohan et al, 2023; Philip et al, 2024; Vasan et al, 2020). These trials primarily focused on the combination of CPI-613 with different chemotherapeutic agents (Anderson et al, 2022; Mangieri et al, 2023; Mohan et al, 2023; Philip et al, 2024). Our findings provide a strong scientific rationale for using CPI-613 in combination with osimertinib to improve EGFR-targeted therapy through overcoming the clinically challenging issue of acquired resistance. When designing future trials using CPI-613 in combination with osimertinib, the dosage range of CPI-613 used in the published combinational clinical trials should be considered. Moreover, patients with osimertinib-relapsed EGFRm NSCLC expressing elevated PGC1β should be a priority population for this therapeutic strategy. Hence, future clinical trials that validate our preclinical findings in this direction are warranted. From this point of view, our findings are of high translational significance.

We previously showed that osimertinib inhibits *TNFRSF1A* (*DR4*) transcription through suppression of the MEK/ERK/AP-1 signaling pathway, including downregulation of both c-Jun and FOSL1 (Zhang et al, 2021). While studying FOSL1/AP-1 regulation of *PPARGC1B* expression, a recent study using a multi-omics approach also demonstrated that osimertinib inhibits AP-1 activity in EGFRm NSCLC primarily through downregulation of both FOSL1 and c-Jun expression and that AP-1 activation is a mediator of osimertinib acquired resistance (Dayanc et al, 2025). Hence, our findings of FOSL1 and c-Jun downregulation and AP-1 suppression by osimertinib in EGFRm NSCLC cells agree with those reported in previous studies (Dayanc et al, 2025; Zhang et al, 2021). The novel finding in this study is the FOSL1/AP-1-mediated transcription of *PPARGC1B* based on the following results: (1) modulation of FOSL1 levels through gene overexpression or knockdown alters the levels of PGC1β; and (2) functional AP-1 binding sites or TREs are present in the 5’flanking region of the *PPARGC1B* gene and mediate suppression of *PPARGC1B* transcription by osimertinib. This novel finding provides mechanistic insight into the transcriptional regulation of the *PPARGC1B* gene, increasing our understanding of the biology of PGC1β. FOSL1 is known to be regulated transcriptionally and post-translationally through the MEK/ERK signaling pathway (Casalino et al, 2003; Dikshit et al, 2018; Wang et al, 2017). Therefore, osimertinib-induced downregulation of FOSL1/AP-1-mediated *PPARGC1B* expression is likely to be a downstream event of MEK/ERK inhibition (Figs. 3F, 4G).

It was previously reported that hTERT/telomere dysfunction is involved in the suppression of *PPARGC1B* expression and subsequent mitochondrial metabolism via activation of p53 (Sahin et al, 2011). We previously reported that osimertinib decreases c-Myc levels through induction of ERK suppression-caused c-Myc degradation (Zhu et al, 2021). Moreover, we recently further demonstrated that osimertinib inhibits c-Myc-mediated *hTERT* expression with induction of telomere dysfunction and telomeric DNA damage in EGFRm NSCLC cells (Chen et al, 2024). In this study, we have revealed a novel mechanism by which hTERT regulates *PPARGC1B* expression through a p53-independent, but FOSL-dependent mechanism (Fig. 4G) since enforced overexpression of FOSL1 in EGFRm NSCLC cell lines with mutant p53 rescued PGC1β reduction caused by hTERT inhibition. Our results do not conclusively demonstrate whether hTERT has a direct effect on the regulation of FOSL1 expression (Fig. 4G). It was shown that hTERT knockdown decreases the expression of both FOSL1 and EGFR in bladder cancer (Kraemer et al, 2006). In our study, hTERT knockdown also decreased EGFR levels with suppressed EGFR signaling. However, osimertinib decreased EGFR levels at relatively later times in comparison with hTERT and FOSL1 reduction, which occurred earlier than EGFR downregulation. Therefore, it is likely that the inhibition of hTERT causes a reduction of EGFR levels at a late stage, leading to suppression of MEK/ERK signaling, further boosting inhibition of FOSL1/AP-1-mediated *PPARGC1B* expression in EGFRm NSCLC cells (Fig. 4G). This may explain why FOSL1 levels are rapidly and sustainedly reduced by osimertinib in EGFRm NSCLC cells.

Telomerase inhibition/telomere dysfunction was reported to activate p53, leading to suppression of *PPARGC1B* expression in mice (Sahin et al, 2011). Our finding on hTERT regulation of *PPARGC1B* expression via the EGFR/FOSL1 axis, as discussed above, is clearly p53-independent since all the tested cell lines possess mutant p53. Interestingly, this effect is likely independent of telomerase. Hence, our findings provide a scientific insight into how hTERT is involved in the regulation of *PPARGC1B* expression and subsequent mitochondrial biogenesis in the context of mutant p53.

In this study, PGC1β levels were apparently elevated in different osimertinib-resistant cell lines that lost response to osimertinib treatment. Moreover, enforced overexpression of ectopic *PPARGC1B* in the sensitive EGFRm NSCLC cells compromised cell response to osimertinib, whereas genetic knockdown of *PPARGC1B* expression in osimertinib-resistant cell lines restored their sensitivities to osimertinib. Hence, the rebound elevation of PGC1β clearly plays an important role in mediating the emergence of osimertinib-acquired resistance. PGC1β elevation was detected in just over 57% of EGFRm NSCLC tissues from patients whose disease relapsed from treatment with EGFR-TKIs, including osimertinib, thus validating our in vitro findings. Given the heterogeneous mechanisms accounting for osimertinib acquired resistance, it can be logically speculated that other resistance mechanisms may play dominant roles in EGFRm NSCLCs relapsed from osimertinib treatment in which PGC1β expression is not increased. In this study, PGC1β elevation was detected in osimertinib-resistant cell lines with different resistant mechanisms, including *EGFR C797S* mutation (PC-9/3 M) and *MET*-amplification (HCC827/AR). Although additional gene mutations were detected in HCC827- and PC-9-derived osimerntinb-resistant cells lines with varied patterns (Appendix Fig. S3), it is hard to see any potential impact of these mutations on *PPARGC1B* expression in the osimertinib-resistant cell lines because PGC1β levels were elevated in these resistant cell lines regardless of these gene mutations. In agreement, PGC1β elevation in EGFRm NSCLC tissues that relapsed from EGFR-TKI treatment was not associated with any new gene mutations. Hence, PGC1β rebound elevation is likely to represent a common resistance mechanism to osimertinib. Whether other non-genetic resistance mechanisms impact PGC1β rebound elevation in osimertinib-resistant cells/tissues may need further investigation. Nonetheless, the combination strategy of osimertinib with a mitochondria-targeting agent such as CPI-613 may work better in patients with relapsed NSCLC expressing elevated PGC1β. Therefore, to achieve possible clinical benefit, the detection of PGC1β elevation may be used as a predictive biomarker for selecting patients with disease relapse from osimertinib treatment to receive this therapeutic strategy.

*TP53* and *EGFR* co-mutation occur in about 45% NSCLC (17-73%) and is often associated with reduced response rates and shorter survival when treated with EGFR-TKIs, including osimertinib (Liu et al, 2022; Wei et al, 2025). The finding that the combination of osimertinib and CPI-613 effectively inhibited the growth of EGFRm osimertinib-resistant tumors with a *TP53* co-mutation (e.g., PC-9/AR) implies its potential application in the treatment of this group of NSLNCs relapsed from osimertinib treatment, particularly those with elevated PGC1β.

In the tested osimertinib-resistant cell lines, *PPARGC1B* mRNA expression and FOSL1 levels were also increased, together with elevated PGC1β. Moreover, the knockdown of FOSL1 in osimertinib-resistant cell lines reduced the levels of PGC1β. Thus, these findings suggest that elevated PGC1β in osimertinib-resistant cells is likely due to the increased gene transcription mediated by increased FOSL1 and activated AP-1. It was recently reported that AP-1 is a targetable vulnerability of osimertinib acquired resistance, and targeting this vulnerability is a potential strategy to reverse acquired resistance (Dayanc et al, 2025). AP-1 has long been considered a potential cancer target. However, efforts in this direction to develop AP-1 inhibitors have not achieved success in moving forward the inhibitors to clinical trials or application because of the complex structures and functions of AP-1 (Atsaves et al, 2019; Casalino et al, 2022; Karin, 1995; Song et al, 2023; Xue et al, 2025). Moreover, the potential concern of possible side effects and toxicity caused by targeting AP-1 that is involved in the regulation of various biological processes via different target genes should be considered. In the case of overcoming acquired resistance to EGFR-targeted therapy, targeting the downstream events of AP-1, PGC1β/mitochondrial biogenesis, may be a reasonable and realistic strategy, particularly in the situation that the tested CPI-613 is a candidate drug that has been moved to phase II/III trials (Anderson et al, 2022; Dong et al, 2020; Philip et al, 2024; Steiner et al, 2025; Vasan et al, 2020).

**Figure EV9:**
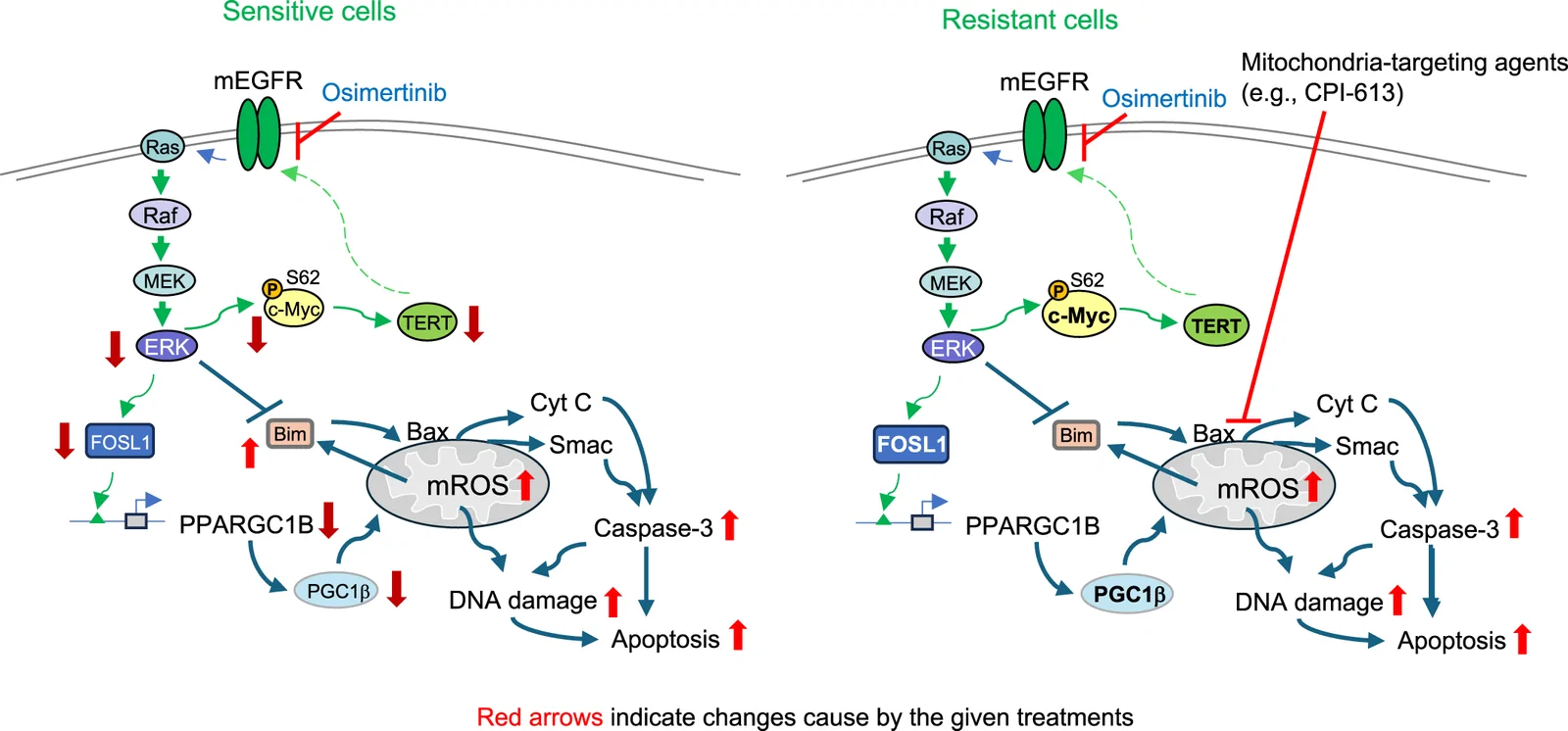
Schemas for downregulation of FOSL1-dependent regulation of PPARGC1B expression, suppression of mitochondrial biogenesis, such as mitochondrial ROS (mROS) generation, and induction of Bim-dependent apoptosis in osimertinib-sensitive cells (left panel) and for restoration of this mechanism through co-targeting mitochondria using a mitochondria-targeting agent such as CPI-613 in osimertinib-resistant cells (right panel), respectively. Cyt C cytochrome C.

The central role of mitochondria in the regulation of apoptosis is well-known, and this regulation is critically mediated by Bim and ROS (Redza-Dutordoir and Averill-Bates, 2016; Sun, 2022). ERK suppression-mediated Bim elevation and Mcl-1 (Liu et al, 2022) reduction are critical mechanisms accounting for osimertinib-induced apoptosis, as we previously demonstrated (Shi et al, 2017). In agreement, the current study found that the osimertinib and CPI-613 combination augmented Bim elevation and Mcl-1 reduction in EGFRm osimertinib-resistant cell lines and enhanced Bim-dependent apoptosis. Since mitochondrial ROS production is known to increase Bim expression and its translocation to mitochondria (Deng et al, 2015; Hagenbuchner et al, 2012; Khawaja et al, 2008) and causes DNA damage (Redza-Dutordoir and Averill-Bates, 2016), the current finding on the induction of mitochondrial ROS generation by osimertinib in EGFRm NSCLC cells is likely to further boost Bim elevation and Bim-dependent apoptosis together with enhanced DNA damage (Fig. EV9, left panel). Given that the elevated FOSL/PGC1β in osimertinib-resistant cells compromises this effect, the addition of a mitochondrial-targeting agent such as CPI-613 may restore this effect and trigger apoptosis in osimertinib-resistant cells (Fig. EV9, right panel).

In summary, the current study has demonstrated a critical role of the inhibition of FOSL1/AP-1-dependent PGC1β/mitochondrial biogenesis in mediating the therapeutic efficacy of EGFR-targeted therapy, primarily using osimertinib as an example in EGFRm NSCLC. Moreover, targeting PGC1β or mitochondrial biogenesis is likely to be a promising therapeutic strategy for improving the therapeutic outcomes of EGFR-targeted therapy via overcoming acquired resistance. The clinical validation of this strategy is warranted. The findings in this study also provide scientific insights into how FOSL1/AP-1 regulates the expression of *PPARGC1B* and subsequent mitochondrial biogenesis.

## Methods

**Table:** Reagents and tools table

Reagent/resource	Reference or source	Identifier or catalog number
**Experimental models**		
NU/J athymic nude mice	The Jackson Laboratory	Stock No. 002019; RRID:IMSR_JAX:002019
PC-9 human NSCLC cells	Dr. P. A. Jänne	N/A
HCC827 human NSCLC cells	Dr. P. A. Jänne	N/A
PC-9/GR cells	Dr. P. A. Jänne	N/A
PC-9/AR cells	Shi et al, 2017	N/A
HCC827/AR cells	Shi et al, 2017	N/A
PC-9/GR/AR cells	Shi et al, 2017	N/A
PC-9/3M (EGFR 19del/T790M/C797S triple-mutant) cells	Dr. Aaron N. Hata	N/A
H1975/AR cells	Dr. Christine M. Lovly	N/A
SH418/AR cells	Dr. Christine M. Lovly	N/A
SH450/AR cells	Dr. Christine M. Lovly	N/A
19.1 Del murine EGFR-mutant NSCLC cells	Dr. Raphael A. Nemenoff	N/A
19.2 Del murine EGFR-mutant NSCLC cells	Dr. Raphael A. Nemenoff	N/A
L860R murine EGFR-mutant NSCLC cells	Dr. Raphael A. Nemenoff	N/A
**Recombinant DNA**		
PPARGC1B Lentiviral Vector (h)	Applied Biological Materials Inc.	Cat# LC137300
Fra1 Lentiviral Vector (h)	Applied Biological Materials Inc.	Cat# LC120747
PGC-1β shRNA Plasmid (h)	Santa Cruz Biotechnology	Cat# sc-62783-SH
Fra1 shRNA Plasmid (h)	Santa Cruz Biotechnology	Cat# sc-35405-SH
TERT shRNA Plasmid (h)	Santa Cruz Biotechnology	Cat# sc-36641-SH
TFAM Lentiviral Vector (h)	VectorBuilder	Custom construct
PPARGC1B promoter luciferase reporter (−2000/ + 1)	VectorBuilder	Custom construct
PPARGC1B promoter luciferase reporter (−1200/ + 1)	VectorBuilder	Custom construct
PPARGC1B promoter luciferase reporter (−800/ + 1)	VectorBuilder	Custom construct
PPARGC1B promoter luciferase reporter (−300/ + 1)	VectorBuilder	Custom construct
**Antibodies**		
Rabbit anti-PGC1β	Fortis Life Sciences	Bethyl, Cat# A302-274A
Rabbit anti-TERT	Affinity Biosciences	Cat# DF7129
Rabbit anti-FOSL1	Cell Signaling Technology	Cat# 5281
Rabbit anti-c-Jun	Cell Signaling Technology	Cat# 9165
Rabbit anti-EGFR	Cell Signaling Technology	Cat# 2232
Rabbit anti-Phospho-EGF Receptor (Tyr1173)	Cell Signaling Technology	Cat# 4407
Rabbit anti-PARP	Cell Signaling Technology	Cat# 9542
Rabbit anti-Erk1/2	Cell Signaling Technology	Cat# 9102
Rabbit anti-Phospho-Erk1/2 (Thr202/Tyr204)	Cell Signaling Technology	Cat# 9101
Rabbit anti-Cleaved PARP (Asp214) for WB	Cell Signaling Technology	Cat# 9541
Rabbit anti-Cleaved PARP (Asp214) (D64E10) for IHC	Cell Signaling Technology	Cat# 5625
Rabbit anti-Caspase-3	Cell Signaling Technology	Cat# 9662
Rabbit anti-TFAM	Cell Signaling Technology	Cat# 7495
Rabbit anti-Akt	Cell Signaling Technology	Cat# 9272
Rabbit anti-Phospho-Akt (Ser473)	Cell Signaling Technology	Cat# 9271
Mouse anti-Mcl-1	Santa Cruz Biotechnology	Cat# sc-12756
Rabbit anti-Bim	MilliporeSigma	Cat# SAB5702197
Rabbit anti-Ki-67 antibody (SP6)	Thermo Fisher Scientific	Cat# MA5-14520
Mouse anti-GAPDH	Proteintech	Cat# 60004-1-Ig
Mouse anti-α-Tubulin	Proteintech	Cat# 66031-1-Ig
Donkey anti-Rabbit IgG (H + L) Highly Cross-Adsorbed Secondary Antibody, Alexa Fluor™ 488	Thermo Fisher Scientific	Cat# A-21206
**Oligonucleotides and other sequence-based reagents**		
PPARGC1B siRNA (h)	Santa Cruz Biotechnology	Cat# sc-62783
Fra1 siRNA (h)	Santa Cruz Biotechnology	Cat# sc-35405
TERT siRNA (h)	Santa Cruz Biotechnology	Cat# sc-36641
**Chemicals, enzymes, and other reagents**		
CPI-613	MedChemExpress	Cat# HY-15453
IACS-010759 (OxPhos)	MedChemExpress	Cat# HY-112037
Menadione	MedChemExpress	Cat# HY-B0332
Imetelstat	Geron Corporation	Provided by the company
**Software**		
GraphPad Prism	GraphPad Software	Version 10
ImageJ/Fiji	National Institutes of Health	RRID:SCR_002285
FlowJo	BD Biosciences	Version 10
**Other**		
MitoTracker™ Green FM	Thermo Fisher Scientific	Cat# M7514
MitoSOX™ Mitochondrial Superoxide Indicators	Thermo Fisher Scientific	Cat# M36007
Hoechst 33342	Thermo Fisher Scientific	Cat# 62249
DAPI	Thermo Fisher Scientific	Cat# 62248
Mitochondrial ToxGlo™ Assay	Promega	Cat# G8001
Dual-Luciferase Reporter Assay System	Promega	Cat# E1910

### Cell lines and cell culture

All human NSCLC cell lines including those with acquired resistance cell lines (Appendix Table S1) used in this study were the same as described previously (Chen et al, 2021; Ichihara et al, 2017; Shi et al, 2017; Shi et al, 2016). PC-9/AR (AZD9291-resistant), HCC827/AR, and PC-9/GR (gefitinib-resistant)/AR were established by exposing PC-9, PC-9/GR and HCC827 cell lines to gradually increased concentrations of osimertinib starting from 10 nM until up to 500 nM for around 6 months as described previously (Shi et al, 2017; Shi et al, 2016). The resistance was stable even after withdrawal of osimertinib for over 6 months (Shi et al, 2017; Shi et al, 2016). Common cancer gene mutations or alterations in these cell lines were detected using PredicineATLAS 600 cancer gene panel NGS assay in Predicine, Inc. (Hayward, CA), and the results were presented in Appendix Fig. S3. H1975/AR, SH418/AR, and SH450/AR were generated in a similar way (Ichihara et al, 2017) and generously provided by Dr. Christine M. Lovly (Vanderbilt University Medical Center and Vanderbilt Ingram Cancer Center, Nashville, TN). PC-9/3 M (EGFR 19del/T790M/C797S triple mutations) was generated by engineering the PC-9 cell line using a lentiviral expression construct harboring EGFR exon 19del/T790M/C797S (Niederst et al, 2015) and provided by Dr. Aaron N. Hata (Harvard Medical School, Boston, MA). Murine EGFRm NSCLC cell lines, 19.1 Del, 19,2 Del and L860R (Kleczko et al, 2023), were generously provided by Dr. Raphael A. Nemenoff (University of Colorado Anschutz Medical Campus, Aurora, CO). Cell lines that stably overexpress the *PPARGC1B* gene were established by the infection of lentiviruses carrying a human *PPARGC1B* gene, followed by puromycin selection. *PPARGC1B* lentiviral plasmid (catalog LC137300) and matching vector pLenti-III-CMV were purchased from Applied Biological Materials Inc. (Richmond, BC) and used as instructed by the manufacturer. PC-9, HCC827, and their derived osimertinib-resistant cell lines were authenticated by short tandem repeat (STR) profiling at the Arizona Genetics Core (Tucson, AZ). All cell lines were routinely tested for mycoplasma contamination. All cell lines were cultured in RPMI 1640 medium supplemented with at 5% FBS at 37 °C in 5% CO_2_ humidified air.

### MitoSOX™ Red staining

Cells were seeded in VWR confocal dishes at appropriate densities and were exposed to the tested drugs on the second day. After the indicated times, the cells were washed with HBSS and then incubated with 5 µM MitoSOX working solution (diluted with serum-free medium) for 10 min at 37 °C. Cells were then washed with HBSS three times to remove excess dye and incubated with Hoechst 33342 for 10 min. After washing with HBSS three times, the solution was replaced with phenol-red–free, serum-free medium. Images were collected using a Leica TCS SP8 confocal microscope.

### MitoTracker™ Green FM staining

Cells were plated in VWR confocal dishes at suitable densities and allowed to adhere overnight. On the second day, the cells were exposed to the indicated drugs for specified durations. Following treatment, cells were washed with HBSS and incubated with 100 nM MitoTracker™ Green FM (diluted in serum-free medium) for 30 min at 37 °C. After staining, cells were washed three times with HBSS and subsequently counterstained with Hoechst 33342 for 10 min. Excess dye was removed by three additional washes with HBSS, and cells were maintained in phenol-red–free, serum-free medium before imaging. Confocal images were obtained using a Leica TCS SP8 microscope.

### Mitochondrial ToxGlo™ assay

Cellular ATP levels were determined using the Mitochondrial ToxGlo™ Assay (Promega; Madison, WI) following the manufacturer’s protocol. Briefly, cells were seeded in 96-well white opaque plates and incubated overnight. After drug treatment for the indicated time, ATP detection reagent was added directly to the wells, and luminescence was measured using a plate reader. Relative ATP levels were normalized to vehicle-treated controls.

### Western blot analysis

Cells were washed with PBS and lysed in lysis buffer containing 50 mM Tris-HCl (pH 8.0), 150 mM NaCl, 0.1% SDS, 1% Nonidet P-40, 1 mM phenylmethylsulfonyl fluoride, 5 μg/ml aprotinin, and 5 μg/ml leupeptin. Whole-cell protein lysates were collected and clarified by centrifugation, and protein concentrations were determined using a BCA protein assay. Equal amounts of protein lysates were separated by SDS-PAGE and transferred onto PVDF membranes. Membranes were blocked with 5% non-fat milk in TBST and incubated with primary antibodies overnight at 4 °C. Primary antibodies were diluted at 1:1000 in 5% BSA in TBST containing sodium azide. Membranes were then incubated with HRP-conjugated secondary antibodies diluted at 1:3000 in 5% non-fat milk in TBST at room temperature. Protein signals were detected using enhanced chemiluminescence. α-Tubulin or GAPDH was used as an internal loading control. Membranes were not stripped and reprobed. When needed, membranes were cut according to molecular weight to detect different target proteins and loading controls. One sample from each treatment was loaded on SDS-PAGE. The results were confirmed in multiple cell lines and/or with biological replications. Protein bands were quantified with NIH ImageJ software.

### Apoptosis assays

Apoptosis was assessed using the Annexin V/7-AAD apoptosis detection kit (BD Biosciences, San Jose, CA) according to the manufacturer’s instructions. In addition, apoptotic cell death was confirmed by detecting the cleavage of apoptosis-related proteins through western blotting.

### Cell survival assay

Cells were seeded into 96-well plates at appropriate densities and then treated with the indicated drugs either alone or in combination on the second day for 3 days. Cell numbers were determined using the sulforhodamine B (SRB) assay as previously reported (Sun et al, 1997). The Combination Index (CI) for drug interactions was calculated using CompuSyn software (ComboSyn, Inc., Paramus, NJ).

### Colony formation assay

PC-9/AR and HCC827AR cells seeded in 12-well plates at a density of 200 cells per well for about 24 h were exposed to the indicated drugs. Medium containing the same drugs was refreshed every 3 days. Following 10 days of incubation, the medium was removed to fix and stain the cell colonies with 2% crystal violet in ethanol. Colonies were then photographed and quantified.

### RNA extraction and qRT-PCR

Total RNA was extracted from cells using the RNA extraction kit (Qiagen; Germantown, MD) according to the manufacturer’s instructions. RNA concentrations were determined with a NanoDrop spectrophotometer (Thermo Fisher Scientific). Reverse transcription was performed using the RevertAid First Strand cDNA Synthesis Kit (Thermo Fisher Scientific). Quantitative real-time PCR (qRT-PCR) was carried out on the QuantStudio 3 and QuantStudio 5 systems (Thermo Fisher Scientific) with the following cycling conditions: 95 °C for 15 s, followed by 40 cycles of 95 °C for 5 s and 60 °C for 30 s. The primer sequences were as follows: *PPARGC1B*, forward 5′-TGAGCAGACCTTGACAGTGGAG -3′ and reverse 5′-GACTATGCTTGATGTCTGGTTTGA-3′; *GAPDH* (endogenous control), forward 5′-GTCTCCTCTGACTTCAACAGCG-3′ and reverse 5′-ACCACCCTGTTGCTGTAGCCAA-3.

### Immunofluorescence staining

Cells grown on chamber slides were fixed with 4% paraformaldehyde for 10 min and washed three times with PBS. The slides were then blocked with blocking buffer containing 5% BSA and 0.2% Triton X-100 for 30 min at room temperature, followed by overnight incubation at 4 °C with primary antibody against PGC1β (1:100 dilution). After washing three times with PBS, slides were incubated with the donkey anti-rabbit IgG (H + L) highly cross-adsorbed secondary antibody, Alexa Fluor™ 488 (1:200 dilution), for 1 h at room temperature. Nuclei were counterstained with DAPI-containing mounting medium. Images were acquired using a confocal microscope (Leica TCS SP8).

### Human NSCLC tissues

A total of 21 paired tumor tissues from patients with EGFRm NSCLC were collected before treatment (baseline) and after disease progression following EGFR-TKI therapy in the Second Xiangya Hospital, Central South University (Changsha, Hunan, China) and Henan Cancer Hospital (Zhengzhou, Henan, China) under protocols approved by the Institutional Review Boards (IRB) of the Second Xiangya Hospital (IRB2019-009), Henan Cancer Hospital (IRB2019-067), and Emory University (IRB00104138). Among these cases, 12 paired samples were obtained from patients treated with first-generation EGFR-TKIs, and 9 paired samples were obtained from patients treated with osimertinib. Written informed consent was obtained from all subjects before sample collection. All experiments involving human tissues conformed to the principles set out in the World Medical Association Declaration of Helsinki and the Department of Health and Human Services Belmont Report. Tissue samples were processed and stained at the Second Xiangya Hospital using immunohistochemistry as described below.

### Immunohistochemistry (IHC) staining

Xenograft tumor tissue sections were dewaxed in xylene and rehydrated through a graded alcohol series. The slides were then incubated with 3% (v/v) hydrogen peroxide for 10 min and blocked with 10% BSA in PBS for 1 h at room temperature. After blocking, the tissue slides were incubated with primary antibodies (cleaved PARP at 1:50 dilution and Bim at 1:100 dilution) at 4 °C in a humidified chamber overnight. Detection was performed using the ImmPRESS® Horse Anti-Rabbit IgG Polymer Kit (Vector Laboratories) as previously described (Chen et al, 2022). Human NSCLC tissues were stained using the EnVision™ + Dual Link System-HRP Kit (Dako; Carpinteria, CA) as described previously (Zhang et al, 2021). PCG1β antibody was diluted at 1:100. The staining data were finally quantified as a weight index (WI) that represents % positive stain in tumor multiplied by intensity score (0–3) as described previously (Elrod et al, 2010).

### Transient transfection and luciferase reporter assay

Cells seeded in 24-well plates overnight were transiently transfected with a *PPARGC1B* promoter-driven luciferase reporter plasmid together with the pCH110 plasmid that encodes β-galactosidase using Fugene 6 reagent (Roche Applied Science; Indianapolis, IN) following the manufacturer’s instructions. After 24 h, the cells were treated with either DMSO or osimertinib for 16 h and then harvested in cell-lysis buffer for measurement of the luciferase activity with a Luciferase Assay kit (Promega) using a Sirius Luminometer (Berthold Detection Systems, Huntsville, AL). The luciferase readings were normalized to β-galactosidase activity measured as previously described (Pfahl et al, 1990).

### Animal xenograft and treatments

All animal experiments were approved by the Institutional Animal Care and Use Committee (IACUC) of Emory University (PROTO201700718). Female 4-week-old outbred athymic nude mice carrying the homozygous Foxn1^nu mutation (J; The Jackson Laboratory, Bar Harbor, ME, USA; stock no. 007850) were housed in a specific pathogen-free facility under controlled temperature and humidity with a 12-h light/dark cycle. The tested cells (3 × 10^6^ per mouse) were subcutaneously injected into the flanks of 4-week-old nu/nu nude mice (The Jackson Laboratory; Bar Harbor, ME). When the average tumor sizes reached around 80 mm^3^, mice were randomized into groups with equal average tumor sizes and body weights. The following treatments were administered daily: vehicle, osimertinib (5 mg/kg, o.g.), CPI-613 (25 mg/kg, i.p.), and the combination of osimertinib and CPI-613. Tumor volume was measured using calipers every 2 or 3 days and calculated by V = π (length × width^2^)/6. Body weight was also measured every 2 or 3 days. At the endpoint, mice were sacrificed using CO₂, and tumors were excised, weighed, and fixed in formalin for subsequent analyses.

### Gene set enrichment analysis (GSEA)

GSEA was performed to assess coordinated enrichment of a predefined mitochondrial gene set using the R package FGSEA (Korotkevich et al, 2021). A pre-ranked gene list was used as input, where genes were ordered by a signed statistic from DESeq2 in differential expression analysis comparing cells treated with osimertinib versus DMSO control. The mitochondrial gene set was obtained from a public database, Human MitoCarta3.0 (Rath et al, 2021), and comprised 473 genes after prescreening based on the database-provided confidence scores. After matching gene identifiers to the ranked dataset, 67 genes were retained and included in the enrichment analysis. The enrichment score was calculated as a weighted running-sum statistic across the ranked gene list. Briefly, the algorithm iteratively traverses all genes in rank order, increasing the running score when a gene belongs to the gene set (weighted by the absolute value of its ranking statistic) and decreasing the score for genes not in the set by a constant penalty. This produces a running enrichment score across all positions in the ranked list. Statistical significance, i.e., *P* values, for the enrichment of the mitochondrial gene set was evaluated using permutation-based procedures implemented in FGSEA. Enrichment curves were visualized using the plotEnrichmentData function in FGSEA, where the *x* axis represents gene rank in the ordered list and the *y* axis represents the running enrichment score. Tick marks indicate the positions of genes from the mitochondrial gene set within the ranked list.

### Statistical analysis

Statistical differences were evaluated using two-sided unpaired or paired Student’s *t* test for two groups or one-way ANOVA test for multiple groups. Data are presented as means ± SDs or SEs, as indicated. All statistical analyses were performed with Graphpad Prism 10 software. *P* value < 0.05 was considered statistically significant. Sample sizes were included in each figure legend. Clinical IHC staining was evaluated in a blinded manner. No blinding was performed during other experimental data collection and analyses. No data was excluded.

## Supplementary information

Appendix

Peer Review File

Source data Fig. 1

Source data Fig. 2

Source data Fig. 3

Source data Fig. 4

Source data Fig. 5

Source data Fig. 6

Source data Fig. 7

Source data Fig. 8

Source data Fig. 9

Expanded View Figures

## Data Availability

This study contains no data deposited in external repositories. The source data of this paper are collected in the following database record: biostudies:S-SCDT-10_1038-S44321-026-00493-7.
