# Supplementary material for: Inhibition of PGC1β-dependent mitochondrial biogenesis enhances EGFR-targeted therapy in lung cancer
Source: EMBO Mol Med. 2026 Jul 30;18(9):3540–69. doi: 10.1038/s44321-026-00493-7 (PMC13562578; doi:10.1038/s44321-026-00493-7)
Supplement: Supplementary file 1 [file 44321_2026_493_MOESM12_ESM.pdf]

Expanded View Figures

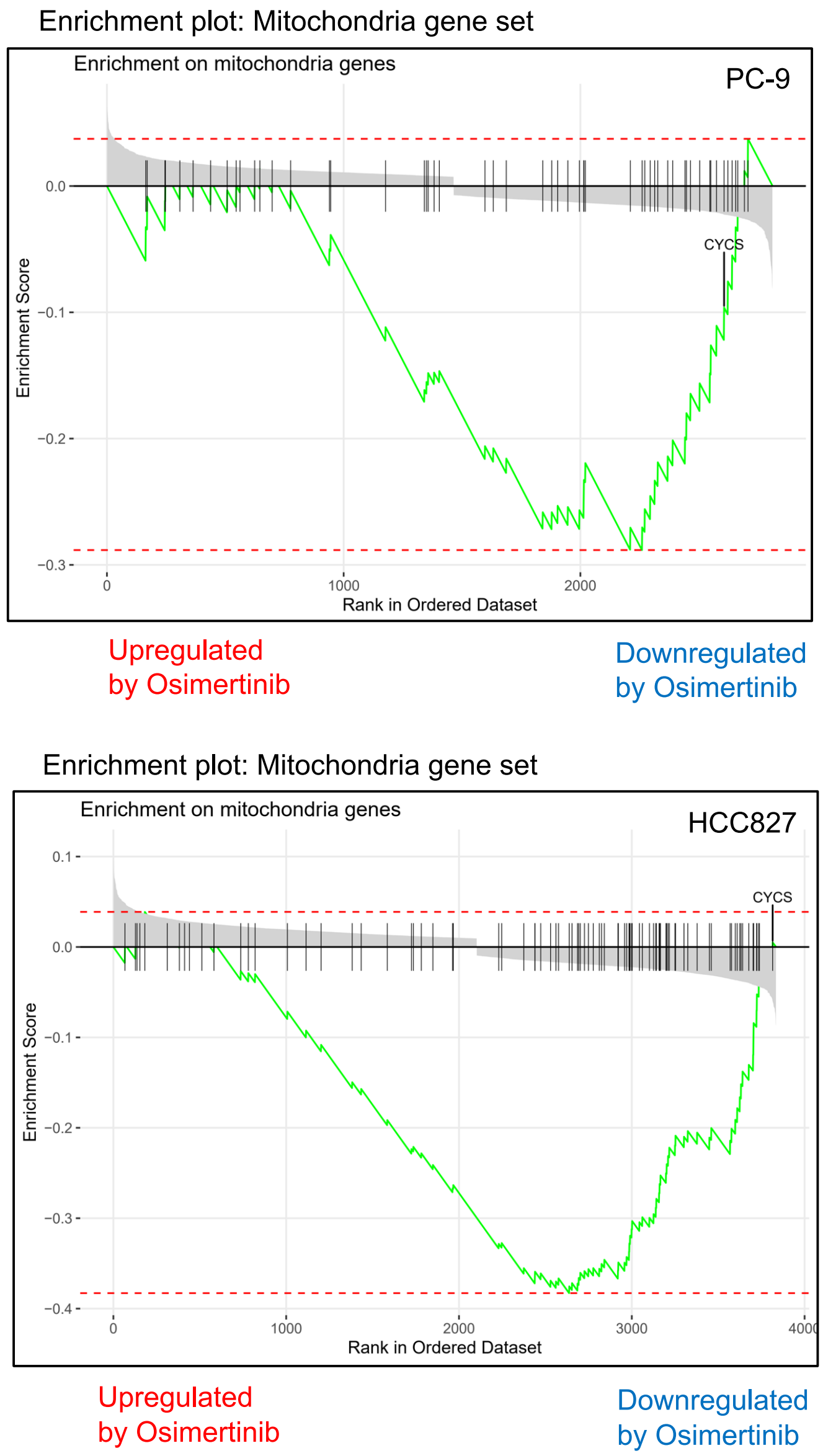

Figure EV1. GSEAs showing negative enrichment of mitochondrial gene set in EGFRm NSCLC cells treated with osimertinib compared with DMSO control.

Enrichment plot showing the running enrichment score (ES) for the mitochondrial gene set (Human MitoCarta3.0) across a ranked list of genes in HCC827 and PC9 cells treated with osimertinib versus DMSO. The *x* axis represents gene rank in the ordered dataset, and the *y* axis shows the running enrichment score. Vertical ticks indicate positions of mitochondrial genes. The negative ES indicates enrichment of mitochondrial genes toward the bottom of the ranked list, consistent with downregulation upon treatment.

 © The Author(s)

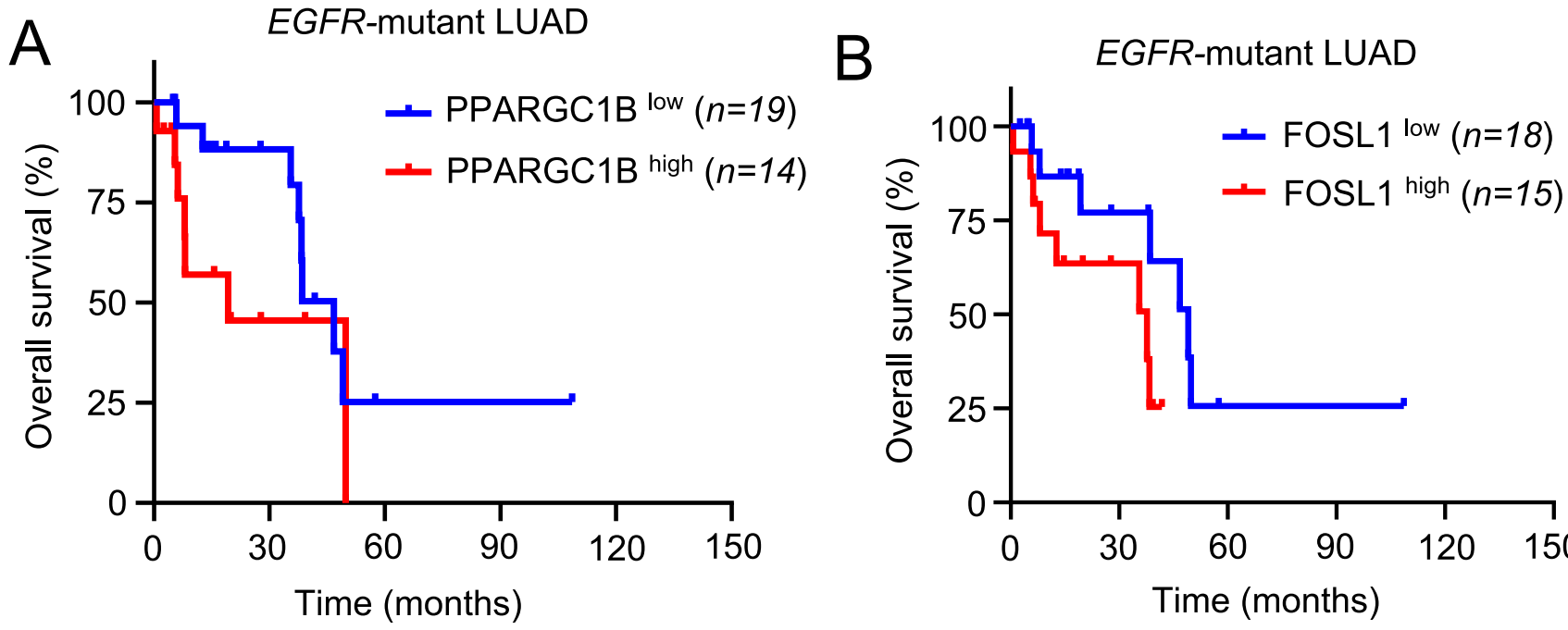

PPARGC1B	High	Low	*P* value
OS (months)	19.25	46.6	0.042

FOSL1	High	Low	*P* value
OS (months)	37.6	49.0	0.06

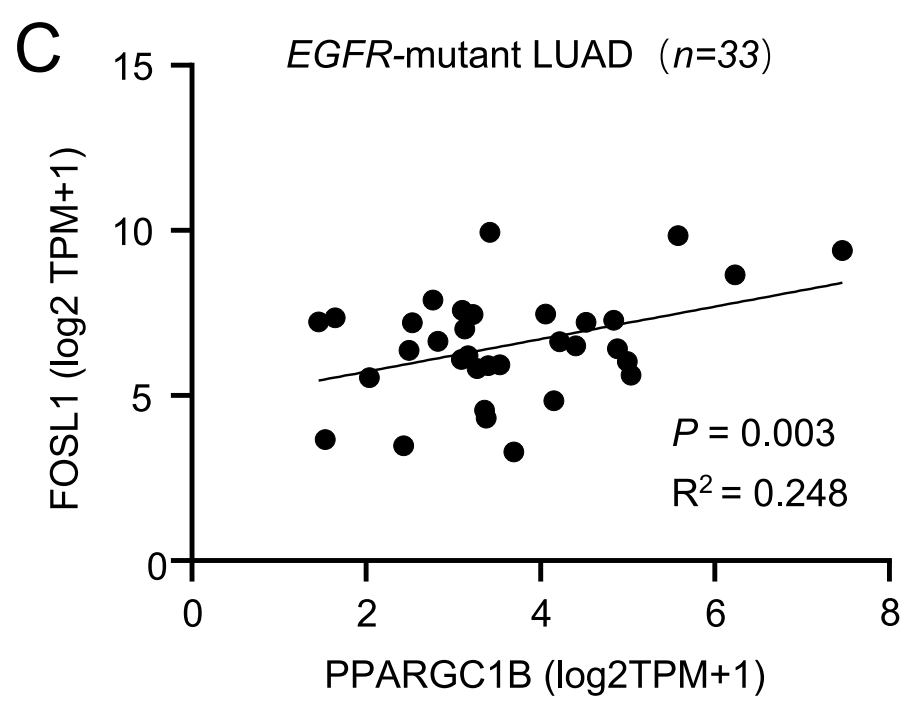

Figure EV2. TCGA analysis of *PPARGC1B* and *FOSL1* expression in EGFRm lung adenocarcinoma (LUAD) tissues and their impacts on patient survival.

(**A**, **B**) High versus low expression of PPARGC1B or FOSL1 in 33 tumors was stratified using the median expression level as the cutoff. Overall survival was analyzed by Kaplan–Meier survival analysis, and P values were determined by the log-rank test. (**C**) PPARGC1B and FOSL1 mRNA expression data from the 33 EGFRm tumors in the TCGA database were extracted, and their correlation was then analyzed. TPM, transcripts per million.

© The Author(s)

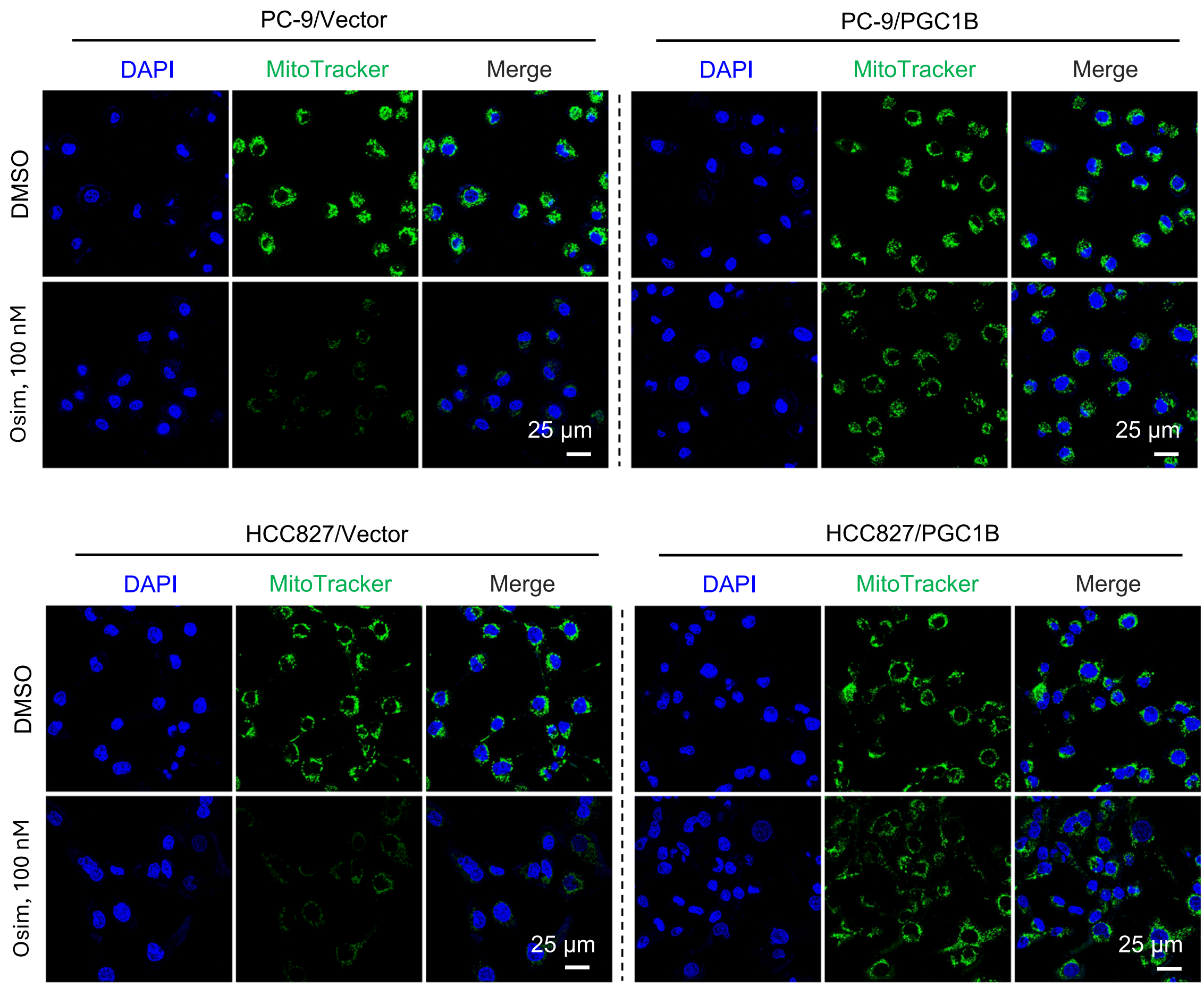

Figure EV3. Impact of enforced expression of ectopic *PPARGC1B* (*PGC1B*) in EGFRm NSCLC cell lines on osimertinib-induced mitochondrial alteration.

The indicated cell lines were treated with 100 nM osimertinib for 24 h and then stained with MitoTracker green.

© The Author(s)

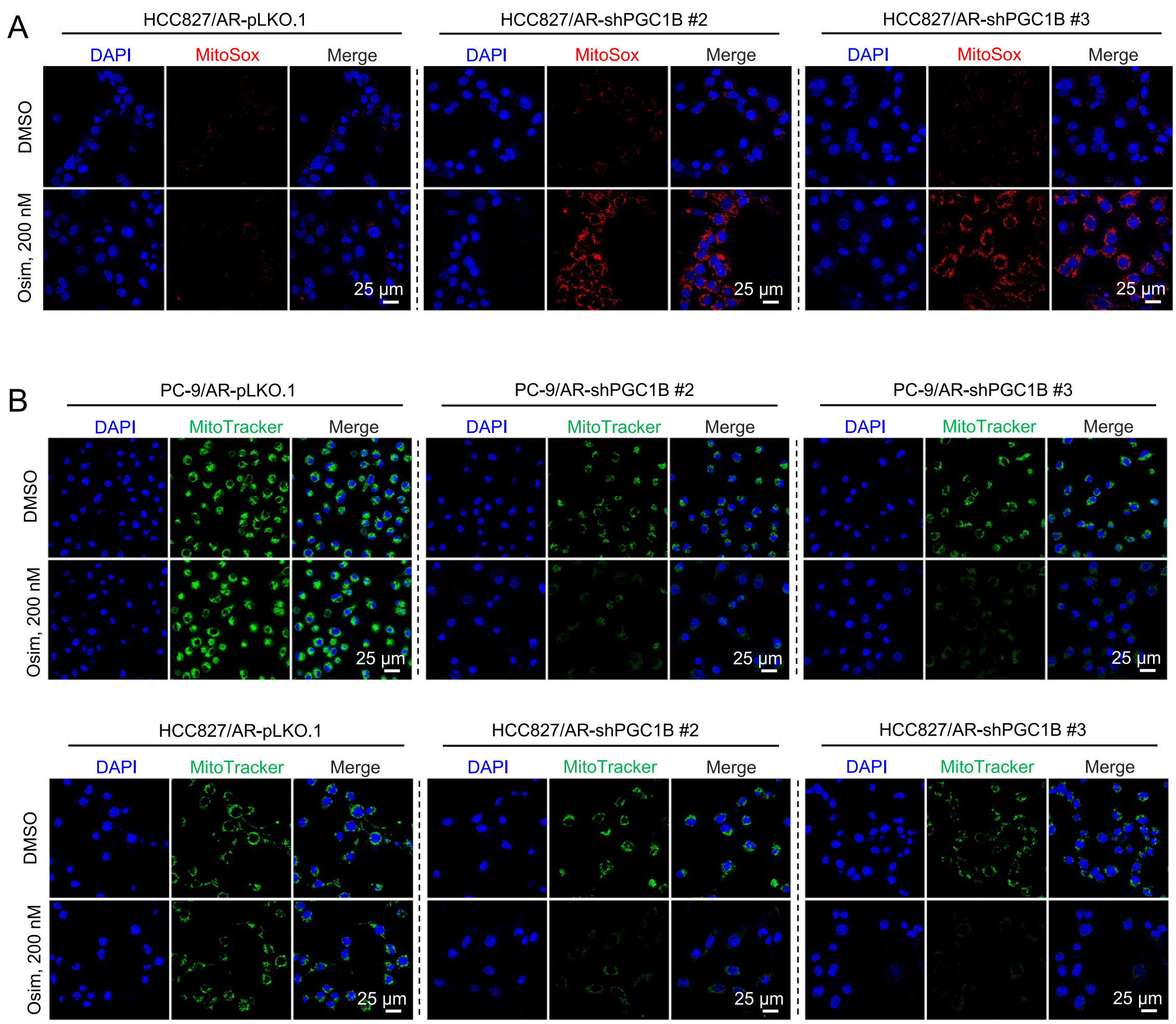

Figure EV4. *PPARGC1B* (*PGC1B*) knockdown in osimertinib-resistant cells restores osimertinib's ability to inhibit mitochondrial biogenesis.

The indicated cell lines were exposed to 200 nM osimertinib (Osim) for 24 h and then stained with MitoSox Red dye for mitochondrial ROS (**A**) or MitoTracker Green FM dye for mitochondrial mass (**B**).

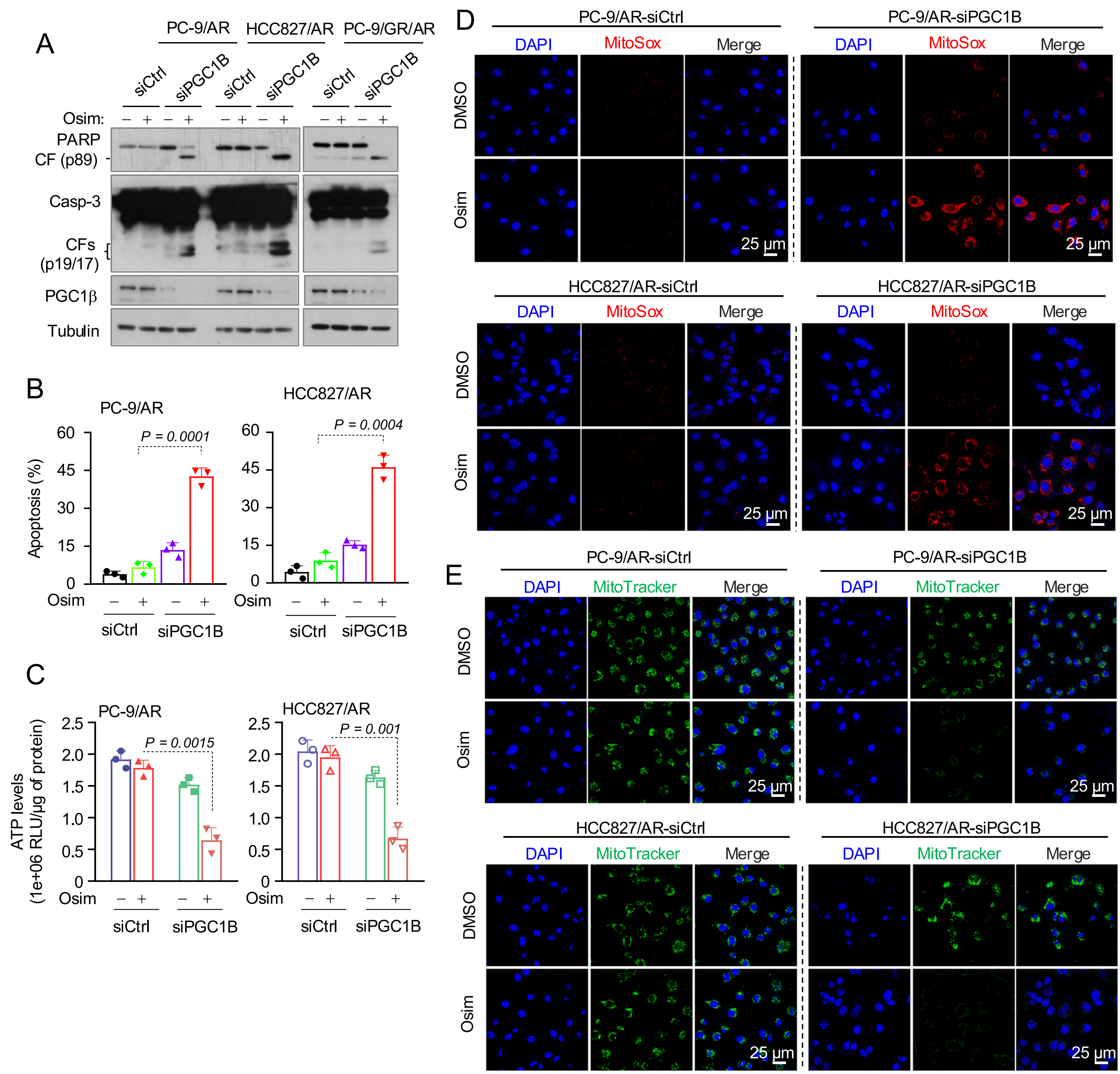

Figure EV5. *PPARGC1B* (*PGC1B*) knockdown with siRNA in osimertinib-resistant cells sensitizes the cells to undergo apoptosis with suppression of mitochondrial biogenesis.

The indicated cell lines were transfected with control or *PPARGC1B* (*PGC1B*) siRNA for 48 h, followed by treatment with 200 nM osimertinib for 48 h (**A**, **B**) or 24 h (**C**–**E**). The proteins of interest were detected with Western blotting (**A**). Annexin V-positive cells were analyzed with flow cytometry (**B**). ATP levels were measured using the Mitochondrial ToxGlo™ Assay (**C**). Mitochondrial ROS were detected by MitoSOX™ Red staining (**D**). Mitochondrial mass was detected by MitoTracker® Green FM staining (**E**). The data in (**B**, **C**) are means ± SDs of triplicate determinations. Statistical differences between the two given groups were evaluated with a two-sided unpaired Student's *t* test.

© The Author(s)

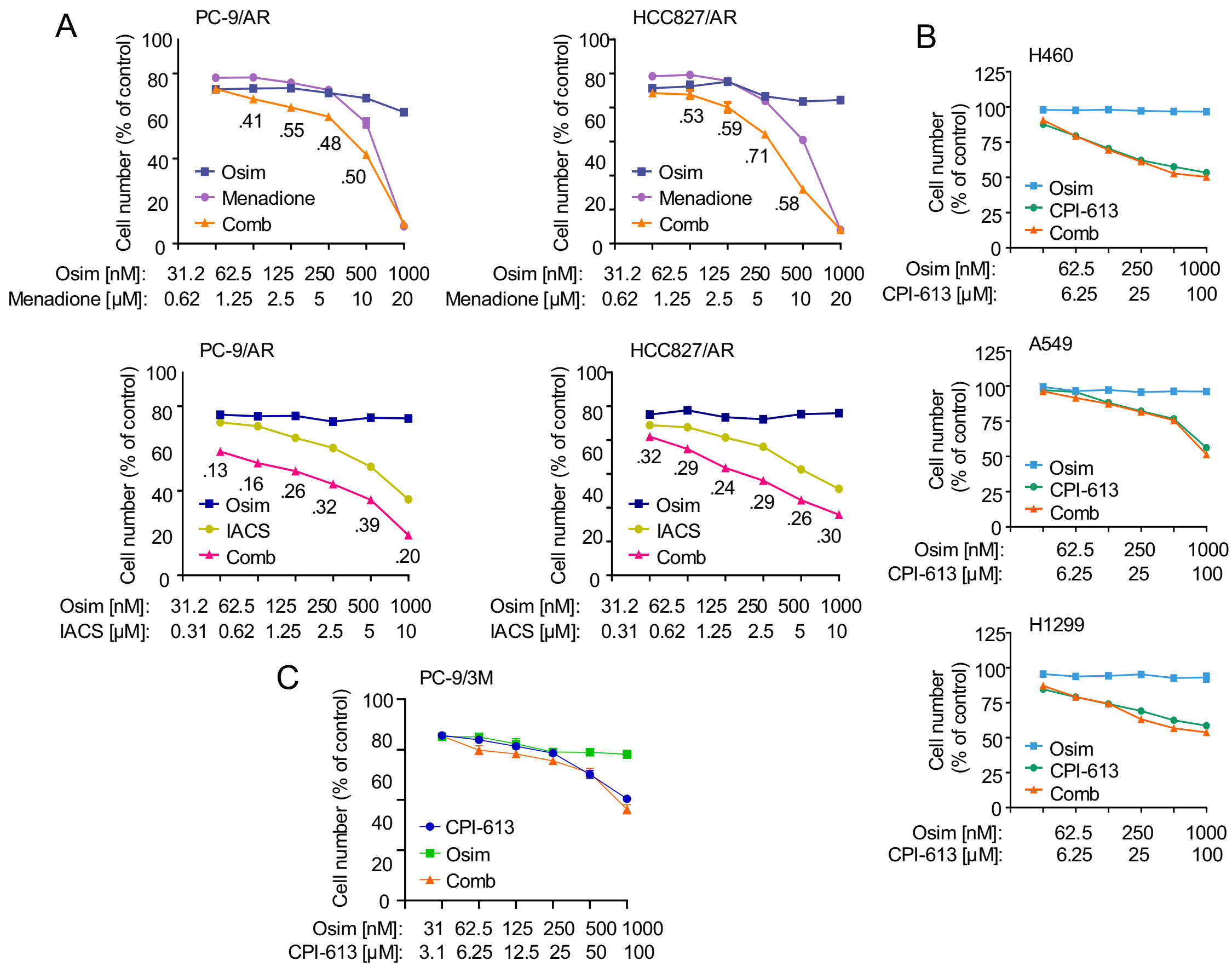

Figure EV6. Effects of osimertinib combined with a mitochondria-targeting agent on the survival of EGFRm NSCLC cell lines with acquired resistance to osimertinib and NSCLC cell lines with WT EGFR.

The indicated cell lines with mutant (**A**, **C**) or WT (**B**) EGFR seeded in 96-well plates were treated with different concentrations of the tested agents alone or in combination for 3 days. Cell numbers were then estimated with the SRB assay. The data are means ± SDs of four replicate determinations. IACS, IACS-010759.

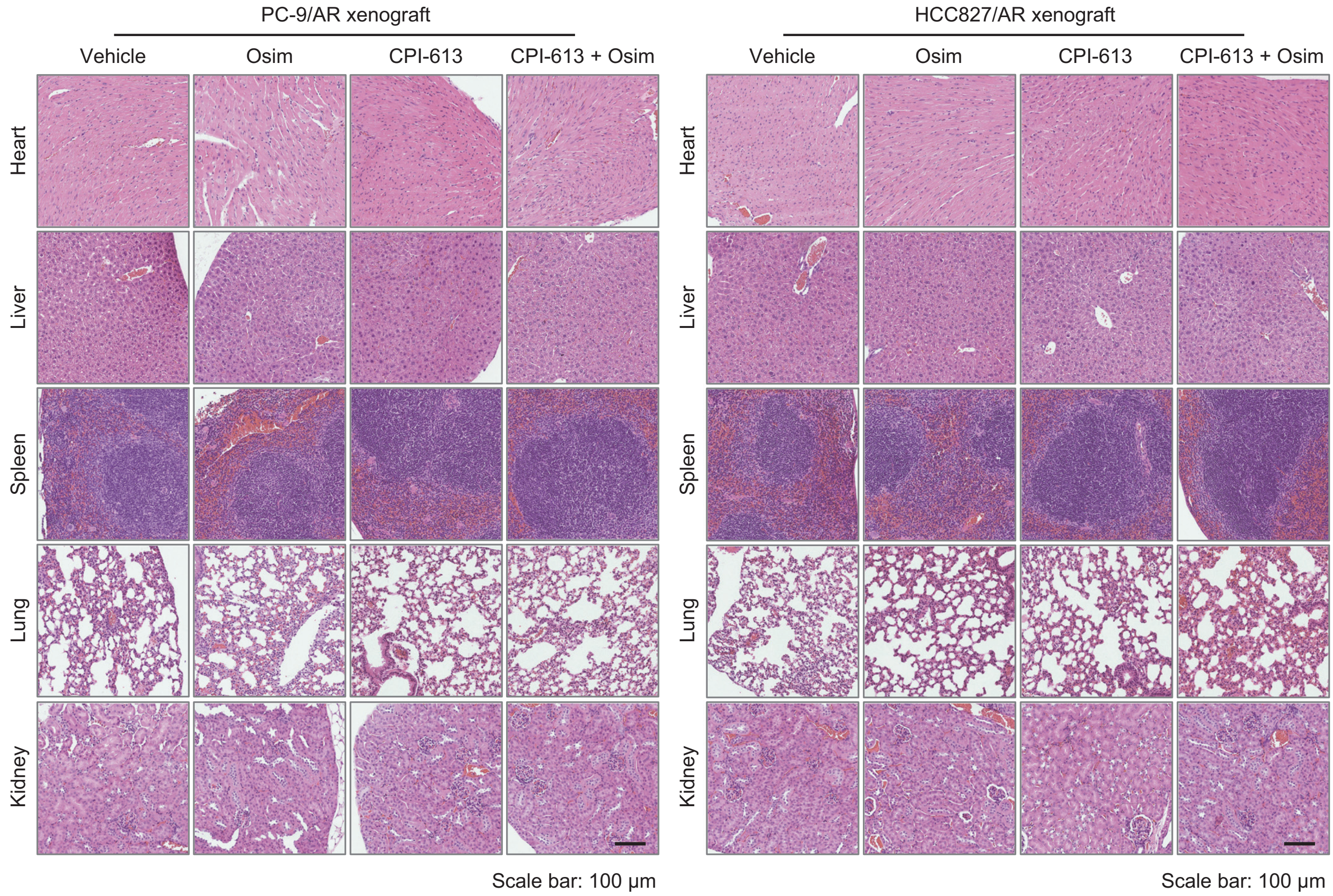

Figure EV7. The combination of osimertinib and CPI-613 does not show harmful effects on the major organs of mice.

The treatments were the same as described in Fig. 9. The morphologies of the tested organs as indicated were evaluated with H&E staining.

 © The Author(s)

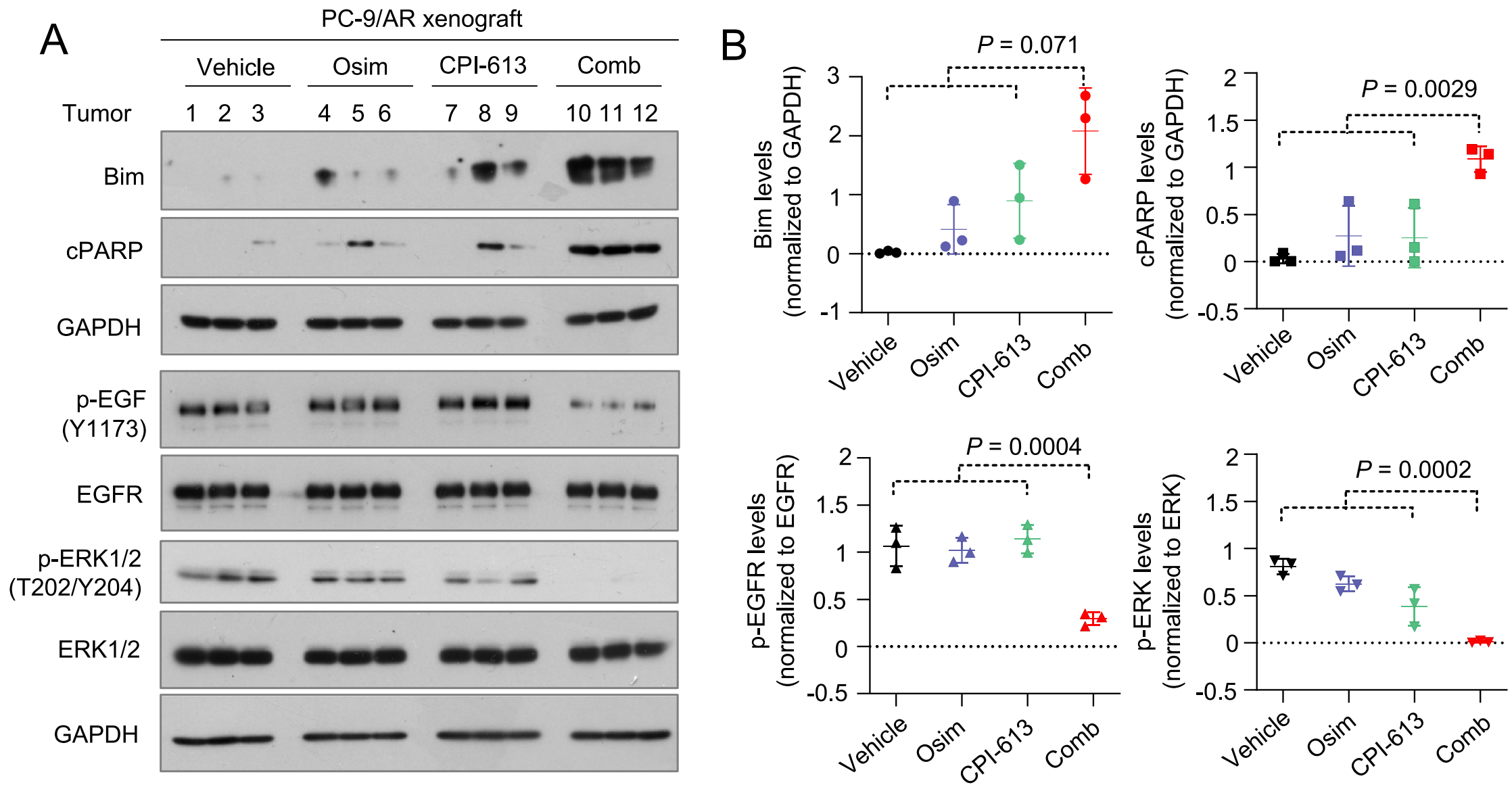

Figure EV8. Detection of protein markers in PC-9/AR tumor tissues using western blotting.

The tumor treatments are the same as described in Fig. 9. Proteins of interest were detected with western blotting (**A**). Protein quantification was conducted using ImageJ software (**B**). The data is presented as means ± SDs generated from three different tumor samples. Statistical differences among the treatment groups were evaluated with a one-way ANOVA test.

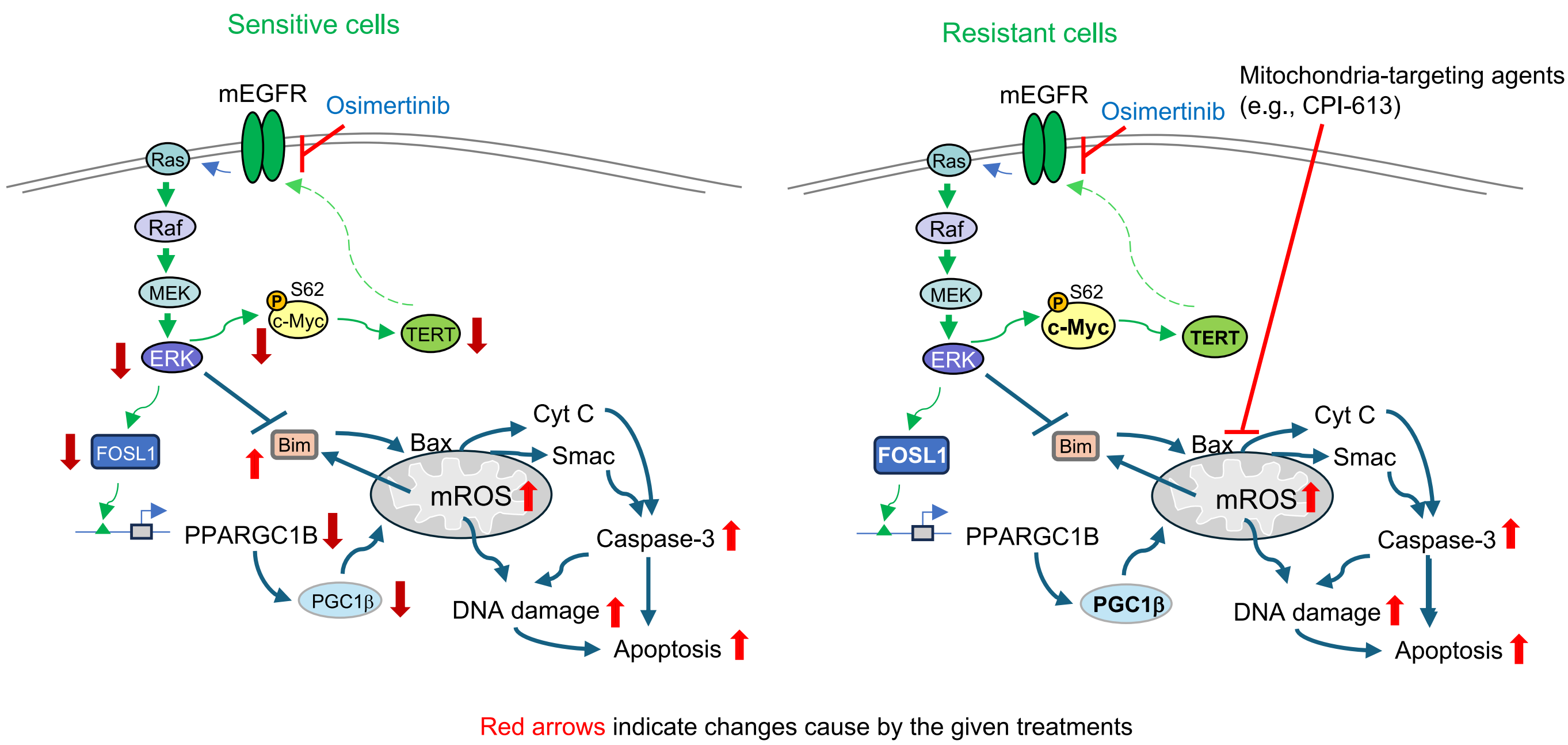

Figure EV9. Schemas for downregulation of FOSL1-dependent regulation of PPARGC1B expression, suppression of mitochondrial biogenesis, such as mitochondrial ROS (mROS) generation, and induction of Bim-dependent apoptosis in osimertinib-sensitive cells (left panel) and for restoration of this mechanism through co-targeting mitochondria using a mitochondria-targeting agent such as CPI-613 in osimertinib-resistant cells (right panel), respectively.

Cyt C cytochrome C.

 © The Author(s)
