# Supplementary material for: Inhibition of PGC1β-dependent mitochondrial biogenesis enhances EGFR-targeted therapy in lung cancer
Source: EMBO Mol Med. 2026 Jul 30;18(9):3540–69. doi: 10.1038/s44321-026-00493-7 (PMC13562578; doi:10.1038/s44321-026-00493-7)
Supplement: Supplementary file 2 [file 44321_2026_493_MOESM1_ESM.pdf]

Table of contents

Appendix Figure S1	Page 2
Appendix Figure S2	Page 3
Appendix Figure S3	Page 4
Appendix Table S1	Page 5
Appendix Table S2	Page 6

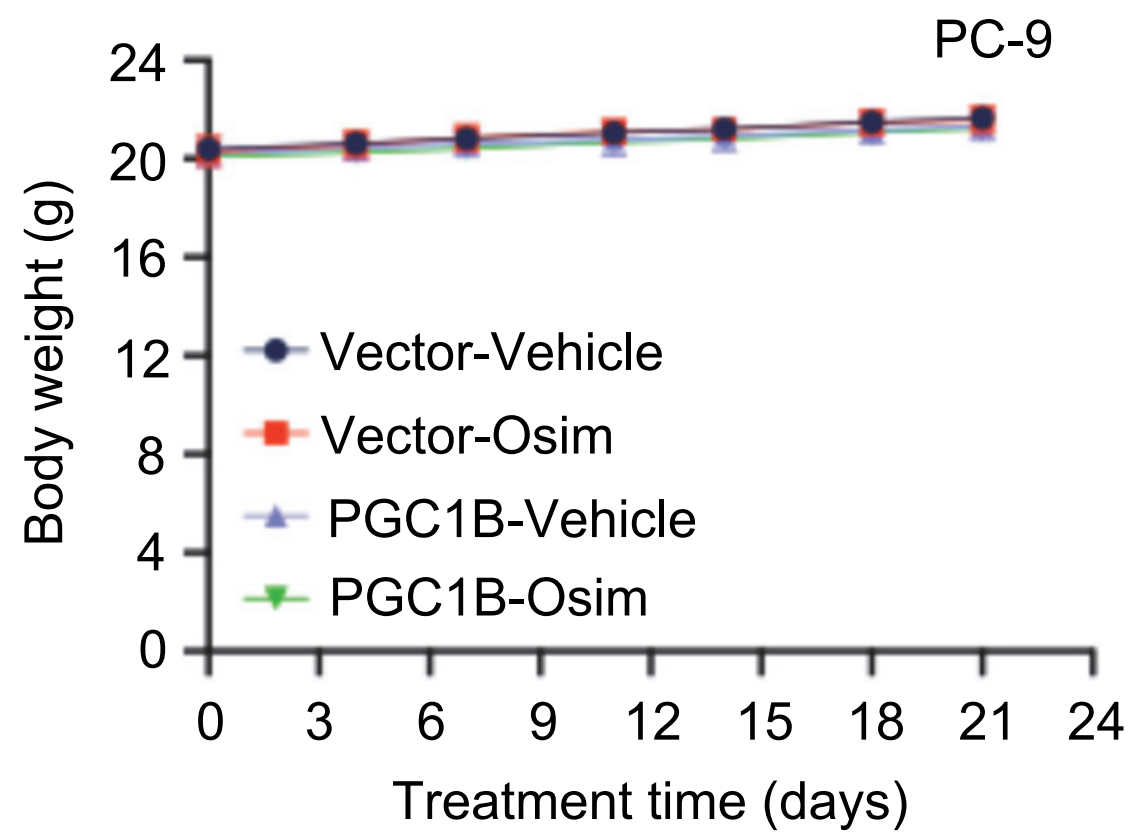

Appendix Figure S1. Osimertinib treatment of PC-9 tumors with ectopic *PPARGC1B* (*PGC1B*) expression does not affect mouse body weights. The experimental conditions were the same as described in Figs. 4K-N. The data are means ± SEs (n = 6).

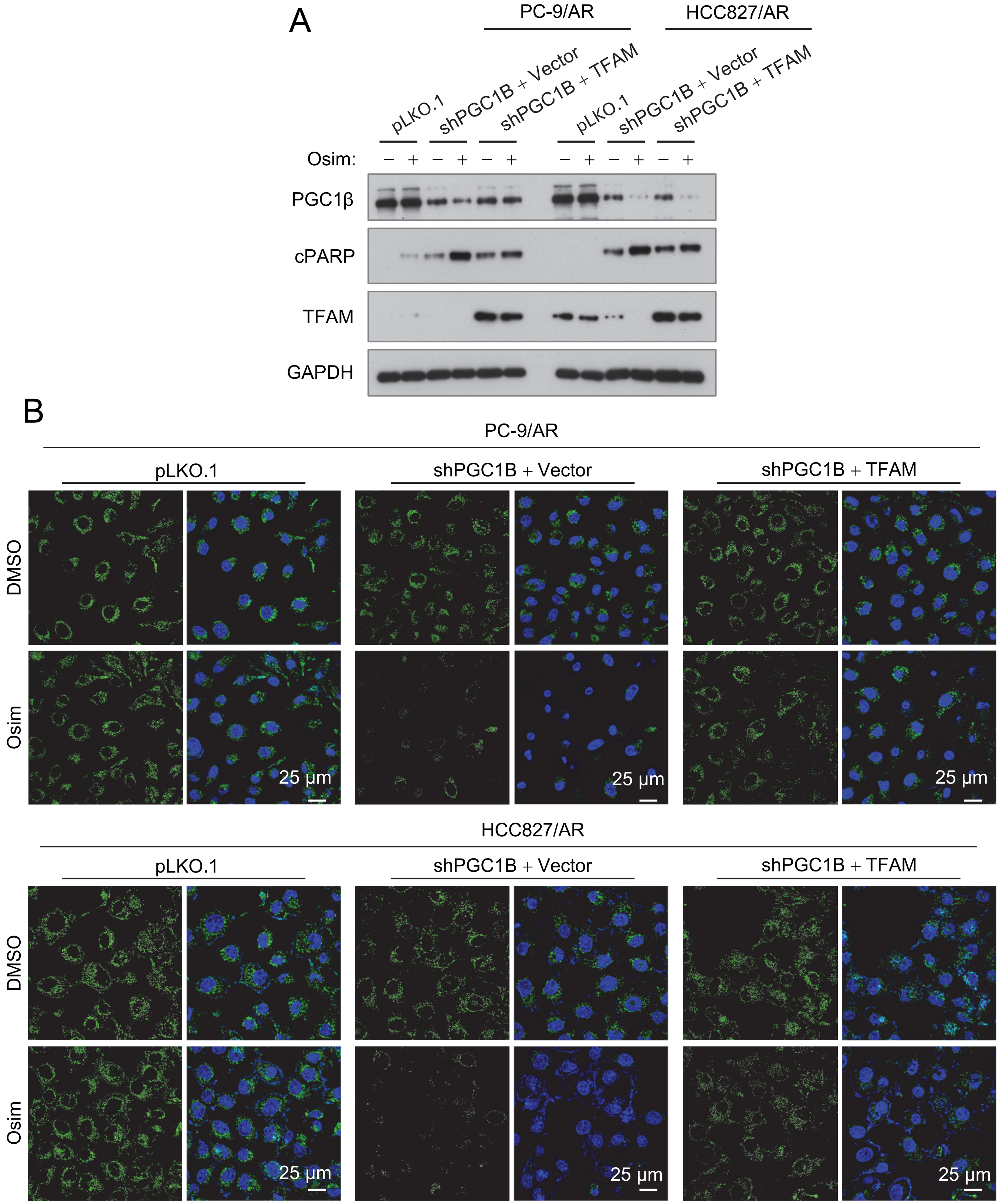

Appendix Figure S2. Restoration of mitochondrial biogenesis via TFAM rescues PGC1β knockdown–induced sensitization to osimertinib. *A*, Proteins of interest were detected with Western blotting in the indicated cell lines treated with DMSO or 200 nM osimertinib (Osim) for 48 h. *B*, MitoTracker staining was visualized using confocal microscopy in the indicated cell lines treated with DMSO or 200 nM osimertinib for 48 h. Nuclei were counterstained with DAPI. Scale bars, 25 μm.

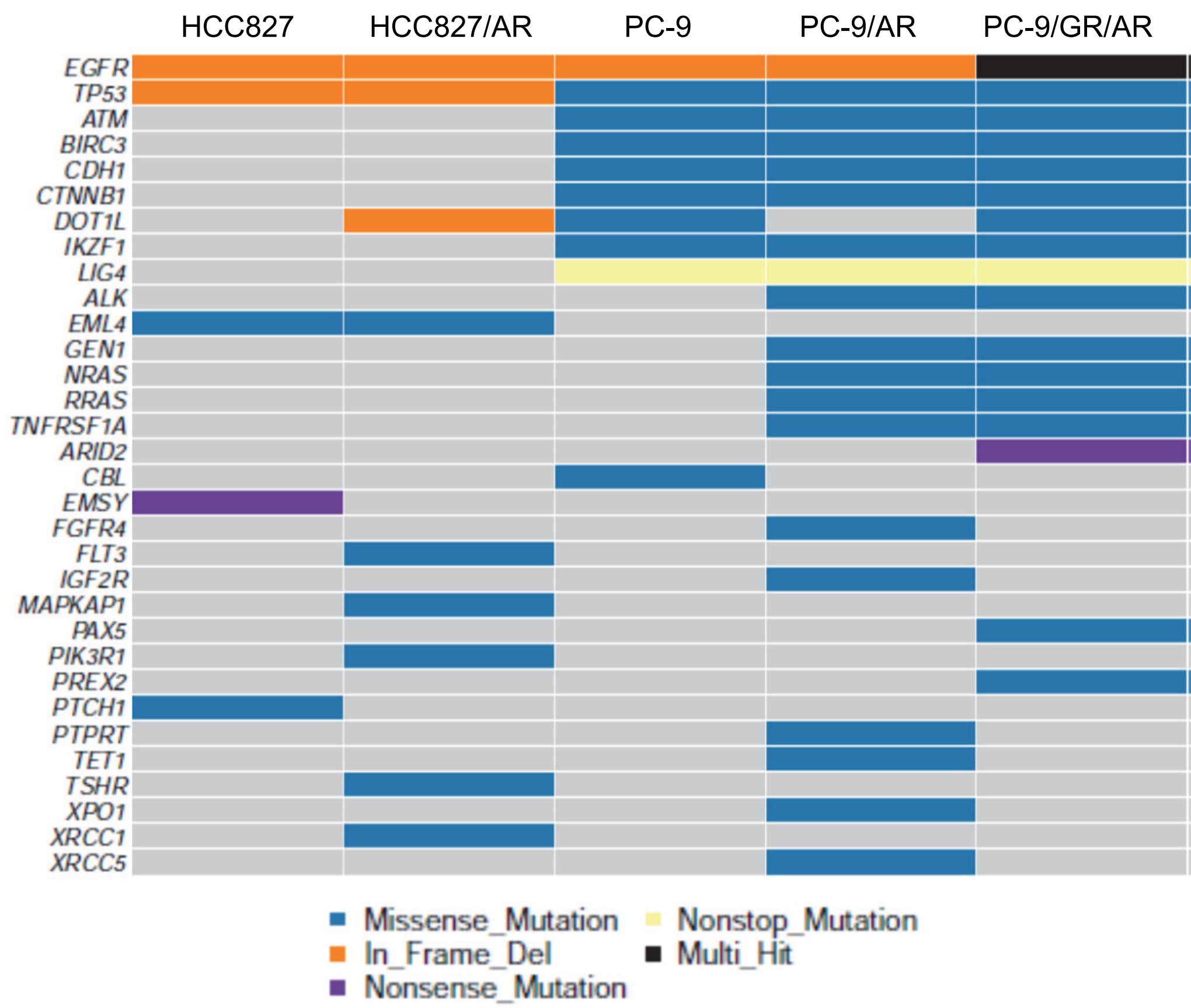

Appendix Figure S3. Detection of novel gene mutations in EGFRm NSCLC cell lines with acquired resistance. The gene mutations in the test cell lines were detected with PredicineATLAS 600 cancer gene panel NGS assay.

Appendix Table S1. EGFRm NSCLC cell lines with acquired resistance to osimertinib/AZD9291 (AR) and their possible resistance mechanisms

Resistant cell line	EGFR mutations	Resistance mechanisms	Parental line
First-line resistance			
HCC827/AR	19del	MET amplification	HCC827
PC-9/AR	19del	Unknown	PC-9
SH416/AR	19del	Unknown	SH416
SH450/AR	19del	Unknown	SH450
Second-line resistance			
H1975/AR	L858R, T790M	Unknown	H1975
PC-9/GR/AR	19del, T790M	Unknown	PC-9/GR
PC-9/3M	19del, T790M, C797S	C797S	PC-9

Appendix Tabel S2. Gene mutations and PGC1β staining in baseline and after-relapse tissues in EGFRm NSCLC patients treated with 1st generation EGFR-TKIs or osimertinib

	Baseline		After relapse	
**Patients**	**Gene mutations**	**PGC1β (WI)**	**Gene mutations**	**PGC1β (WI)**
1	EGFR p.E746_A750del	210	EGFR p.E746_A750del、TP53 p.V73Wfs*50、STK11 p.P281Rfs*6、CDKN2A p.W15*、PIK3CA p.H1047R、RNF43 p.G659Vfs*41、NBN p.M83Cfs*9	270
2	EGFR p.E746_S752delinsV	180	EGFR p.E746_S752delinsV	180
3	EGFR p.L858R、TP53 p.F134L	40	EGFR p.L858R、TP53 p.F134L	40
4	EGFR p.L858R	5	EGFR p.L858R、EGFR p.T790M、EGFR p.L792F、PIK3CB p.E1051K、BRAF p.G469A	130
5	EGFR p.E746_A750del	165	EGFR p.E746_A750del	270
7	EGFR p.L861Q	60	EGFR p.L861Q	70
8	EGFR p.L858R	0	EGFR p.L858R	5
10	EGFR p.E746_A750del	70	EGFR p.E746_A750del、TP53 p.E294Sfs*51	180
12	EGFR p.L858R	270	EGFR p.L858R	270
13	EGFR p.L858R、EGFR p.L844V	0	EGFR p.L858R	55
15	EGFR p.E746_A750del、TP53 p.R175H、RECQL4 p.R872Efs*12	10	EGFR p.E746_A750del、RECQL4 p.R872Efs*12、TP53 p.R175H	180
16	EGFR p.L747_K754delinsA	5	EGFR p.L747_K754delinsA	5
18	EGFR p.L858R	5	EGFR p.L858R、SMARCA4 p.E1361	0
21	EGFR p.E746_A750del、EGFR p.T790M	5	EGFR p.T790M、EGFR p.E746_A750del	5
22	EGFR p.E746_A750del	0	EGFR p.E746_A750del、TP53 c.376-4_379delACAGTACT、RB1 p.A17Pfs*3	0
23	EGFR p.E746_A750del、EGFR p.T790M	0	NF2 p.R198*、EGFR p.T790M、EGFR p.E746_A750del	5
24	EGFR p.E746_A750del、EGFR p.T790M	0	EGFR p.T790M、EGFR p.E746_A750del	0
25	EGFR p.L858R、EGFR p.T790M	0	EGFR p.L858R、EGFR p.T790M	240
29	EGFR p.L747_T751delinsP、BRCA2 p.K2729N、FANCA p.D694N	5	EGFR p.L747_T751delinsP	180
30	EGFR p.L858R、EGFR p.T790M	0	TP53 p.G245V、EGFR p.L858R、PIK3CA p.E545K、BRCA1 p.E1849	180
32	EGFR p.S768I、EGFR p.G719A	0	EGFR p.S768I、EGFR p.G719A	0
