# Supplementary material for: Inhibition of PGC1β-dependent mitochondrial biogenesis enhances EGFR-targeted therapy in lung cancer
Source: EMBO Mol Med. 2026 Jul 30;18(9):3540–69. doi: 10.1038/s44321-026-00493-7 (PMC13562578; doi:10.1038/s44321-026-00493-7)
Supplement: Supplementary file 3 [file 44321_2026_493_MOESM2_ESM.pdf]

EMBO Molecular Medicine

Inhibition of PGC1β-dependent mitochondrial biogenesis enhances EGFR-targeted therapy in lung cancer

Zhen Chen, Dongsheng Wang, Songqing Fan, Qiming Wang, Yong Huang, Pan Du, Shidong Jia, Suresh Ramalingam, and Shi-Yong Sun

Corresponding author(s): Zhen Chen (zhen.chen@emory.edu) , Shi-Yong Sun (ssun@emory.edu)

Review Timeline:

Submission Date:	25th Dec 25
Editorial Decision:	4th Feb 26
Revision Received:	28th May 26
Editorial Decision:	21st Jun 26
Revision Received:	6th Jul 26
Accepted:	15th Jul 26

Editor: Lise Roth

Transaction Report:

(Note: With the exception of the correction of typographical or spelling errors that could be a source of ambiguity, letters and reports are not edited. Depending on transfer agreements, referee reports obtained elsewhere may or may not be included in this compilation. Referee reports are anonymous unless the Referee chooses to sign their reports.)

4th Feb 2026

Dear Dr. Chen,

Thank you for submitting your manuscript to our editorial office. Please accept our apologies for the delay in responding, which is due to our office's reduced activity over the holiday season and the high volume of manuscripts we have received recently. We have now received feedback from the three reviewers who agreed to evaluate your study. As you will see from the reports below, while they acknowledge the potential interest of the findings, they also highlight several shortcomings and the need for additional mechanistic validation.

Addressing the reviewers' concerns in full will be necessary for further considering the manuscript in our journal, and acceptance of the manuscript will entail a second round of review. EMBO Molecular Medicine encourages a single round of revision only and therefore, acceptance or rejection of the manuscript will depend on the completeness of your responses included in the next, final version of the manuscript. For this reason, and to save you from any frustrations in the end, I would strongly advise against returning an incomplete revision.

If you would like to discuss further the points raised by the referees, I am available to do so via email or video. Let me know if you are interested in this option.

We are expecting your revised manuscript within three months, if you anticipate any delay, please contact us.

When preparing your revised manuscript, please refer to our guidelines: https://link.springer.com/journal/44321/submission-guidelines#cms-Revised-submissions. We perform an initial quality control of all revised manuscripts before re-review; failure to include requested items will delay the evaluation of your revision.

We require:

1) A .docx formatted version of the manuscript text (including legends for main figures, EV figures and tables). Please make sure that the changes are highlighted to be clearly visible.

2) Individual production quality figure files as .eps, .tif, .jpg (one file per figure). For guidance, download the 'Figure Guide PDF': https://media.springernature.com/original/springer-cms/rest/v1/content/27825798/data/v1.

3) A .docx formatted letter INCLUDING the reviewers' reports and your detailed point-by-point responses to their comments. As part of the EMBO Press transparent editorial process, the point-by-point response is part of the Review Process File (RPF), which will be published alongside your paper.

4) A complete author checklist, which you can download from our author guidelines. Please insert information in the checklist that is also reflected in the manuscript. The completed author checklist will also be part of the RPF.

5) Please note that all corresponding authors are required to supply an ORCID ID for their name upon submission of a revised manuscript.

6) It is mandatory to include a 'Data Availability' section after the Materials and Methods. Before submitting your revision, primary datasets produced in this study need to be deposited in an appropriate public database, and the accession numbers and database listed under 'Data Availability'. Please remember to provide a reviewer password if the datasets are not yet public.

In case you have no data that requires deposition in a public database, please state so in this section. Note that the Data Availability Section is restricted to new primary data that are part of this study.

7) For data quantification: please specify the name of the statistical test used to generate error bars and P values, the number (n) of independent experiments (specify technical or biological replicates) underlying each data point and the test used to calculate p-values in each figure legend. The figure legends should contain a basic description of n, P and the test applied. Graphs must include a description of the bars and the error bars (s.d., s.e.m.).

8) At EMBO Press we ask authors to provide source data for the main manuscript figures. You will receive a separate email with instructions for providing source data with your revised manuscript, including how to upload and organize the files.

9) Our journal encourages inclusion of *data citations in the reference list* to directly cite datasets that were re-used and obtained from public databases. Data citations in the article text are distinct from normal bibliographical citations and should directly link to the database records from which the data can be accessed. In the main text, data citations are formatted as follows: "Data ref: Smith et al, 2001" or "Data ref: NCBI Sequence Read Archive PRJNA342805, 2017". In the Reference list,

data citations must be labeled with "[DATASET]". A data reference must provide the database name, accession number/identifiers and a resolvable link to the landing page from which the data can be accessed at the end of the reference.

10) We replaced Supplementary Information with Expanded View (EV) Figures and Tables that are collapsible/expandable online. A maximum of 5 EV Figures can be typeset. EV Figures should be cited as 'Figure EV1, Figure EV2" etc... in the text and their respective legends should be included in the main text after the legends of regular figures.

- For the figures that you do NOT wish to display as Expanded View figures, they should be bundled together with their legends in a single PDF file called *Appendix*, which should start with a short Table of Content. Appendix figures should be referred to in the main text as: "Appendix Figure S1, Appendix Figure S2" etc.

- Additional Tables/Datasets should be labeled and referred to as Table EV1, Dataset EV1, etc. Legends have to be provided in a separate tab in case of .xls files. Alternatively, the legend can be supplied as a separate text file (README) and zipped together with the Table/Dataset file.

11) The paper explained: EMBO Molecular Medicine articles are accompanied by a summary of the articles to emphasize the major findings in the paper and their medical implications for the non-specialist reader. Please provide a draft summary of your article highlighting
- the medical issue you are addressing,
- the results obtained and
- their clinical impact.

This may be edited to ensure that readers understand the significance and context of the research. Please refer to any of our published articles for an example.

12) Author contributions: You will be asked to provide CRediT (Contributor Role Taxonomy) terms in the submission system. These replace a narrative author contribution section in the manuscript.

13) A "Disclosure and Competing Interests Statement" should be provided in the main text.

14) Every published paper includes a 'Synopsis' to further enhance discoverability. Synopses are displayed on the journal webpage and are freely accessible to all readers. They include a short stand first (maximum of 300 characters, including space) as well as 2-5 one-sentences bullet points that summarizes the paper. Please write the bullet points to summarize the key NEW findings. They should be designed to be complementary to the abstract - i.e. not repeat the same text. We encourage inclusion of key acronyms and quantitative information (maximum of 30 words / bullet point). Please use the passive voice. Please attach these in a separate file or send them by email, we will incorporate them accordingly.

Please also suggest a striking image or visual abstract to illustrate your article as a PNG file 550 px wide x 300-600 px high.

15) Include a Reagents and Tools Table as part of the Methods section, which can be downloaded from our author guidelines.

16) As part of the EMBO Publications transparent editorial process initiative (see our Editorial at http://embomolmed.embopress.org/content/2/9/329), EMBO Molecular Medicine will publish online a Review Process File (RPF) to accompany accepted manuscripts.
In the event of acceptance, this file will be published in conjunction with your paper and will include the anonymous referee reports, your point-by-point response and all pertinent correspondence relating to the manuscript. Let us know whether you agree with the publication of the RPF and as here, if you want to remove or not any figures from it prior to publication.
Please note that the Authors checklist will be published at the end of the RPF.

EMBO Molecular Medicine has a "scooping protection" policy, whereby similar findings that are published by others during review or revision are not a criterion for rejection. Should you decide to submit a revised version, I do ask that you get in touch after three months if you have not completed it, to update us on the status.

I look forward to receiving your revised manuscript.

Yours sincerely,

Lise Roth

Lise Roth, PhD
Senior Editor
EMBO Molecular Medicine

***** Reviewer's comments *****

Referee #1 (Remarks for Author):

This study proposes that, in EGFR-mutant NSCLC, osimertinib suppresses PPARGC1B (PGC1β)-dependent mitochondrial biogenesis and that reactivation of this pathway contributes to acquired osimertinib resistance. The mechanistic work is a clear strength, with relatively detailed interrogation of upstream regulation (including FOSL1/AP-1-mediated transcriptional control and links to hTERT). However, the clinical and biological positioning of the findings is currently limited by insufficient information on how the resistant sublines were generated and what established resistance mechanisms they harbor, making it difficult to determine whether PGC1β upregulation represents an independent resistance mechanism or a secondary adaptation in specific resistance contexts.

Major Comments

1. In Fig. 4A, multiple resistant sublines are used (PC-9/AR, PC-9/GR/AR, PC-9/3M, HCC827/AR), but critical information is missing or not clearly described in this manuscript: (i) how each resistant subline was generated (stepwise escalation vs. continuous exposure; duration; final drug concentration; maintenance conditions), and (ii) the meaning of the nomenclature (AR vs. GR/AR vs. 3M). Although these sublines appear to have been reported previously (Ref. 20), sufficient detail should be provided within this manuscript for readers to interpret the findings.

2. It is essential to clarify whether known resistance mechanisms are present in each subline (e.g., EGFR C797S, retention/loss of T790M, MET amplification, EGFR amplification, AXL activation, EMT, or histologic transformation). Given the heterogeneity of PC9-derived osimertinib resistance reported in the literature-including on-target mutations (C797S), bypass signaling (MET amplification), and ARID1A mutations identified as a resistance mechanism in PC9 cells (J Thorac Oncol 2025; 20(10):1459-1474)-the manuscript would benefit from explicitly positioning where the proposed PGC1β pathway fits relative to these established resistance states. Specifically, does PGC1β elevation occur independently, or does it co-occur with or depend on specific genetic drivers? Without this information, it is difficult to interpret whether PGC1β rebound represents a general resistance mechanism or a context-dependent adaptation accompanying specific resistance programs.

3.Fig. 4A shows increased PGC1β protein in resistant sublines. Please assess whether PPARGC1B mRNA is similarly elevated in these same resistant models. This is important to distinguish transcriptional regulation from potential post-transcriptional or protein-stability mechanisms, and it affects how the proposed regulatory axis should be therapeutically targeted.

4. The authors focus on PPARGC1B based on RNA-seq; it would strengthen the argument to present whether a broader mitochondrial biogenesis program is coordinately suppressed (e.g., a brief GSEA or a small qPCR panel including related regulators/targets such as PPARGC1A and mitochondrial biogenesis gene sets). This would support that the phenomenon reflects a pathway-level response rather than a single-gene change.

5. The study provides strong functional evidence that PPARGC1B overexpression blunts osimertinib-induced mitochondrial suppression and apoptosis, and that PPARGC1B knockdown restores sensitivity in resistant cells. To further sharpen the mechanistic interpretation, please expand the discussion (and, if feasible, provide additional data) on how preserved mitochondrial biogenesis/fitness mechanistically intersects with the survival/apoptosis machinery under EGFR inhibition. This is particularly relevant given the authors' own prior work demonstrating that Bim elevation and Mcl-1 suppression are critical mediators of osimertinib-induced apoptosis (Ref. 20). Discussion of metabolic rewiring (e.g., OXPHOS dependence) would also be informative.

6. In Fig. 3A-B, hTERT knockdown reduces total EGFR. In EGFR-mutant NSCLC, signaling output is more directly reflected by phospho-proteins. Please evaluate p-EGFR and downstream signaling (e.g., p-ERK and p-AKT) to support the conclusion that hTERT knockdown suppresses EGFR pathway activity rather than only total EGFR abundance.

Minor Comments

7.The schematic includes an ERK → c-Myc(S62) → TERT cascade. If this is not directly demonstrated in the current study, please add appropriate citations and/or modify the schematic to clearly distinguish established steps from proposed links. Additionally, a final integrated schematic that includes both sensitive and resistant states (and where PGC1β rebound occurs) would improve readability and should be considered for the Discussion.

8. In Fig. 4C-D, PGC1β elevation is reported in 12/21 (57.1%) post-relapse samples. If available, annotating these cases with known resistance mechanisms (e.g., C797S, MET amplification, etc.) would allow a more informative interpretation regarding correlation, mutual exclusivity, or subgroup enrichment with PGC1β elevation.

9.Because CPI-613 is being clinically tested largely in other cancer contexts (primarily pancreatic cancer), a brief discussion of feasibility for EGFR-mutant NSCLC would strengthen the translational section. This could include anticipated tolerability, dosing considerations, and how patients might be selected (e.g., by PGC1β elevation as a predictive biomarker).

Referee #2 (Comments on Novelty/Model System for Author):

The paper is technically sound, and the conclusions are largerly supported by the data. Whereas the osimertinib/CPI613 combination results are promising, the relevance to the described PGC1β is not a given, though, given the lack of a direct targeting strategy, the authors' approach and motivation are reasonable.

Referee #2 (Remarks for Author):

The manuscript by Chen at al describes the finding linking osimertinib-induced downregulation of PPARGC1B as an importatn component of osimertinib sensitivity, and demonstrate that restoration of PPARGC1B reduces osimertinib sensitivity. The manuscript is well written and clearly presented. The key conclusions are reasonably supported by the data.
There are several relatively minor issues that are worth addressing before the manuscript can be recommended for publication.
- The authors need to explain the rational for examining PPARGC1B. Osimertinib treatment reduces expression of large number of genes. What was the reasoning behind focusing on this gene in Figure 1?
- Whereas the data linking hTERT and PPARGC1B is reasonably convincing, the authors should at least speculate on the potential p53-independent mechanism that they have uncovered.
- Effects of osimertinib are not limited to suppression of mitochondrial biogenesis. They involve multiple additional dimmentions, including suppression of protein biogenesis, proliferation etc. While restoration of osimertinib sensitivity by suppression of PPARGCB1 is to be expected (in light of the findings described in this paper), the biological mechanism behind the loss of osimertinib sensitivity due to ectopic PPARGCB1 expression is puzzling, as it must entail the reversal of additional aspects of cellular responses to EGFR inhibition. Therefore, it would be informative to examine the impact of PARGCB1 expression on other key signaling nodes (PI3K-AKT-mTOR and JAK-STAT signaling), and at least speculate how restoration of a single aspect of EGFR inhibition is able to negate the effects on other important pathways.
- While the focus on apoptosis is justifiable, tumor relapse (or loss of responce) integrates proliferation and death. Authors imply that the suppression of tumor growth of osimertinib-CPI-613 combination is fully explainable by enhanced apoptosis. Authors also need to examine differences in cell proliferation (using KI67 staining of histology sections shown in Fig. 7I. Also, while the differences in BIM staining in fig. 7I support their inferences, without proper quantification the data is not sufficiently conclusive. Authors need to quantify the effect beyond showing a selected field of view. Alternatively, immunoblotting on protein lysates from tumors should provide a sufficiently informative independent validation.
Additional points:
- Plotting Fig S2C on log scale (for both axes) would be more useful for visual evaluation of the correlation, as most of the data points are clustered at low values.
- Page 18 ".. these effects were compromised in the corresponding...". "reduced" would be more informative and accurate than the "compromized"
- Page 19: expression of ectopic PPARGC1B confers resistance.." is somewhat of an overstatement. The effect is clear, but incomplete. Thus I suggest using "reduces inhibition" instead of "confers resistance". Conversely, given that PPARGC1B expression enabled net positive tumor growth under osimertinib, "confered resistance" would be appropriate vs. a weaker term "significantly reduced" used by authors is warranted.
- In 9 of 21 paired patient tissues, osimertinib resistance was not associated with PPARGC1B. While authors cover it in discussion, it could be highly informative to interrogate experimental models where osimertinib responces are not linked with PPARGC1B modulation. The relatively weak correlations reported in Figure S2 indicate that the findings reported here are not generalizable. While outside of the scope of the current paper, it will be important to elucidate the udnerlying biology.

Referee #3 (Remarks for Author):

This manuscript (EMM-2025-23215) examines PGC1β-driven mitochondrial biogenesis as a central determinant of sensitivity and acquired resistance to osimertinib in EGFR-mutant NSCLC. The authors report that osimertinib suppresses PGC1β through FOSL1/AP-1 and hTERT signaling, leading to impaired mitochondrial function, whereas resistant cells display restoration of PGC1β activity. Importantly, co-treatment with osimertinib and the mitochondrial inhibitor CPI-613 is shown to overcome resistance by inducing mitochondrial stress and apoptosis. However, the study would benefit from substantial additional revisions, including deeper mechanistic validation, expanded assessment of clinical relevance, and stronger support for the proposed therapeutic strategy.

Major suggestions:
(1) The authors are encouraged to demonstrate direct binding of FOSL1/AP-1 to the PPARGC1B promoter, such as through ChIP-qPCR analysis.
(2) To evaluate whether AP-1 overexpression can restore osimertinib sensitivity in a PGC1β-independent manner.
(3) The efficacy of osimertinib combined with CPI-613 against heterogeneous resistance (such as C797S and MET amplification) can be assessed by monitoring acquired resistance using continuous passaging or CRISPR screening.
(4) Conduct gain- and loss-of-function rescue experiments that specifically restore mitochondrial biogenesis (e.g., via ectopic overexpression of TFAM or MT-ND1) in resistant cells to demonstrate that PGC1β acts predominantly through this pathway, rather than through non-canonical functions such as lipid metabolism or ROS signaling.
(5) Confirm that hTERT regulates FOSL1 through ChIP-seq by demonstrating hTERT binding at the FOSL1 promoter, and determine whether telomerase inhibition (e.g., imetelstat) recapitulates PGC1β suppression in a mutant p53 context; additionally, explain why this pathway is retained in mutant p53 NSCLC cell lines despite reports that it is lost in p53-deficient models.
(6) Correlate pre- and post-treatment PGC1β IHC expression in larger EGFR-mutant NSCLC cohorts (n > 50) with progression-free survival on osimertinib, and validate circulating PGC1β in plasma as a pharmacodynamic biomarker using NCT05601633-like trials to inform patient selection for mitochondrial co-targeting.

Minor suggestions:
(1) The manuscript would benefit from PK/PD data supporting the in vivo doses used for osimertinib (5 mg/kg, OG) and CPI-613 (25 mg/kg, IP), including tumor/plasma exposure and bioavailability comparisons.
(2) Evaluate the potential of PGC1β/FOSL1 as biomarkers for patient stratification, incorporating TCGA correlation analyses and IHC cutoff criteria, while addressing limitations such as mutant p53 dependency and the lack of comparison with PGC1α.

Responses to reviewers' comments

We thank all reviewers for their valuable and constructive comments or recommendations that are very helpful for improving the manuscript. We carefully considered reviewers' comments and revised the manuscript by conducting additional experiments and further clarification. The revised parts were highlighted in red.

Referee #1 (Remarks for Author):

This study proposes that, in EGFR-mutant NSCLC, osimertinib suppresses PPARGC1B (PGC1β)-dependent mitochondrial biogenesis and that reactivation of this pathway contributes to acquired osimertinib resistance. The mechanistic work is a clear strength, with relatively detailed interrogation of upstream regulation (including FOSL1/AP-1-mediated transcriptional control and links to hTERT). However, the clinical and biological positioning of the findings is currently limited by insufficient information on how the resistant sublines were generated and what established resistance mechanisms they harbor, making it difficult to determine whether PGC1β upregulation represents an independent resistance mechanism or a secondary adaptation in specific resistance contexts.

Major Comments

1. In Fig. 4A, multiple resistant sublines are used (PC-9/AR, PC-9/GR/AR, PC-9/3M, HCC827/AR), but critical information is missing or not clearly described in this manuscript: (i) how each resistant subline was generated (stepwise escalation vs. continuous exposure; duration; final drug concentration; maintenance conditions), and (ii) the meaning of the nomenclature (AR vs. GR/AR vs. 3M). Although these sublines appear to have been reported previously (Ref. 20), sufficient detail should be provided within this manuscript for readers to interpret the findings.

As recommended, detailed information on these resistant cell lines had been added to the revision (Please see pages 28, last paragraph).

2. It is essential to clarify whether known resistance mechanisms are present in each subline (e.g., EGFR C797S, retention/loss of T790M, MET amplification, EGFR amplification, AXL activation, EMT, or histologic transformation). Given the heterogeneity of PC9-derived osimertinib resistance reported in the literature-including on-target mutations (C797S), bypass signaling (MET amplification), and ARID1A mutations identified as a resistance mechanism in PC9 cells (J Thorac Oncol 2025; 20(10):1459-1474)-the manuscript would benefit from explicitly positioning where the proposed PGC1β pathway fits relative to these established resistance states. Specifically, does PGC1β elevation occur independently, or does it co-occur with or depend on specific genetic drivers? Without this information, it is difficult to interpret

whether PGC1β rebound represents a general resistance mechanism or a context-dependent adaptation accompanying specific resistance programs.

In this study, PCG1β elevation was detected on osimertinib resistant cell lines irrespective of resistance mechanisms including C797S mutation (PC-9/3M) and MET amplification (HCC827/AR). Moreover, PGC1β elevation in EGFR-TKI resistant cell lines and tissues was not clearly associated with any new mutations. Hence, PGC1β elevation is unlikely to be a context-dependent adaptation accompanying specific resistance programs. Please see our revised descriptions page 12 (lines 3-6) and discussion on page 21 (lines 9-18). Gene mutation information in both cell lines and tissues has been included in the current Appendix Fig. S6 and Table S2.

3.Fig. 4A shows increased PGC1β protein in resistant sublines. Please assess whether PPARGC1B mRNA is similarly elevated in these same resistant models. This is important to distinguish transcriptional regulation from potential post-transcriptional or protein-stability mechanisms, and it affects how the proposed regulatory axis should be therapeutically targeted.

As suggested, we have generated new data indicating that *PPARGC1B* mRNA expression was also significantly increased in the tested osimertinib-resistant cell lines (please the revised Figure 5B and description on page 11, lines 3-7from bottom).

4. The authors focus on PPARGC1B based on RNA-seq; it would strengthen the argument to present whether a broader mitochondrial biogenesis program is coordinately suppressed (e.g., a brief GSEA or a small qPCR panel including related regulators/targets such as PPARGC1A and mitochondrial biogenesis gene sets). This would support that the phenomenon reflects a pathway-level response rather than a single-gene change.

As suggested, we have included GSEA data showing the downregulation of mitochondria gene set in both PC-9 and HCC827 cell lines (please see Appendix Fig. S1 and description on page 6, last paragraph).

5. The study provides strong functional evidence that PPARGC1B overexpression blunts osimertinib-induced mitochondrial suppression and apoptosis, and that PPARGC1B knockdown restores sensitivity in resistant cells. To further sharpen the mechanistic interpretation, please expand the discussion (and, if feasible, provide additional data) on how preserved mitochondrial biogenesis/fitness mechanistically intersects with the survival/apoptosis machinery under EGFR inhibition. This is particularly relevant given the authors' own prior work demonstrating that Bim elevation and Mcl-1 suppression are critical mediators of osimertinib-induced apoptosis (Ref. 20). Discussion of metabolic rewiring (e.g., OXPHOS dependence) would also be informative.

Ae suggested, we have added a discussion on the connection between suppression of mitochondrial biogenesis, particularly mitochondrial ROS production, and Bim-dependent induction of apoptosis in both sensitive and osimertinib-resistant EGFRm NSCLC cells (please see the 2nd paragraph on page 23). Meanwhile, we have added a schema illustrating this mechanistic connection (Please see Appendix Fig. S7).

6. In Fig. 3A-B, hTERT knockdown reduces total EGFR. In EGFR-mutant NSCLC, signaling output is more directly reflected by phospho-proteins. Please evaluate p-EGFR and downstream signaling (e.g., p-ERK and p-AKT) to support the conclusion that hTERT knockdown suppresses EGFR pathway activity rather than only total EGFR abundance.

As suggested, we have included the data on p-EGFR, p-ERK and p-Akt in the revised Fig. 4 (A and B) and described the results on page 11 (line 6-8).

Minor Comments

7.The schematic includes an ERK → c-Myc(S62) → TERT cascade. If this is not directly demonstrated in the current study, please add appropriate citations and/or modify the schematic to clearly distinguish established steps from proposed links. Additionally, a final integrated schematic that includes both sensitive and resistant states (and where PGC1β rebound occurs) would improve readability and should be considered for the Discussion.

As suggested, the initial figure 3F (current Fig. 4G) and the according legend have been revised with citation of previous publications.

8. In Fig. 4C-D, PGC1β elevation is reported in 12/21 (57.1%) post-relapse samples. If available, annotating these cases with known resistance mechanisms (e.g., C797S, MET amplification, etc.) would allow a more informative interpretation regarding correlation, mutual exclusivity, or subgroup enrichment with PGC1β elevation.

As suggested, we have included gene mutations in these tissues as Appendix Table S2. No C797S or MET amplification was detected. As described in this revision, we did not see a clear association between PGC1β elevation and novel mutations (see page 12, lines 3-6).

9.Because CPI-613 is being clinically tested largely in other cancer contexts (primarily pancreatic cancer), a brief discussion of feasibility for EGFR-mutant NSCLC would strengthen the translational section. This could include anticipated tolerability, dosing considerations, and how patients might be selected (e.g., by PGC1β elevation as a predictive biomarker).

As suggested, we have added a brief discussion in this revision on page 18 (the first paragraph,

lines 5-8 from the bottom).

Referee #2 (Comments on Novelty/Model System for Author):

The paper is technically sound, and the conclusions are largerly supported by the data. Whereas the osimertinib/CPI613 combination results are promising, the relevance to the described PGC1β is not a given, though, given the lack of a direct targeting strategy, the authors' approach and motivation are reasonable.

Referee #2 (Remarks for Author):

The manuscript by Chen at al describes the finding linking osimertinib-induced downregulation of PPARGC1B as an importatn component of osimertinib sensitivity, and demonstrate that restoration of PPARGC1B reduces osimertinib sensitivity. The manuscript is well written and clearly presented. The key conclusions are reasonably supported by the data.

There are several relatively minor issues that are worth addressing before the manuscript can be recommended for publication.

- The authors need to explain the rational for examining PPARGC1B. Osimertinib treatment reduces expression of large number of genes. What was the reasoning behind focusing on this gene in Figure 1?

The rationale for studying PPARGGC1B has been described in the introduction of the manuscript (pages 4 and 5). The major reason for us to pick up PGC1β because it is a coactivator for the transcriptional factors, SREBP1 and PPARγ that have been linked to mediation of osimertinib's therapeutic efficacy and acquired resistance as demonstrated in our previous work (Chen et al. 2021) and ongoing work (PPARγ).

- Whereas the data linking hTERT and PPARGC1B is reasonably convincing, the authors should at least speculate on the potential p53-independent mechanism that they have uncovered.

As revealed in our study, the p53-independent regulation of *PPARGC1B* expression by hTERT is mediated by FOSL. We generated all data presented in the initial Fig. 3 (current Fig. 4) to demonstrate this mechanism or connection.

- Effects of osimertinib are not limited to suppression of mitochondrial biogenesis. They involve multiple additional dimmentions, including suppression of protein biogenesis, proliferation etc. While restoration of osimertinib sensitivity by suppression of PPARGCB1 is to be expected (in light of the findings described in this paper), the biological mechanism behind the loss of

osimertinib sensitivity due to ectopic PPARGCB1 expression is puzzling, as it must entail the reversal of additional aspects of cellular responses to EGFR inhibition. Therefore, it would be informative to examine the impact of PARGCB1 expression on other key signaling nodes (PI3K-AKT-mTOR and JAK-STAT signaling), and at least speculate how restoration of a single aspect of EGFR inhibition is able to negate the effects on other important pathways.

The finding on PGC1β elevation as a resistant mechanism or osimertinib in this study does not exclude other important resistant mechanisms. The expression of ectopic *PPARGC1B* only partially attenuated the effects on suppression of mitochondrial biogenesis, induction of apoptosis, and particularly suppression of tumor growth. Some mechanisms are likely to be associated with the same axis via altering upstream and downstream events or feed-forward mechanisms. Under this scenario, blockage of one event may have a profound consequence. The future study may address the connection of PGC1β with other osimertinib resistance mechanisms.

- While the focus on apoptosis is justifiable, tumor relapse (or loss of responce) integrates proliferation and death. Authors imply that the suppression of tumor growth of osimertinib-CPI-613 combination is fully explainable by enhanced apoptosis. Authors also need to examine differences in cell proliferation (using KI67 staining of histology sections shown in Fig. 7I. Also, while the differences in BIM staining in fig. 7I support their inferences, without proper quantification the data is not sufficiently conclusive. Authors need to quantify the effect beyond showing a selected field of view. Alternatively, immunoblotting on protein lysates from tumors should provide a sufficiently informative independent validation.

As suggested, we have stained Ki67 and added the results in the revised Fig. 9. Moreover, we have included Western blotting data from the same tumors in Fig. EV5 showing similar alterations of Bim and cPARP. Moreover, we have included results on p-EGFR and p-ERK alterations. These results were described on page 17, last 9 lines in the first paragraph.

Additional points:
- Plotting Fig S2C on log scale (for both axes) would be more useful for visual evaluation of the correlation, as most of the data points are clustered at low values.

The initial Fig. S2C has been replaced with log scales (please see the revised Appendix Fig. S2C in this revision).

- Page 18 ".. these effects were compromised in the corresponding...". "reduced" would be more informative and accurate than the "compromized"

As suggested, this wording has been changed (see the revised page 12).

- Page 19: expression of ectopic PPARGC1B confers resistance.." is somewhat of an overstatement. The effect is clear, but incomplete. Thus I suggest using "reduces inhibition" instead of "confers resistance". Conversely, given that PPARGC1B expression enabled net positive tumor growth under osimertinib, "confered resistance" would be appropriate vs. a weaker term "significantly reduced" used by authors is warranted.

Changes have been made as suggested (please see the revised page 12)

- In 9 of 21 paired patient tissues, osimertinib resistance was not associated with PPARGC1B. While authors cover it in discussion, it could be highly informative to interrogate experimental models where osimertinib responces are not linked with PPARGC1B modulation. The relatively weak correlations reported in Figure S2 indicate that the findings reported here are not generalizable. While outside of the scope of the current paper, it will be important to elucidate the udnerlying biology.

We totally agree and recognized this fact as stated in the discussion as "Given the heterogenous mechanisms accounting for osimertinib acquired resistance, it can be logically speculated that other resistance mechanisms may play dominant roles in EGFRm NSCLCs relapsed from osimertinib treatment in which PGC1β expression is not increased." (page 21 in this revision). Our lab has been working hard to identify additional resistance mechanisms.

Referee #3 (Remarks for Author):

This manuscript (EMM-2025-23215) examines PGC1β-driven mitochondrial biogenesis as a central determinant of sensitivity and acquired resistance to osimertinib in EGFR-mutant NSCLC. The authors report that osimertinib suppresses PGC1β through FOSL1/AP-1 and hTERT signaling, leading to impaired mitochondrial function, whereas resistant cells display restoration of PGC1β activity. Importantly, co-treatment with osimertinib and the mitochondrial inhibitor CPI-613 is shown to overcome resistance by inducing mitochondrial stress and apoptosis. However, the study would benefit from substantial additional revisions, including deeper mechanistic validation, expanded assessment of clinical relevance, and stronger support for the proposed therapeutic strategy.

Major suggestions:
(1) The authors are encouraged to demonstrate direct binding of FOSL1/AP-1 to the PPARGC1B promoter, such as through ChIP-qPCR analysis.

We have oligo pull down results indicating direct binding of FOSL1/AP-1 to the PPARGC1B promoter. Please see data presented in the initial Fig. 2N (current Fig. 3E).

(2) To evaluate whether AP-1 overexpression can restore osimertinib sensitivity in a PGC1β-independent manner.

The current study focused on PGC1β /mitochondrial biogenesis in mediating therapeutic efficacy of osimertinib in EGFRm NSCLC cells. Although this study demonstrates a now role of FOSL1/AP-1 in regulation PPARGC1B expression, AP-1 is known to have many other targeted genes. We speculate that AP-1 overexpression may restore osimertinib sensitivity in a PGC1β-independent manner, which is beyond the scope of this study and will be explored in the future. In the initial submission, we also recognized that directly targeting AP-1 may cause potential side effects of toxicity since it regulates various biological processes via different target genes in the discussion (please see 22, last paragraph in this revision).

(3) The efficacy of osimertinib combined with CPI-613 against heterogeneous resistance (such as C797S and MET amplification) can be assessed by monitoring acquired resistance using continuous passaging or CRISPR screening.

The combination was effective in HCC827/AR cell line that has MET amplification. However, this combination was not effective in PC-9/3M that carries a C797S mutation given that the C797S mutation prevents osimertinib from binding to the activating mutant EGFR (see the revised Fig. EV4C and description on page 16, lines 6-8). To be clear, we have added Appendix Table S1 that summarizes the characteristics of the EGFRm cell lines used in this study to this revision.

(4) Conduct gain- and loss-of-function rescue experiments that specifically restore mitochondrial biogenesis (e.g., via ectopic overexpression of TFAM or MT-ND1) in resistant cells to demonstrate that PGC1β acts predominantly through this pathway, rather than through non-canonical functions such as lipid metabolism or ROS signaling.

As suggested, we conducted the rescue experiment using overexpression of ectopic TFAM inn both PC-9/AR and HCC827/AR cells as presented in the revised Appendix Fig. S4. The findings show that enforced overexpression of ectopic *TFAM* partially rescued mitochondrial dysfunction and apoptosis induced by PGC1β suppression/osimertinib treatment in these osimertinib-resistant cells. These results further demonstrate a critical role of mitochondrial suppression in mediating osimertinib's therapeutic efficacy. Please see our description of the results on page 14, 2nd paragraph for detail.

(5) Confirm that hTERT regulates FOSL1 through ChIP-seq by demonstrating hTERT binding at the FOSL1 promoter, and determine whether telomerase inhibition (e.g., imetelstat) recapitulates

PGC1β suppression in a mutant p53 context; additionally, explain why this pathway is retained in mutant p53 NSCLC cell lines despite reports that it is lost in p53-deficient models.

We must be clear that this study does not study hTERT directly regulation of FOSL1 expression. Rather, we demonstrate that hTERT regulates FOSL1 levels via EGFR/EGFR signaling as presented in Fig. 3F (the current Fig. 4G). It is currently unclear whether hTERT had a direct effect on regulation of FOSL1 levels.

We also checked the effects of Imetelstat on EGFR, FOSL1 and PGC1β in both PC-9 and HCC827 cells and found that it did not alter the levels of these proteins (the revised Fig. 4F), suggesting that hTERT regulates EGFR/FOSL1/PGC1β axis via a telomerase-independent mechanisms. Our findings are different from p53-dependent regulation of PGC1β, which is dependent on telomerase and telomere. This may explain why hTERT regulates the EGFR/FOSL1/PGC1β axis in cells with mutant p53. Please see the description on page 10, the last paragraph.

(6) Correlate pre- and post-treatment PGC1β IHC expression in larger EGFR-mutant NSCLC cohorts ($n > 50$) with progression-free survival on osimertinib, and validate circulating PGC1β in plasma as a pharmacodynamic biomarker using NCT05601633-like trials to inform patient selection for mitochondrial co-targeting.

We thank the reviewer for this recommendation; while we agree that obtaining tumor biopsy at baseline and post-progression on osimertinib to look for PGC1β by IHC would be informative, there are some practical limitations. We recently published a manuscript showing that obtaining on-treatment biopsy even in academic centers is challenging for lung cancer patients (Piotrowska Z et al, Cancer Discovery 2026); in this multi-center study specifically designed to obtain biopsy at the time of disease progression across prominent academic centers, only 40% of the patients were able to undergo this. We will therefore pursue an effort to use circulating tumor cells as a potential vehicle to pursue pharmacodynamic studies in this patient population in the future when it is practical in the clinic.

Minor suggestions:

(1) The manuscript would benefit from PK/PD data supporting the in vivo doses used for osimertinib (5 mg/kg, OG) and CPI-613 (25 mg/kg, IP), including tumor/plasma exposure and bioavailability comparisons.

The recommendation has been well taken and will be considered in our future studies. The current in vivo study focused on evaluating the therapeutic efficacy of osimertinib and CPI-613 against EGFRm tumors with acquired resistance to osimertinib. In this revised Fig. EV5, the

combination clearly decreased the levels of p-EGFR and p-ERK with increased levels of Bim, which could serve as potential PD markers for therapeutic efficacy of the combination.

(2) Evaluate the potential of PGC1β/FOSL1 as biomarkers for patient stratification, incorporating TCGA correlation analyses and IHC cutoff criteria, while addressing limitations such as mutant p53 dependency and the lack of comparison with PGC1α.

Evaluation of PGC1β as a potential biomarker patient stratification has been discussed (see pages 18 and 21). Given that patients with EGFRm NSCLC harboring a p53 co-mutation is highly prevalent and generally associated with a poorer survival in EGFR-targeted therapy, the combinational strategy of osimertinib with CPI-613 may be particularly suitable for the treatment of this type of patients, particularly those whose tumors express elevated PGC1β (please see our discussion on page 22, the first paragraph).

We did not consider PGC1α in our study because its gene expression was undetectable and altered by osimertinib in both PC-9 and HCC827 cells based on our RNA-seq data. This has been mentioned in this revision (page5, lines 4-5) as part of the rationale for our focus on PGC1β.

21st Jun 2026

Dear Dr. Chen,

Thank you for submitting your revised manuscript, which has now been evaluated by referees #1 and #3. As you will see below, they are overall satisfied with the revisions, and I will therefore be able to accept your manuscript once the following points have been addressed:

1/ Please address the remaining comments raised by referee #1.

2/ Manuscript text:
- Please remove the red font text and only keep in track changes mode any new modification.
- Please remove "Request for reprints" on the title page and define the corresponding author(s) instead.
- Remove the reagent table from the manuscript text and upload it as an independent file.
- In the methods, please avoid over-reliance on previously published articles, and provide sufficient details to ensure reproducibility of the experiments. Additionally, please provide the following information:
o Cell lines: indicate the origin of the cells and their culture conditions, and state if they were authenticated.
o Western blots: provide more details, including loading controls, number of replicates, whether the membranes were stripped, as well as antibody dilutions.
o Human NSCLC tissues: provide more experimental details. Include a statement confirming that informed consent was obtained from all subjects, and the full statement that the experiments conformed to the principles set out in the WMA Declaration of Helsinki and the Department of Health and Human Services Belmont Report. State details of authority granting ethics approval (IRB or equivalent committee(s), provide reference number for approval.
o Animal experiments: provide origin, strain, sex, age, genetic modification status. Include a statement of compliance with ethical regulations. Provide reference number for ethic approval.
o RNA sequencing: clarify whether RNAseq was performed, and provide detailed methods. The obtained datasets should be deposited in a public repository and the
o Statistics: provide statements about sample size, blinding, randomization, and inclusion/exclusion criteria.
- Data Availability: please see above (RNA sequencing). Primary datasets produced in this study need to be deposited in an appropriate public database, and the accession numbers and database listed under 'Data Availability'. If the study doesn't contain newly generated data that necessitate deposition in a public database, please only indicate: "This study contains no data deposited in external repositories."
- Please rename "Disclosure of potential conflicts of interest" to "Disclosure and competing interests statement".
- Acknowledgements: ensure that all funding sources are listed in our system in addition to the Acknowledgements, as they will be linked in PubMed (i.e. should the Winship Cancer Institute of Emory University internal funds and the David A Cole Professorship fund be added as funding sources in our system?)
- Remove the authors contributions from the manuscript text, and provide CRediT (Contributor Role Taxonomy) terms in the submission system instead.
- References: Please refer to our guidelines for citing preprints and correct where needed (i.e. Korotkevich G, Sukhov V, Budin N, Shpak B, Artyomov MN, Serushichev A (2021) Fast gene set enrichment analysis. BioRxiv): https://link.springer.com/journal/44321/submission-guidelines#cms-Reference-guidelines

3/ Figures:
- Please note that the resolution of the submitted figures is too low for blot and microscopy images. This reduction in resolution is commonly caused by converting original 16-bit TIFF files to RGB format for publication. While this is not inherently problematic, it can raise concerns about image integrity for critical readers.
To avoid any misunderstanding and to meet EMBO Press standards, we kindly ask that you resubmit the complete figure set, including the Appendix file at the captured original data resolution.
- Please add callouts for the panels of Fig EV3 and EV5.
- Please correct the heading "EV figure legends" to "Expanded View Figure Legends".
- Please note that we can accommodate more than five Expanded View (EV) figures. We thus encourage you to convert some or all of your current Appendix figures into EV figures to improve accessibility for readers. If you choose to keep an Appendix, please compile the appendix figures and table into one PDF file, and a table of contents should be added to the file, with page numbers.
- Please address the queries from our data editors in the figure legends:
1. Please note that the legends for figures 2; 5; 9 are not provided in the sequential manner (legend for figure 2G is provided before legend of figure 2F; legend for figure 5C is provided before legend of figure 5B; legend for figure 9B,C are provided before legend of figure 9D, etc.). This needs to be rectified.
2. Please note that the figure 5D is mislabeled as figure 5C, respectively in the manuscript. This needs to be rectified.
3. Please note that the exact p values are not provided in the legends of 1A,B,J; 2F; 3C,D; 5B; 6B,C,E,F; 7B,D,F,H; 8B,D,I,J; 9A,C,D,F; EV-3B,C; EV-5B.
4. Please indicate the statistical test used for data analysis in the legends of figure 1A,B,J; 2F; EV-3B,C.

5. Please note that information related to n is missing in the legends 2F; EV-5B.
6. Please note that the error bars are not defined in the legends of 2F; EV-5B.

4/ Checklist: please fill in the section 'Experimental study design and statistics/inclusion-exclusion criteria'.

5/ Source Data:
Thank you for providing source data. We encourage you to provide the additional Western blot replicates.

6/ The paper explained: EMBO Molecular Medicine articles are accompanied by a summary of the articles to emphasize the major findings in the paper and their medical implications for the non-specialist reader. Please provide a draft summary of your article highlighting
- the medical issue you are addressing,
- the results obtained and
- their clinical impact.
This may be edited to ensure that readers understand the significance and context of the research. Please refer to any of our published articles for an example.

7/ Every published paper includes a 'Synopsis' to further enhance discoverability. Synopses are displayed on the journal webpage and are freely accessible to all readers. They include a short stand first (maximum of 300 characters, including space) as well as 2-5 one-sentences bullet points that summarizes the paper. Please write the bullet points to summarize the key NEW findings. They should be designed to be complementary to the abstract - i.e. not repeat the same text. We encourage inclusion of key acronyms and quantitative information (maximum of 30 words / bullet point). Please use the passive voice. Please attach these in a separate file or send them by email, we will incorporate them accordingly.

Please also suggest a striking image or visual abstract to illustrate your article as a PNG file 550 px wide x 300 px high.

8/ As part of the EMBO Publications transparent editorial process initiative (see our Editorial at http://embomolmed.embopress.org/content/2/9/329), EMBO Molecular Medicine will publish online a Review Process File (RPF) to accompany accepted manuscripts.
This file will be published in conjunction with your paper and will include the anonymous referee reports, your point-by-point response and all pertinent correspondence relating to the manuscript. Let us know whether you agree with the publication of the RPF.
Please note that the Authors checklist will be published at the end of the RPF.

I look forward to receiving your revised manuscript.

Yours sincerely,

Lise Roth

Lise Roth, PhD
Senior Editor
EMBO Molecular Medicine

***** Reviewer's comments *****

Referee #1 (Remarks for Author):

I thank the authors for a thorough revision. My original comments 1, 3, 4, 5, 6, 8, and 9 are adequately addressed. Two points remain partially addressed, plus minor corrections.

Comment 2
The authors show that PGC1β elevation is independent of the genetic alterations detectable by their NGS panel, which I appreciate. However, the "general resistance mechanism" claim (p.21) overreaches. The non-genetic mechanisms the authors themselves highlight in the Introduction (EMT, AXL activation, histologic transformation) cannot be captured by a mutation panel

and were not assessed, and a "general" claim also sits uneasily with the 57.1% (12/21) frequency the authors report. I ask the authors either to assess one or two non-genetic markers (e.g., E-cadherin/Vimentin or ZEB1, {plus minus} AXL) in the existing resistant sublines, or to temper the wording (e.g., "a recurrent/common mechanism") and state the non-genetic mechanisms as an explicit limitation.

Comment 7
The added citations in the Fig. 4G legend are appreciated, but a single integrated schematic covering both sensitive and resistant states (and where PGC1β rebound occurs) is still lacking. I suggest elevating Appendix Fig. S7 to serve this role, or adding a brief composite to Fig. 4G.

Editorial corrections. TFMA → TFAM (p.14); CPI-693 → CPI-613 (p.22); HCC8277/AR → HCC827/AR (p.21); PPARGGC1B → PPARGC1B (text/figures); Fig. 5 legend lists "C" twice and omits "D".

Referee #3 (Remarks for Author):

The authors have adequately addressed the comments.

Responses to reviewer #1's comments

We thank the reviewer again for the constructive comments. Based on the recommendations, we have made corrections in this version.

Referee #1 (Remarks for Author):

I thank the authors for a thorough revision. My original comments 1, 3, 4, 5, 6, 8, and 9 are adequately addressed. Two points remain partially addressed, plus minor corrections.

Comment 2
The authors show that PGC1β elevation is independent of the genetic alterations detectable by their NGS panel, which I appreciate. However, the "general resistance mechanism" claim (p.21) overreaches. The non-genetic mechanisms the authors themselves highlight in the Introduction (EMT, AXL activation, histologic transformation) cannot be captured by a mutation panel and were not assessed, and a "general" claim also sits uneasily with the 57.1% (12/21) frequency the authors report. I ask the authors either to assess one or two non-genetic markers (e.g., E-cadherin/Vimentin or ZEB1, {plus minus} AXL) in the existing resistant sublines, or to temper the wording (e.g., "a recurrent/common mechanism") and state the non-genetic mechanisms as an explicit limitation.

As recommended, we have reworded the expression as "a common mechanism" and mentioned "Whether other non-genetic resistance mechanisms impact PGC1β rebound elevation in osimertinib-resistant cells/tissues may need further investigation" (please see page 21, lines 2-3 from the bottom).

Comment 7
The added citations in the Fig. 4G legend are appreciated, but a single integrated schematic covering both sensitive and resistant states (and where PGC1β rebound occurs) is still lacking. I suggest elevating Appendix Fig. S7 to serve this role, or adding a brief composite to Fig. 4G.

By the time we called out Fig. 4, the findings on rebound elevation of PGC1β in osimertinib-resistant cells/tissues and its connection to FOSL1 had not been described (Fig. 5). This was the main reason why the schema in the previous Fig. 4G did not include the situations in the resistant cells. Nonetheless, we have revised the Fig. 4G by adding possible mechanism of PGC1β elevation in the resistant cells as suggested.

Additional minor revisions

In addition, we made a minor textual clarification in the revised manuscript to indicate that the RNA-seq data shown in Fig. 1A were from our previously generated RNA-seq dataset in EGFR-mutant NSCLC cells treated with osimertinib.

We also reanalyzed the flow cytometry data in Fig. 7B, Fig. 8I, and Fig. 8J based on three independent experiments and updated the corresponding P values in the revised figures and figure legends. These updates do not affect the overall interpretation or conclusions of the study.

15th Jul 2026

Dear Dr. Chen,

Thank you for submitting your revised files. I am pleased to inform you that your manuscript is accepted for publication and is now being sent to our publisher to be included in the next available issue of EMBO Molecular Medicine.

Your manuscript will be processed for publication by EMBO Press. It will be copy edited and you will receive page proofs prior to publication. Please note that you will be contacted by Springer Nature Author Services to complete licensing and payment information.

You may qualify for financial assistance for your publication charges - either via a Springer Nature fully open access agreement or an EMBO initiative. Check your eligibility: https://link.springer.com/journal/44321/how-to-publish-with-us

Should you be planning a Press Release on your article, please notify the production team via this form in order to coordinate publication and release dates: https://forms.cloud.microsoft/e/eH4A8JVP6g

If you have any questions, please do not hesitate to contact the Editorial Office.
Thank you for your contribution to EMBO Molecular Medicine.

Yours sincerely,

Lise Roth

Lise Roth, Ph.D
Senior Editor
EMBO Molecular Medicine

>>> Please note that it is EMBO Molecular Medicine policy for the transcript of the editorial process (containing referee reports and your response letter) to be published as an online supplement to each paper. If you do NOT want this, you will need to inform the Editorial Office via email immediately. More information is available here: https://link.springer.com/partners/embo-press/editorial-policies#Peer%20review

EMBO Press Author Checklist

Corresponding Author Name: Shi-yong Sun	
Journal Submitted to: EMBO Molecular Medicine	
Manuscript Number: EMM-2025-23215	

USEFUL LINKS FOR COMPLETING THIS FORM
The EMBO Journal - Author Guidelines
EMBO Reports - Author Guidelines
Molecular Systems Biology - Author Guidelines
EMBO Molecular Medicine - Author Guidelines

Reporting Checklist for Life Science Articles (updated January
This checklist is adapted from Materials Design Analysis Reporting (MDAR) Checklist for Authors. MDAR establishes a minimum set of requirements in transparent reporting in the life sciences (see Statement of Task: 10.31222/osf.io/9sm4x). Please follow the journal's guidelines in preparing your
Please note that a copy of this checklist will be published alongside your article.

Abridged guidelines for figures
1. Data
The data shown in figures should satisfy the following conditions:
- the data were obtained and processed according to the field's best practice and are presented to reflect the results of the experiments in an accurate and unbiased manner.
- ideally, figure panels should include only measurements that are directly comparable to each other and obtained with the same assay.
- plots include clearly labeled error bars for independent experiments and sample sizes. Unless justified, error bars should not be shown for technical
- if n<5, the individual data points from each experiment should be plotted. Any statistical test employed should be justified.
- Source Data should be included to report the data underlying figures according to the guidelines set out in the authorship guidelines on Data

2. Captions
Each figure caption should contain the following information, for each panel where they are relevant:
- a specification of the experimental system investigated (eg cell line, species name).
- the assay(s) and method(s) used to carry out the reported observations and measurements.
- an explicit mention of the biological and chemical entity(ies) that are being measured.
- an explicit mention of the biological and chemical entity(ies) that are altered/varied/perturbed in a controlled manner.
- the exact sample size (n) for each experimental group/condition, given as a number, not a range;
- a description of the sample collection allowing the reader to understand whether the samples represent technical or biological replicates (including how many animals, litters, cultures, etc.).
- a statement of how many times the experiment shown was independently replicated in the laboratory.
- definitions of statistical methods and measures:
  - common tests, such as t-test (please specify whether paired vs. unpaired), simple χ2 tests, Wilcoxon and Mann-Whitney tests, can be unambiguously identified by name only, but more complex techniques should be described in the methods section;
  - are tests one-sided or two-sided?
  - are there adjustments for multiple comparisons?
  - exact statistical test results, e.g., P values = x but not P values < x;
  - definition of 'center values' as median or average;
  - definition of error bars as s.d. or s.e.m.

Please complete ALL of the questions below.
Select "Not Applicable" only when the requested information is not relevant for your study.

Materials

Newly Created Materials	Information included in the manuscript?	In which section is the information available? (Reagents and Tools Table, Materials and Methods, Figures, Data Availability Section)
New materials and reagents need to be available; do any restrictions apply?	Yes	Reagents and Tools Table; Materials and Methods

Antibodies	Information included in the manuscript?	In which section is the information available? (Reagents and Tools Table, Materials and Methods, Figures, Data Availability Section)
For **antibodies** provide the following information:<br>- Commercial antibodies: RRID (if possible) or supplier name, catalogue number and or/clone number<br>- Non-commercial: RRID or citation	Yes	Reagents and Tools Table; Materials and Methods

DNA and RNA sequences	Information included in the manuscript?	In which section is the information available? (Reagents and Tools Table, Materials and Methods, Figures, Data Availability Section)
**Short novel DNA or RNA including primers, probes**: provide the sequences.	Yes	Materials and Methods

Cell materials	Information included in the manuscript?	In which section is the information available? (Reagents and Tools Table, Materials and Methods, Figures, Data Availability Section)
**Cell lines:** Provide species information, strain. Provide accession number in repository **OR** supplier name, catalog number, clone number, and/**OR** RRID.	Yes	Materials and Methods
**Primary cultures:** Provide species, strain, sex of origin, genetic modification status.	Not Applicable	
Report if the cell lines were recently **authenticated** (e.g., by STR profiling) and tested for mycoplasma contamination.	Yes	Materials and Methods

Experimental animals	Information included in the manuscript?	In which section is the information available? (Reagents and Tools Table, Materials and Methods, Figures, Data Availability Section)
**Laboratory animals or Model organisms:** Provide species, strain, sex, age, genetic modification status. Provide accession number in repository **OR** supplier name, catalog number, clone number, **OR** RRID.	Yes	Reagents and Tools Table; Materials and Methods
**Animal observed in or captured from the field:** Provide species, sex, and age where possible.	Not Applicable	
Please detail **housing and husbandry conditions**.	Yes	Materials and Methods

Plants and microbes	Information included in the manuscript?	In which section is the information available? (Reagents and Tools Table, Materials and Methods, Figures, Data Availability Section)
**Plants:** provide species and strain, ecotype and cultivar where relevant, unique accession number if available, and source (including location for collected wild specimens).	Not Applicable	
**Microbes:** provide species and strain, unique accession number if available, and source.	Not Applicable	

Human research participants	Information included in the manuscript?	In which section is the information available? (Reagents and Tools Table, Materials and Methods, Figures, Data Availability Section)
If collected and within the bounds of privacy constraints report on age, sex and gender or ethnicity for all study participants.	Not Applicable	

Core facilities	Information included in the manuscript?	In which section is the information available? (Reagents and Tools Table, Materials and Methods, Figures, Data Availability Section)
If your work benefited from core facilities, was their service mentioned in the acknowledgments section?	Yes	Acknowledgments

Design

Study protocol	Information included in the manuscript?	In which section is the information available? (Reagents and Tools Table, Materials and Methods, Figures, Data Availability Section)
If study protocol has been **pre-registered, provide DOI in the manuscript**. For clinical trials, provide the trial registration number **OR** cite DOI.	Not Applicable	
Report the **clinical trial registration number** (at ClinicalTrials.gov or equivalent), where applicable.	Not Applicable	

Laboratory protocol	Information included in the manuscript?	In which section is the information available? (Reagents and Tools Table, Materials and Methods, Figures, Data Availability Section)
Provide DOI OR other citation details if **external detailed step-by-step protocols** are available.	Not Applicable	

Experimental study design and statistics	Information included in the manuscript?	In which section is the information available? (Reagents and Tools Table, Materials and Methods, Figures, Data Availability Section)
Include a statement about **sample size** estimate even if no statistical methods were used.	Yes	Figure Legends; Materials and Methods
Were any steps taken to minimize the effects of subjective bias when allocating animals/samples to treatment (e.g. **randomization procedure**)? If yes, have they been described?	Yes	Materials and Methods
Include a statement about **blinding** even if no blinding was done.	Yes	Materials and Methods
Describe **inclusion/exclusion criteria** if samples or animals were excluded from the analysis. Were the criteria pre-established?<br><br>If sample or data points were omitted from analysis, report if this was due to attrition or intentional exclusion and provide justification.	Not Applicable	
For every figure, are **statistical tests** justified as appropriate? Do the data meet the assumptions of the tests (e.g., normal distribution)? Describe any methods used to assess it. Is there an estimate of variation within each group of data? Is the variance similar between the groups that are being statistically compared?	Yes	Figure Legends; Materials and Methods

Sample definition and in-laboratory replication	Information included in the manuscript?	In which section is the information available? (Reagents and Tools Table, Materials and Methods, Figures, Data Availability Section)
In the figure legends: state number of times the experiment was **replicated** in laboratory.	Yes	Figure Legends
In the figure legends: define whether data describe **technical or biological replicates**.	Yes	Figure Legends

Ethics

Ethics	Information included in the manuscript?	In which section is the information available? (Reagents and Tools Table, Materials and Methods, Figures, Data Availability Section)
Studies involving **human participants**: State details of **authority granting ethics approval** (IRB or equivalent committee(s), provide reference number for approval.	Yes	Materials and Methods
Studies involving **human participants**: Include a statement confirming that **informed consent** was obtained from all subjects and that the experiments conformed to the principles set out in the WMA Declaration of Helsinki and the Department of Health and Human Services Belmont Report.	Yes	Materials and Methods
Studies involving **human participants:** For publication of **patient photos**, include a statement confirming that consent to publish was obtained.	Not Applicable	
Studies involving experimental **animals**: State details of **authority granting ethics approval** (IRB or equivalent committee(s), provide reference number for approval. Include a statement of compliance with ethical regulations.	Yes	Materials and Methods
Studies involving **specimen and field samples:** State if relevant **permits** obtained, provide details of authority approving study; if none were required, explain why.	Not Applicable	

Dual Use Research of Concern (DURC)	Information included in the manuscript?	In which section is the information available? (Reagents and Tools Table, Materials and Methods, Figures, Data Availability Section)
Could your study fall under dual use research restrictions? Please check biosecurity documents and list of **select agents and toxins** (CDC): https://www.selectagents.gov/sat/list.htm	Not Applicable	
If you used a select agent, is the security level of the lab appropriate and reported in the manuscript?	Not Applicable	
If a study is subject to dual use research of concern regulations, is the name of the **authority granting approval and reference number** for the regulatory approval provided in the manuscript?	Not Applicable	

Reporting

The MDAR framework recommends adoption of discipline-specific guidelines, established and endorsed through community initiatives. Journals have their own policy about requiring specific guidelines and recommendations to complement MDAR.

Adherence to community standards	Information included in the manuscript?	In which section is the information available? (Reagents and Tools Table, Materials and Methods, Figures, Data Availability Section)
State if relevant guidelines or checklists (e.g., **ICMJE, MIBBI, ARRIVE, PRISMA**) have been followed or provided.	Yes	Materials and Methods
For **tumor marker prognostic studies,** we recommend that you follow the **REMARK** reporting guidelines (see link list at top right). See author guidelines, under 'Reporting Guidelines'. Please confirm you have followed these guidelines.	Not Applicable	
For **phase II and III randomized controlled trials**, please refer to the **CONSORT** flow diagram (see link list at top right) and submit the CONSORT checklist (see link list at top right) with your submission. See author guidelines, under 'Reporting Guidelines'. Please confirm you have submitted this list.	Not Applicable	

Data Availability

Data availability	Information included in the manuscript?	In which section is the information available? (Reagents and Tools Table, Materials and Methods, Figures, Data Availability Section)
Have **primary datasets** been deposited according to the journal's guidelines (see 'Data Deposition' section) and the respective accession numbers provided in the Data Availability Section?	Not Applicable	
Were **human clinical and genomic datasets** deposited in a public access-controlled repository in accordance to ethical obligations to the patients and to the applicable consent agreement?	Not Applicable	
Are **computational models** that are central and integral to a study available without restrictions in a machine-readable form? Were the relevant accession numbers or links provided?	Not Applicable	
If publicly available data were reused, provide the respective **data citations in the reference list**.	Yes	Materials and Methods
